# DFT Modeling of Organocatalytic Ring-Opening Polymerization of Cyclic Esters: A Crucial Role of Proton Exchange and Hydrogen Bonding

**DOI:** 10.3390/polym11122078

**Published:** 2019-12-12

**Authors:** Ilya Nifant’ev, Pavel Ivchenko

**Affiliations:** 1 Chemistry Department, M.V. Lomonosov Moscow State University, 1 Leninskie Gory Str., Building 3, 119991 Moscow, Russia; 2 A.V. Topchiev Institute of Petrochemical Synthesis RAS, 29 Leninsky Pr., 119991 Moscow, Russia

**Keywords:** density functional theory, lactones, lactides, cyclic carbonates, cyclic phosphates, ring-opening polymerization, organocatalysis

## Abstract

Organocatalysis is highly efficient in the ring-opening polymerization (ROP) of cyclic esters. A variety of initiators broaden the areas of organocatalysis in polymerization of different monomers, such as lactones, cyclic carbonates, lactides or gycolides, ethylene phosphates and phosphonates, and others. The mechanisms of organocatalytic ROP are at least as diverse as the mechanisms of coordination ROP; the study of these mechanisms is critical in ensuring the polymer compositions and architectures. The use of density functional theory (DFT) methods for comparative modeling and visualization of organocatalytic ROP pathways, in line with experimental proof of the structures of the reaction intermediates, make it possible to establish these mechanisms. In the present review, which continues and complements our recent manuscript that focused on DFT modeling of coordination ROP, we summarized the results of DFT modeling of organocatalytic ROP of cyclic esters and some related organocatalytic processes, such as polyester transesterification.

## 1. Introduction

**Figure 1:**
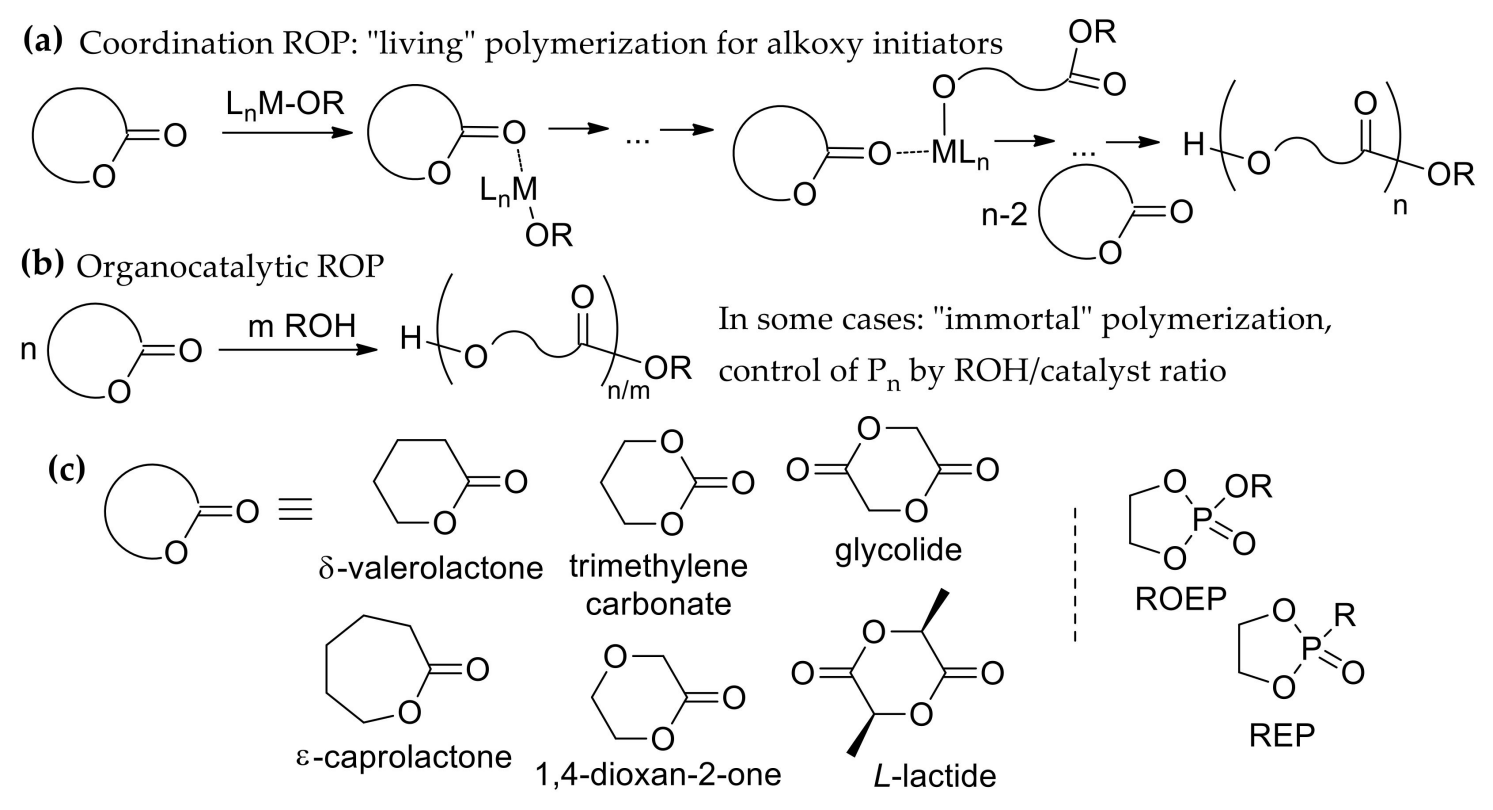
Coordination (**a**) and organocatalytic (**b**) ring-opening polymerization (ROP); (**c**) cyclic esters, monomers for ROP.

The development of biotechnology and medical technology has set higher requirements for biomedical materials. The catalytic ring-opening polymerization (ROP) of cyclic esters and related compounds (Figure 1) provides a basis of the efficient synthesis of biodegradable and biocompatible polymers [1,2,3,4,5,6,7,8]. Tin (II) carboxylates [9,10,11,12] or aluminium alkoxides [13,14,15,16] were the first efficient ROP catalysts, in the next decades hundreds of metal complexes were studied in the polymerization of different cyclic substrates [1,2,17,18]. However, consideration must be given to the fundamental difference between metal-catalyzed industrial processes, such as Ziegler-Natta polymerization of α-olefins and ROP of cyclic esters. The monomer-to-catalyst molar ratio for the first process is typically in the range 10^6^–10^8^ [19]; therefore, the removal of the catalyst is not needed and polyolefins can be processed without purification. Instead, the synthesis of polyesters via the ROP of cyclic esters typically requires the monomer-to-catalyst ratios ~10^3^–10^4^ [1,2,20]. The high oxophilicity of the metal complexes makes their removal from oxygen-rich polyesters difficult. Tackling the problem of the metal catalyst removal has become all the more crucial for sensitive domains, such as biomedical, packaging, and microelectronics [21]. The ability of organocatalysts to be effectively removed from the polymers is a significant asset. The other benefits of organocatalysts include mild reaction conditions, the option to vary initiator/catalyst ratios, and lower toxicity [22,23,24,25]. That is why the use of organocatalysts appears to be a reliable alternative to the coordination of ROP catalysts in the synthesis of biomedical-grade polymers.

To date, various organocatalysts of ROP have been studied [22,23,24,25,26,27,28]. The density functional theory (DFT) methods have been applied for the modeling of the organocatalytic ROP to improve the understanding of the reaction mechanisms for different cyclic substrates, to determine the scope and limitations of ROP in the development of biodegradable polymers with given compositions and architectures. Houk et al. reviewed DFT modeling of the full diversity of organocatalytic processes in 2011 [29], the recent review of Jones [30] that addressed the contributions of quantum chemistry to the development of ROP also looked at some of the aspects of organocatalytic polymerization of cyclic esters. Ruipérez discussed some aspects of catalytic ROP in a recent review [31]. Traditionally, organocatalytic ROP mechanisms are divided into base-catalyzed and acid-catalyzed [22,23,25,27], but it became clear in recent years that such a split is rather conditional.

**Figure 2:**
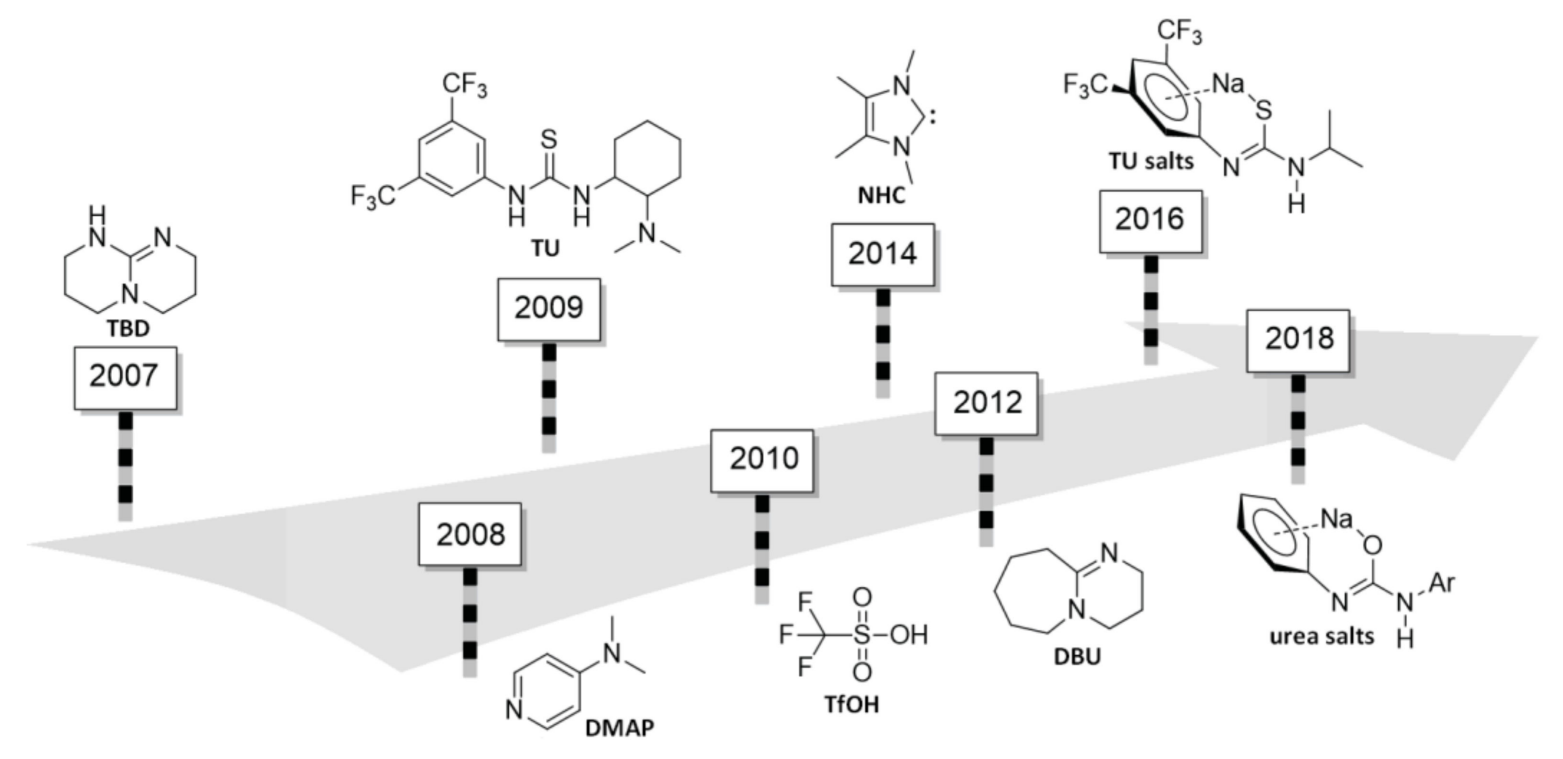
Chronological compilation of the types of ROP organocatalysts studied by the density functional theory (DFT) method.

In the present review, we tried to summarize and analyze the results of DFT modeling of organocatalytic ROP reported in dozens of publications. In line with different types of organocatalysts (Figure 2), the material that is presented in the review is divided into six main parts bearing in mind the retrospective of the each research topics. This review complements our previous work that was devoted to the DFT modeling of coordination ROP that was recently published in the special issue of *Molecules* [32].

## 2. Polymerizability of Cyclic Esters

The ROP thermodynamics plays a crucial role for polymerizability of cyclic esters, as mentioned in a number of publications and reviewed in [33]. In our recent review [32], we only mentioned this subject; in the present manuscript we consider the reaction ability of cyclic esters in more detail.

**Figure 3:**
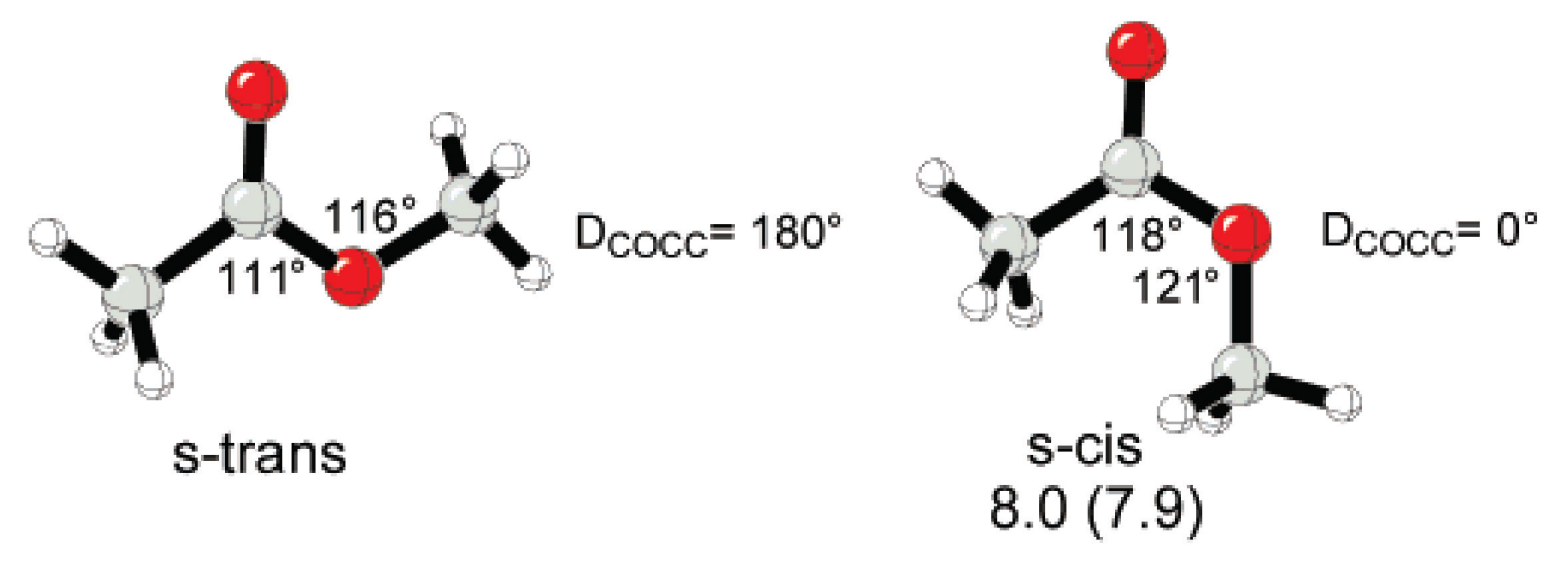
Geometries of *s*-trans and *s*-cis methyl acetate and relative Gibbs free energies (kcal/mol) of *s*-cis methyl acetate determined experimentally and calculated (in parentness). Reprinted with permission from [37]. Copyright (2008) American Chemical Society.

The strain of the lactone ring can be attributed to the higher stability of *s*-trans conformation of esters relative to *s*-cis-conformation [34], which was confirmed by DFT optimization of methyl acetate conformers [35,36,37] (Figure 3).

The strain energies for five- and six-membered lactones, γ-butyrolactone (γBL), and δ-valerolactone (δVL) were found to be ~8 kcal/mol (calorimetry data) [38]. Although this might be thought to be more than enough to cause the polymerization of these lactones, γBL does not polymerize under the typical ROP conditions, in contrast with easily polymerizable δVL. Houk et al. studied the thermochemistry of γBL and δVL ROP, and the conformational preferences of model molecules that mimic corresponding polymers, to explain these facts [37]. The geometry optimizations were preformed at B3LYP/6-31G(d) level [39,40,41]; the CBS-QB3 method [42,43] was used for additional calculations of thermodynamic parameters. The calculations of the thermochemistry of the model transesterification reaction between the lactones and methyl acetate when given the values of ΔG as 1.0 and −1.3 kcal/mol for γBL and δVL, respectively. The conformation effects in the transesterification products also significantly contribute to the thermochemistry of the reactions [44,45]; a high preference for gauche-coiled conformations of the transesterification products for polymerizable lactones was also detected. 

Aleman et al. performed later work on the polymerizability of 1,4-dioxan-2-one (PDO), ε-caprolactone (εCL) [46], glycolide (GL), *L*-lactide (*l*-LA), and (*R*,*S*)-lactide [47]. All of these monomers were found to be polymerizable due to strain in the ester group. In addition, the predominance of high-energy coiled conformations in linear homopolymers the formed from PDO, εCL and GL was proportionately less than extended conformations, in comparison to the inactive monomer γBL. However, while the prevalence of coiled conformations over extended conformations was expected for poly(LA), this factor does not affected the reactivity of lactides. Very good agreement between the experimental and calculated data for ROP of GL, *l*-LA and (*R*,*S*)-lactide [47] should be separately noted.

## 3. 4-(Dimethylamino)pyridine (DMAP) and Related Compounds

**Scheme 1:**
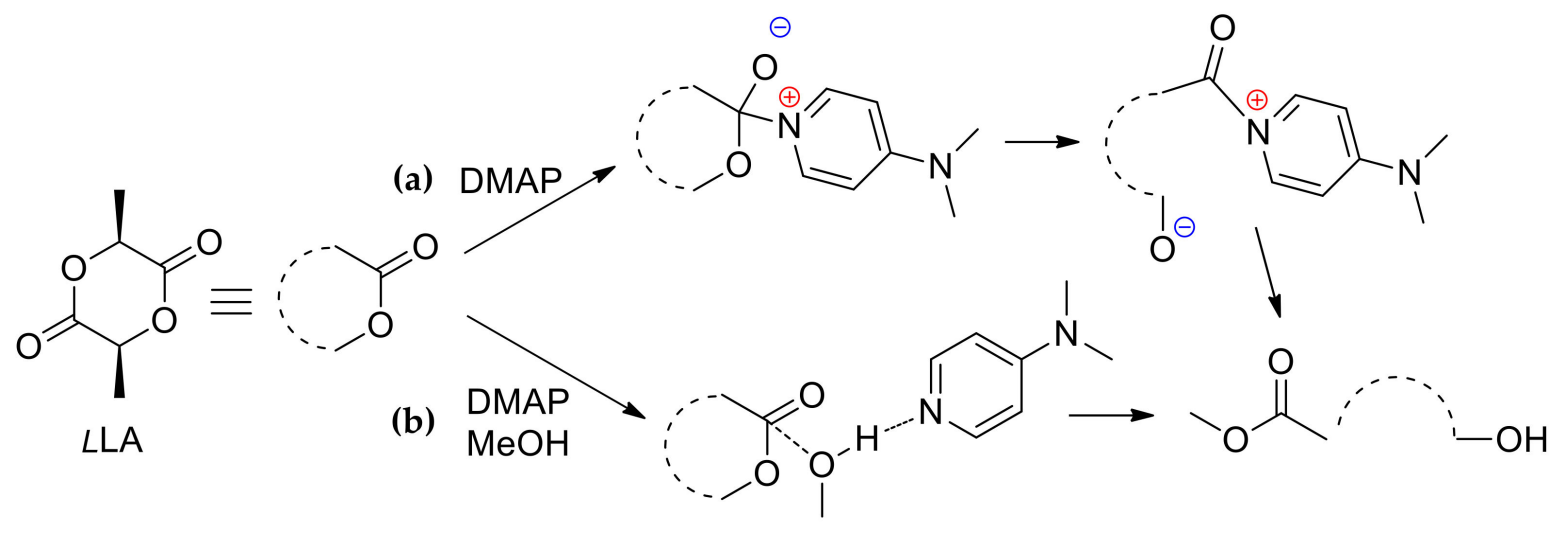
Schematic representation of the nucleophilic/monomer (**a**) and basic/alcohol (**b**) mechanisms for (Dimethylamino)pyridine (DMAP)-catalyzed methanolysis of *l*-LA.

DMAP was the first employed organocatalyst in polymer synthesis. In 2001, Nederberg et al. [48] reported the “first organocatalytic living polymerization” (*Ð_M_* < 1.2) of *L*-lactide (*l*-LA) mediated by DMAP or 4-pyrrolidinopyridine in the presence of ROH initiators. Four years later [49], Feng and Dong reported polymerization of ε-caprolactone (εCL) catalyzed by DMAP in the presence of chitozane as an initiator. Bourissou et al. studied the mechanism of DMAP-catalyzed ROP of *l*-LA and five-membered lactic O-carboxylic anhydride (lacOCA) [50]. Two possible reaction pathways were analyzed at the B3LYP/6–31G(d) level of theory [39,40,51]; the solvent effect of dichloromethane was taken into account through the PCM/SCRF model [52]. The modeled reaction was the methanolysis of *l*-LA or lacOCA; alternative nucleophilic/monomer (Scheme 1a) and basic/alcohol (Scheme 1b) activation mechanisms were proposed for these reactions, and then analyzed by DFT optimization of the key reaction intermediates and transition states, if necessary.

**Figure 4:**
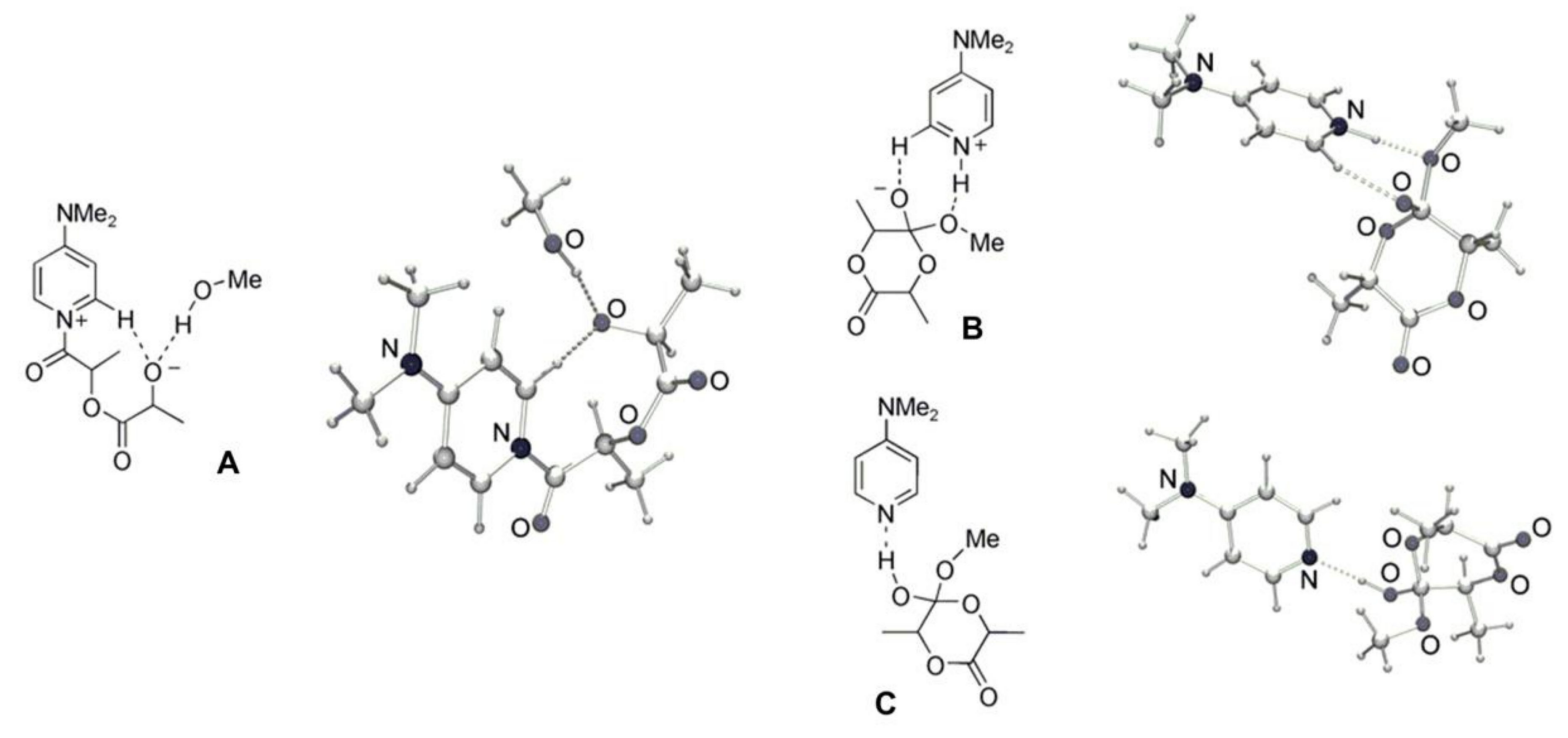
Optimized structures for the acylpyridinium intermediate **A** (nucleophilic/monomer mechanism) and tetrahedral intermediates **B** and **C** (basic/alcohol mechanism). Reprinted with permission from [50]. Copyright (2008) Wiley-VCH Verlag GmbH & Co.

The first, the zwitterionic nucleophilic/monomer reaction pathway was found to be energetically unfavorable: the key reaction acylpyridinium intermediate **A** (Figure 4) was 25 kcal/mol higher in energy than the separated reactants, thus suggesting a fairly large activation barrier. The detailed analysis of the reaction profile for nucleophilic/monomer mechanism was not implemented while taking substantially lower activation barriers for the basic/alcohol reaction pathway into account.

**Figure 5:**
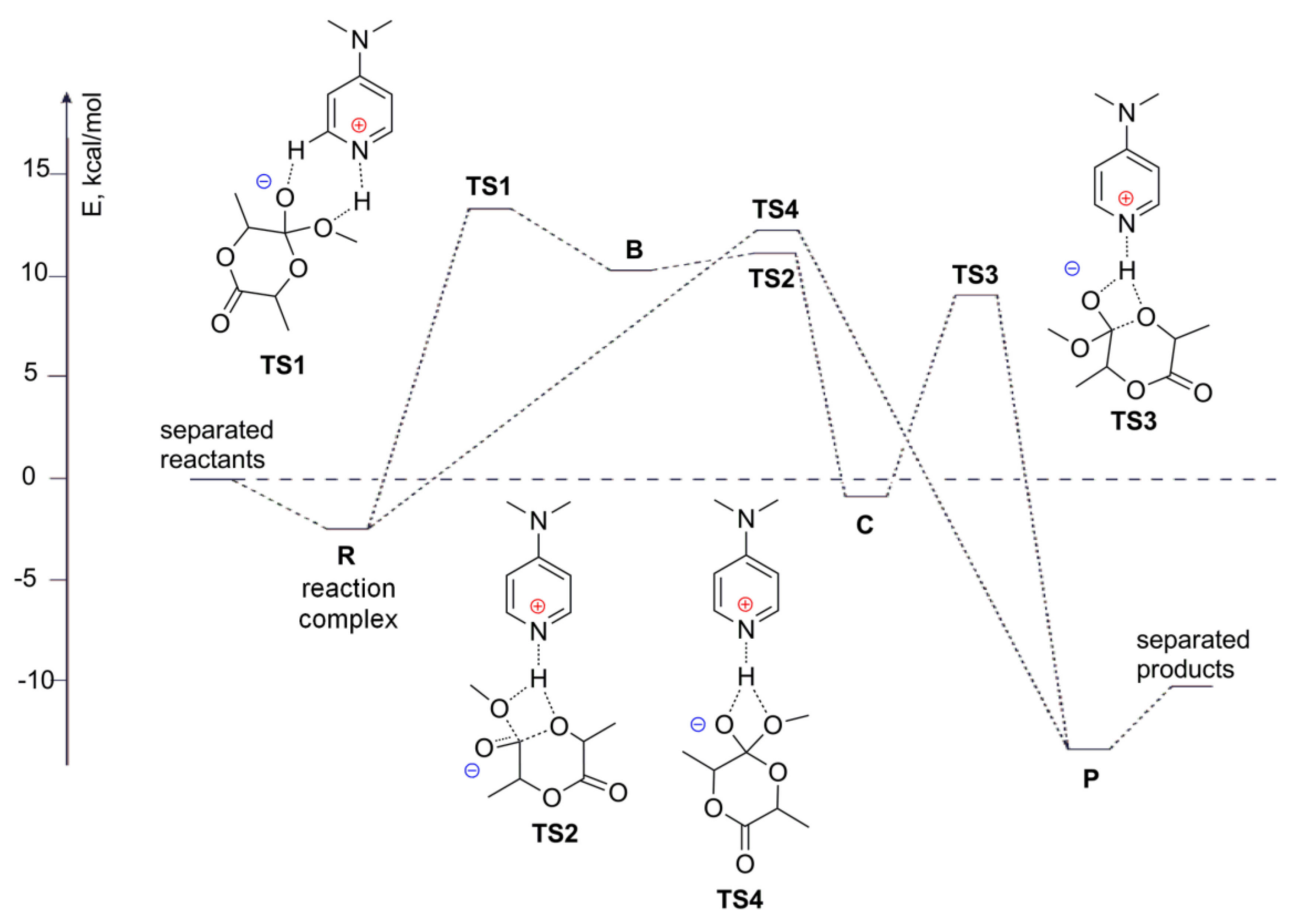
The energy profile (E - electronic energies) of the basic/alcohol mechanism of the DMAP-catalyzed ROP of *l*-LA with MeOH as an initiator. Reprinted with permission from [50]. Copyright (2008) Wiley-VCH Verlag GmbH & Co.

The key reaction intermediates for the basic/alcohol reaction pathway **B** and **C** (Figure 4) represent zwitterionic and neutral complexes that are stabilized by hydrogen bonding. The intermediate **B** was approximately 10 kcal/mol higher in energy than the separated reactants. The neutral complex **C** was more stable than **B** by ~20 kcal/mol in the gas phase; this difference was lower (~10 kcal/mol) in dichloromethane because of the stabilization of the more polar structure **B**. Figure 5 presents the whole energy profile for the basic/alcohol reaction pathway. 

**Figure 6:**
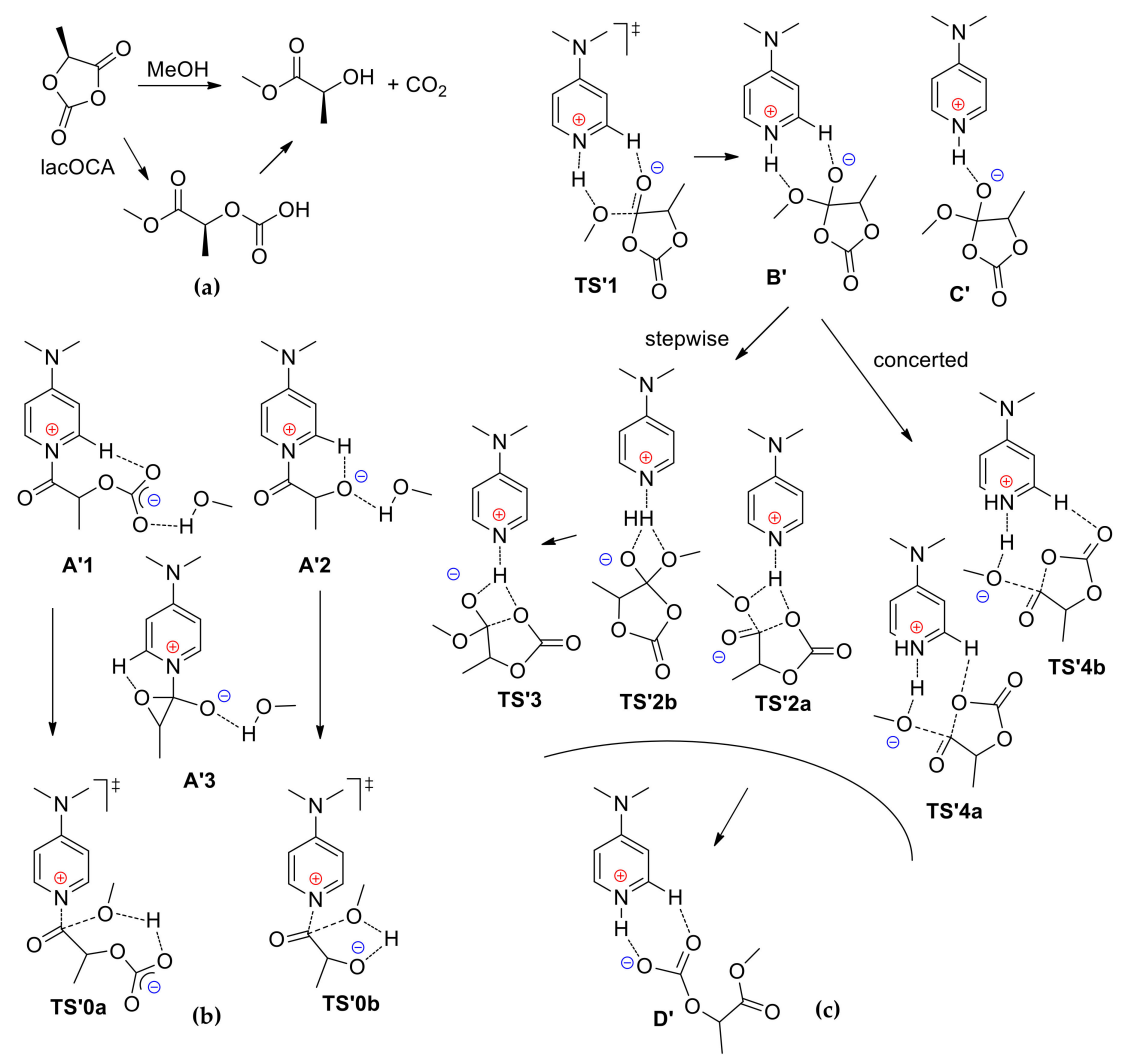
Methanolysis of lactic O-carboxylic anhydride (lacOCA) (**a**); Intermediates and transition states of the reaction for nucleophilic/monomer (**b**) and basic/alcohol (**c**) mechanisms [50].

The transition states TS1 and TS2, which connect the zwitterionic intermediate **B** to the ternary complex of reactants **R** and the neutral intermediate **C**, respectively, were located. TS1 and TS2 were both very close in energy to **B**, with barrier heights of only a few kcal/mol. In addition, **C** connected with the products **P** via TS3, with a barrier height of approximately 10 kcal/mol in CH_2_Cl_2_. The possibility of a concerted ring-opening reaction of *l*-LA with MeOH was also estimated, and the corresponding transition state TS4 was indeed found to directly connect **R** with **P** (Figure 5). The barrier height calculated for TS4 was comparable to the barrier that was predicted for the stepwise reaction (TS1), thus suggesting that the ROP might occur in a stepwise or concerted manner (Figure 5). It was found that lacOCA methanolysis (Figure 6a), which models the ROP of l-lacOCA, was more favorable than *l*-LA methanolysis. The decomposition occurs over two stages; the ring-opening step provides the major part of the enthalpic term, whereas the decarboxylation step plays a key role entropically.

In the framework of the nucleophilic/monomer mechanism, the key stationary points and transition states (Figure 6b) were found. In contrast with that observed for *l*-LA, acylpyridinium complex **A’1** was only a few kcal/mol higher in energy than reactants **R**. The product of decarboxylation **A’2** and its isomer **A’3** were predicted to be very close in energy. In the transition states of MeOH insertion in **A’1** and **A’2** (TS’0a and TS’0b, respectively) the nucleophilic attack of methanol on the activated carbonyl group was intramolecularly assisted by the carbonate or alkoxide groups, and **TS’0b** was slightly favored energetically over TS’0a. 

However, basic/alcohol mechanism was found to be preferable for lacOCA, similarly to *l*-LA. The intermediates **B’** and **C’** (Figure 6c) of the lacOCA methanolysis by basic/alcohol mechanism resembled the intermediates **B** and **C** of *l*-LA reaction, but they were more energetically accessible. The formation of the ring-opened intermediate **D’** that precedes the final decarboxylation step was predicted to be energetically favorable by ~15 kcal/mol. The whole energy profile for lacOCA methanolysis was created and the reaction sequence included a number of transition states that were stabilized by hydrogen bonding (Figure 6c). It was found that the highest transition state for the basic/alcohol mechanism (TS’1) laid significantly lower in energy than the most favorable transition state for the nucleophilic/monomer mechanism (TS’0b), the difference in the activation barriers of these two pathways exceeded 15 kcal/mol in electronic energies and 9 kcal/mol in the Gibbs free energies. From these results, it was proposed that DMAP-catalyzed ROP of lacOCA occurs by the activation of the alcohol through hydrogen bonding, rather than nucleophilic activation of the cyclic substrate. In addition, the ring-opening of lacOCA was predicted to simultaneously proceed through a stepwise or concerted pathway (TS’2–3 and TS’4, respectively, Figure 6c). The analysis of the reaction of this cyclic substrate with methyl lactate instead of MeOH refined the reaction model for lacOCA ROP; the results of calculations were in line with the results that were obtained for the simple MeOH model.

The possibility of DMAP acting both as a hydrogen bond acceptor (through its basic nitrogen center) and a weak hydrogen bond donor (through one *ortho*-hydrogen atom) was the fundamental finding of the work [50]. Such behaviour was highly similar to the catalyst action of the 1,5,7-triazabicyclo[4.4.0]dec-5-ene (TBD) and other basic catalysts that are discussed in Section 4, Section 5, Section 6 and Section 7.

**Scheme 2:**
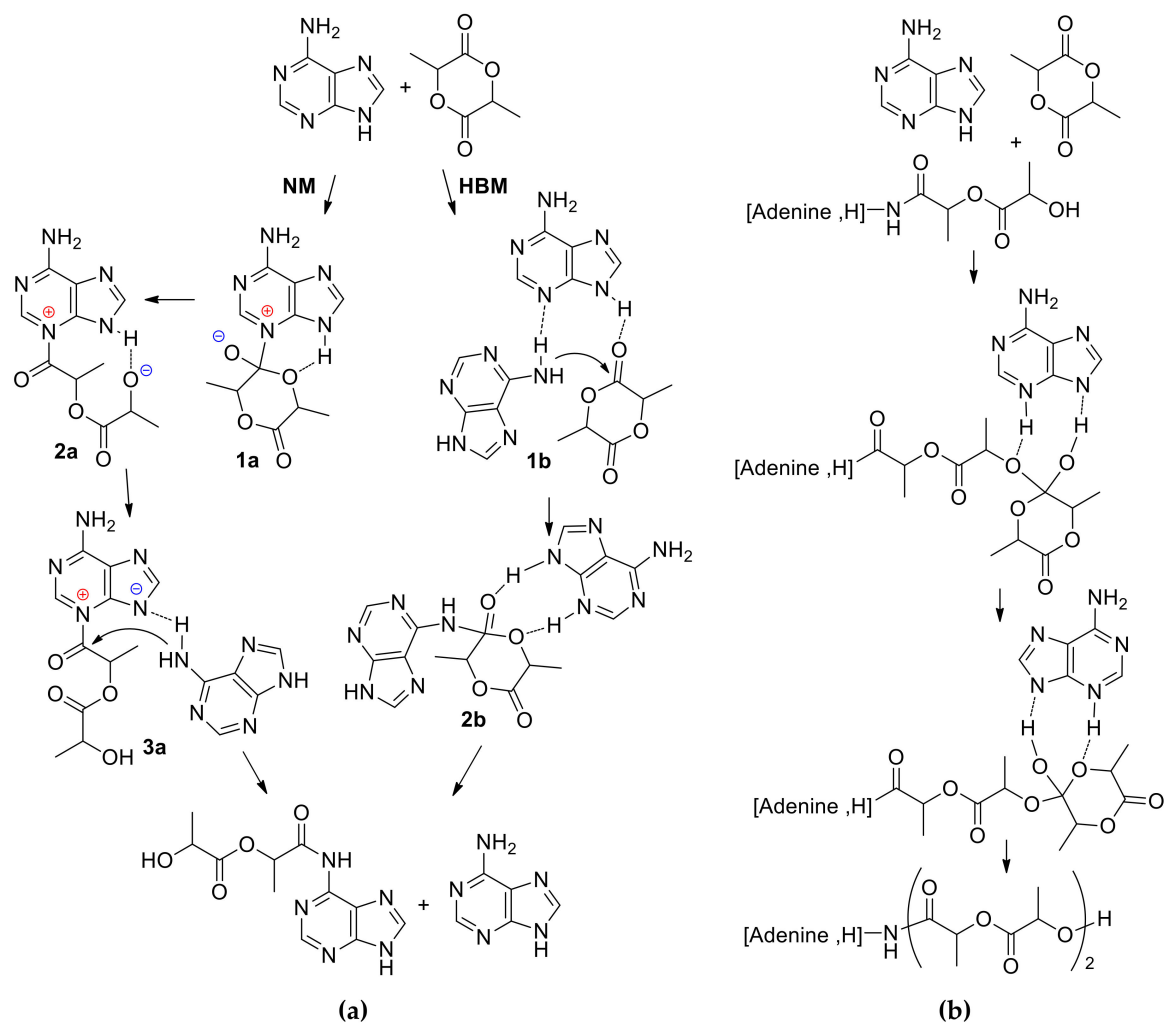
(**a**) Nucleophilic mechanism (NM) and hydrogen-bonding mechanism (HBM) of the initiation stage of adenine-catalyzed *l*-LA ROP; (**b**) Propagation stage in the frameworks of HBM [53].

Zinck et al. found some similarities with DMAP-catalyzed ROP of cyclic esters in the polymerization of *l*-LA catalyzed by adenine without additional initiators [53]. The formation of adenine-functionalized PLA was detected, and this experimental fact was explained while using the results of DFT modeling at M06-2X/6-31+G(d,p) [51,54] level of theory. Two alternative reaction mechanisms, namely, nucleophilic (NM) and hydrogen-bonding (HBM) were analyzed. In NM, adenine inserts into the *l*-LA carbonyl group (1a–2a, Scheme 2a), the hydrogen bonding of the adjacent nitrogen to a second incoming adenine (3a, Scheme 2a) completes the cycle to form the adenine end-capped ring-opened LA. In the first step of HBM, the hydrogen that was attached to the nitrogen of the first adenine molecule activates the >C=O of *l*-LA through hydrogen bonding, and the imine nitrogen simultaneously activates the amine group of a second adenine molecule by attracting the hydrogen through a lone pair interaction (1b, Scheme 2a). The activated amine group attacks the electrophilic carbon of the carbonyl group of *l*-LA, giving tetrahedral intermediate (2b, Scheme 2a). The subsequent ring opening results in the formation of the adenine end-capped ring-opened LA and the release of an adenine molecule.

**Figure 7:**
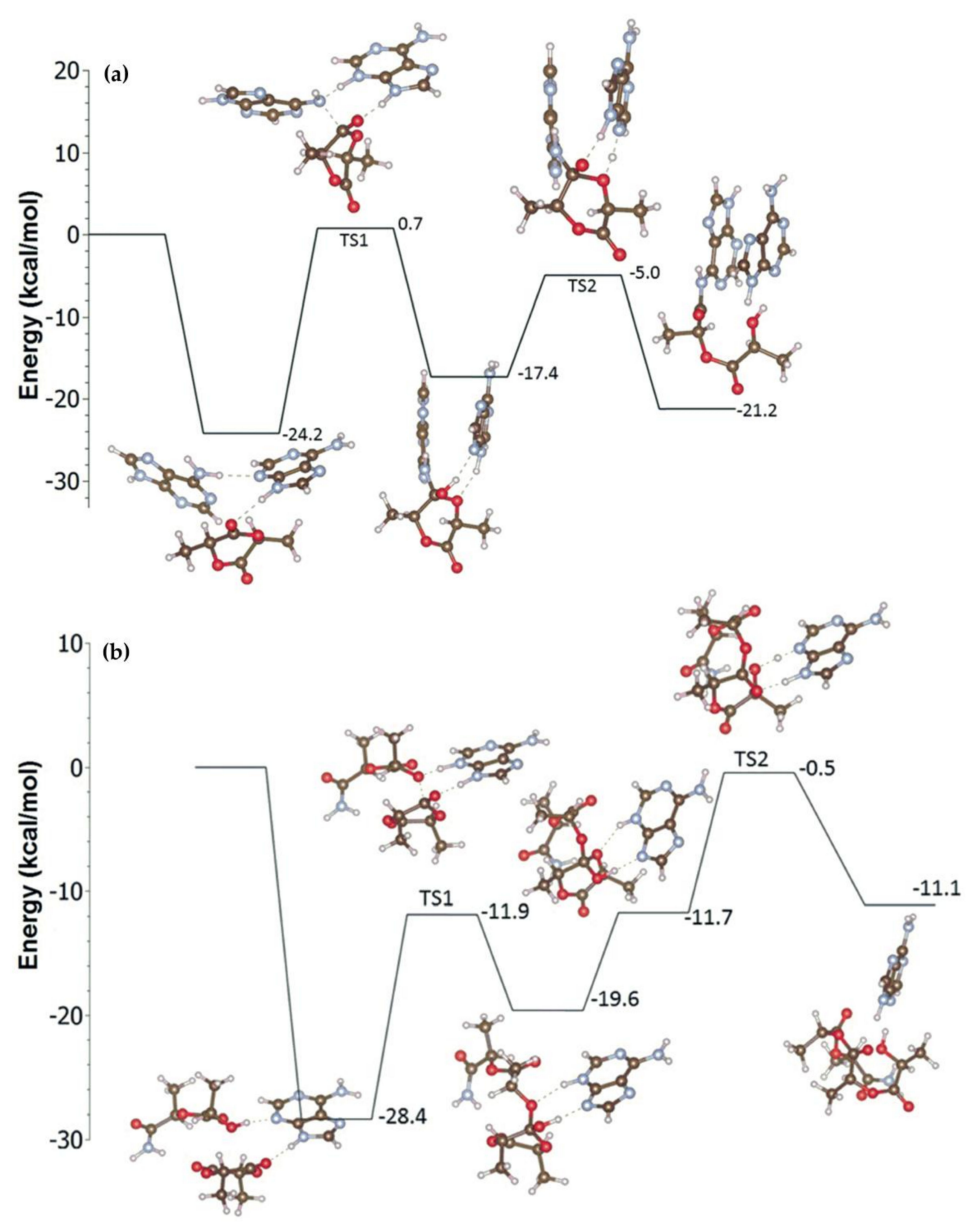
The reaction energy profiles (electronic energies with zero-point corrections) for initiation (**a**) and propagation (**b**) stages of adenine-catalyzed *l*-LA ROP. Reprinted with permission from [53]. Copyright (2016) Royal Society of Chemistry.

The lack of success in the search of the key insertion transition state was one of the notable results of the comparative modeling of *l*-LA ROP initiation in the frameworks of NM and HBM, resulting in stationary point **1a** (NM, Scheme 2a). A hydrogen bond interaction was detected between the (imidazole)N–H and the carbonyl group, and this interaction inhibited the desirable interaction between this bond and exocyclic oxygen atom and prevented the nucleophilic attack of the imine nitrogen to the carbonyl. Thus, the HBM mechanism was found to be more plausible, and the propagation stage (Scheme 2b) was also studied; amide by the formula H_2_NC(O)CHMeOC(O)CHMeOH mimicking adenine end-capped ring-opened LA was used in the calculations. Figure 7 presents the energy reaction profiles for initiation and propagation stages. These profiles were in good agreement with experimental data, but did not reflect side reactions experimentally observed, in particular the formation of macrocycles. This aspect of organocatalyzed ROP is discussed in Section 4, Section 5, Section 6 and Section 7.

The use of DFT modeling for estimation of the reaction ability of different *N*-heterocycles (4-pyrrolidino-pyridine, imidazole, 2-methylpyridine, pyridine, and pyrrole) in ROP of lactide on the basis of calculated proton affinities is also of note [55]. We suspect that such appreciation does not sufficiently predict the relative catalytic activity of basic organocatalysts in ROP.

## 4. *N*-Heterocyclic carbenes (NHC) and Related Compounds

**Figure 8:**
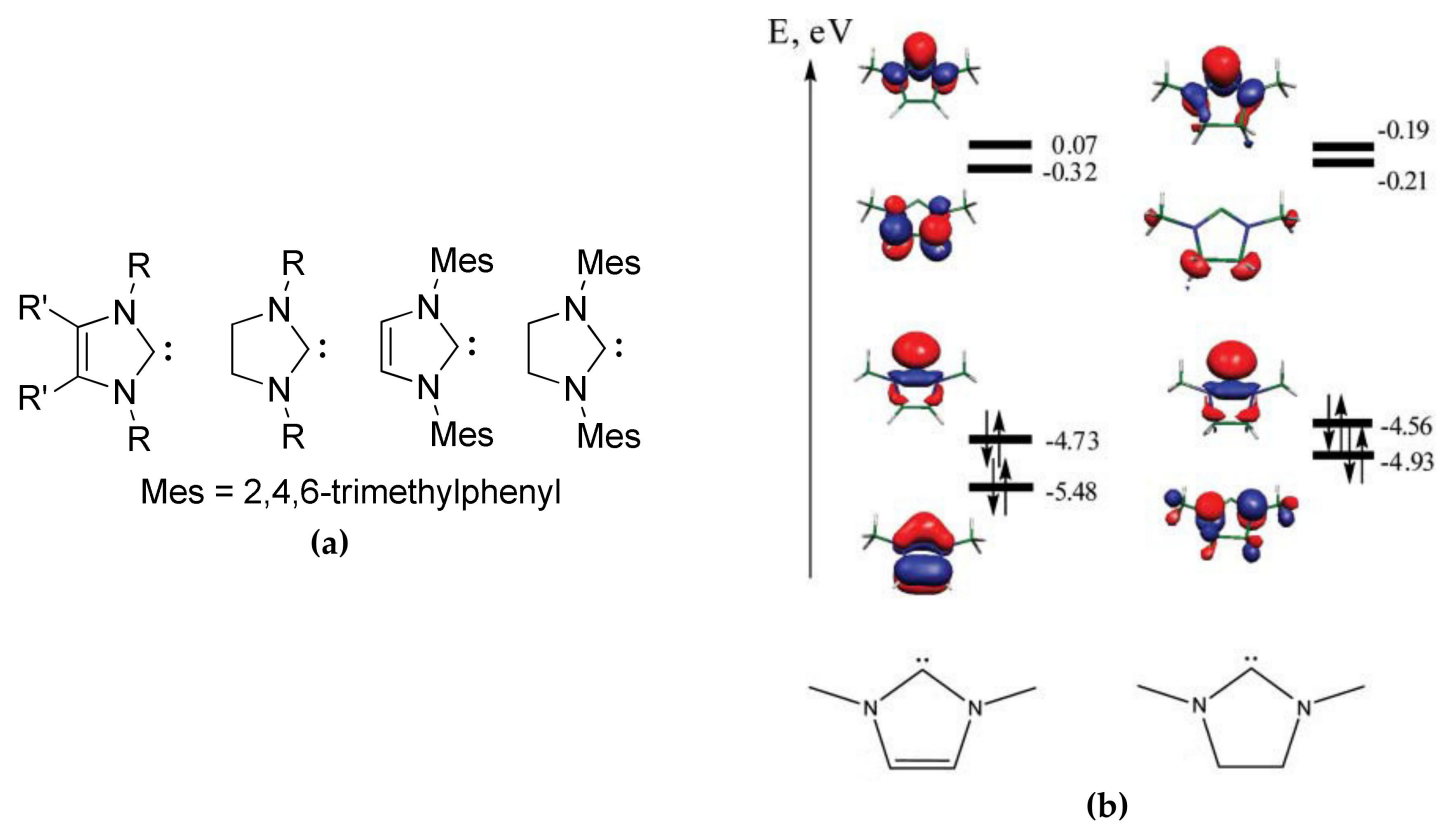
(**a**) The common structures of imidazole- and imidazoline-based N-heterocyclic carbenes (NHCs) and mesityl-substituted carbenes initially used in ROP of cyclic esters [63,64]; (**b**) The view and energies (eV) of two highest occupied molecular orbitals (HOMO) and two lowest occupied molecular orbitals (LUMO) of imidazole- and imidazoline-derived carbenes calculated while using Natural Bond Orbital (NBO) program. Reprinted with permission from [59]. Copyright (2009) Royal Society of Chemistry.

*N*-heterocyclic carbenes (NHCs, Figure 8a) represent the whole group of effective ROP catalysts; the mechanisms of their action and prospects of applications in polymer synthesis have been comprehensively reviewed [26,28,56]. NHCs are cyclic carbene species with two neighboring nitrogen atoms. A characteristic property of NHC is their high coordinating ability that their bulkiness and strong electron donation cause (Figure 8b); the results of DFT calculations of NHCs electronic structure [57,58,59] allow for explaining the chemical behaviour of NHCs that are strong Brønsted and Lewis bases [26,60,61]. The basicity of NHCs plays a crucial role in the catalytic activity of these compounds [62]. Most of the NHCs are moisture sensitive, hence they have to be handled under an inert atmosphere. The first report of the catalytic application of imidazole-based NHC (mesityl-substituted carbene was used, Figure 8a) in ROP of cyclic esters, such as *l*-LA, εCL, and β-butyrolactone (βBL) dates back to 2002 [63]. The reactions were carried out in the presence of alcohols, the ROP of *l*-LA that was catalyzed by NHC proved to be much faster than the DMAP-catalyzed reaction. After three years, imidazoline-derived NHC (Figure 8a, right) was successfully used in ROP of LA [64]. Linear polymers and macrocyclic oligomers of different cyclic esters were both obtained in the following years while using NHCs catalysts [23,26,28,56,65,66].

**Figure 9:**
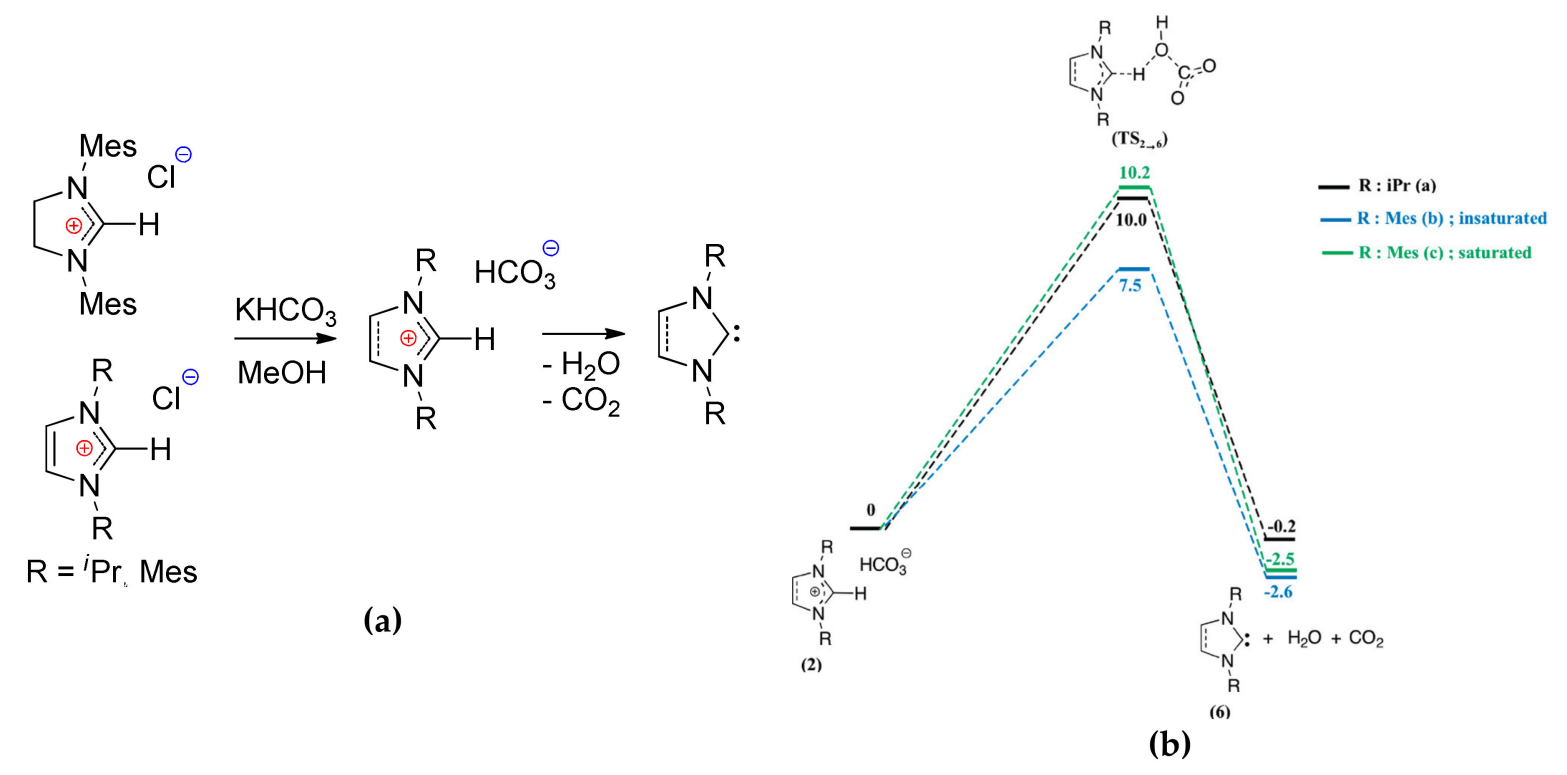
(**a**) Generation of NHCs from the corresponding hydrogen carbonates; (**b**) Energy profile calculated for decomposition of NHC hydrogen carbonates (free energies at 25 °C including zero point energy (ZPE) corrections, kcal/mol). Reprinted with permission from [71]. Copyright (2012) American Chemical Society.

The methods of the synthesis and in situ generation of NHC have been discussed in a number of publications and reviews for example, [26,67,68,69,70]. Taton et al. [71] proposed an efficient method of the generation of imidazole- and imidazoline-based NHCs that were suitable for organocatalytic ROP. This method was based on the NHCs hydrogen carbonates that were generated by the reaction of easily accessible imidazolium chlorides with KHCO_3_ (Figure 9a). The obtained hydrogen carbonates were used as the initiators of LA polymerization. The authors performed DFT modeling of the decomposition of these precursors with a formation of NHCs at B3LYP/6-31G** [39,40,51] level. Figure 9b presents the reaction profiles of these reactions. Relative low activation barrier values allowed for explaining the efficiency of hydrogen carbonate pre-catalysts. Note that NHCs are able to chemical bonding with CO_2_, and this process is exergonic. In [71] it was also found that this reaction is reversible; and, the barrier of decomposition of NHC–CO_2_ adducts were estimated by the values in the range 9.9–10.7 kcal/mol. The formation of the water in the course of pre-catalyst decomposition was the only factor limiting the use of this method.

**Figure 10:**
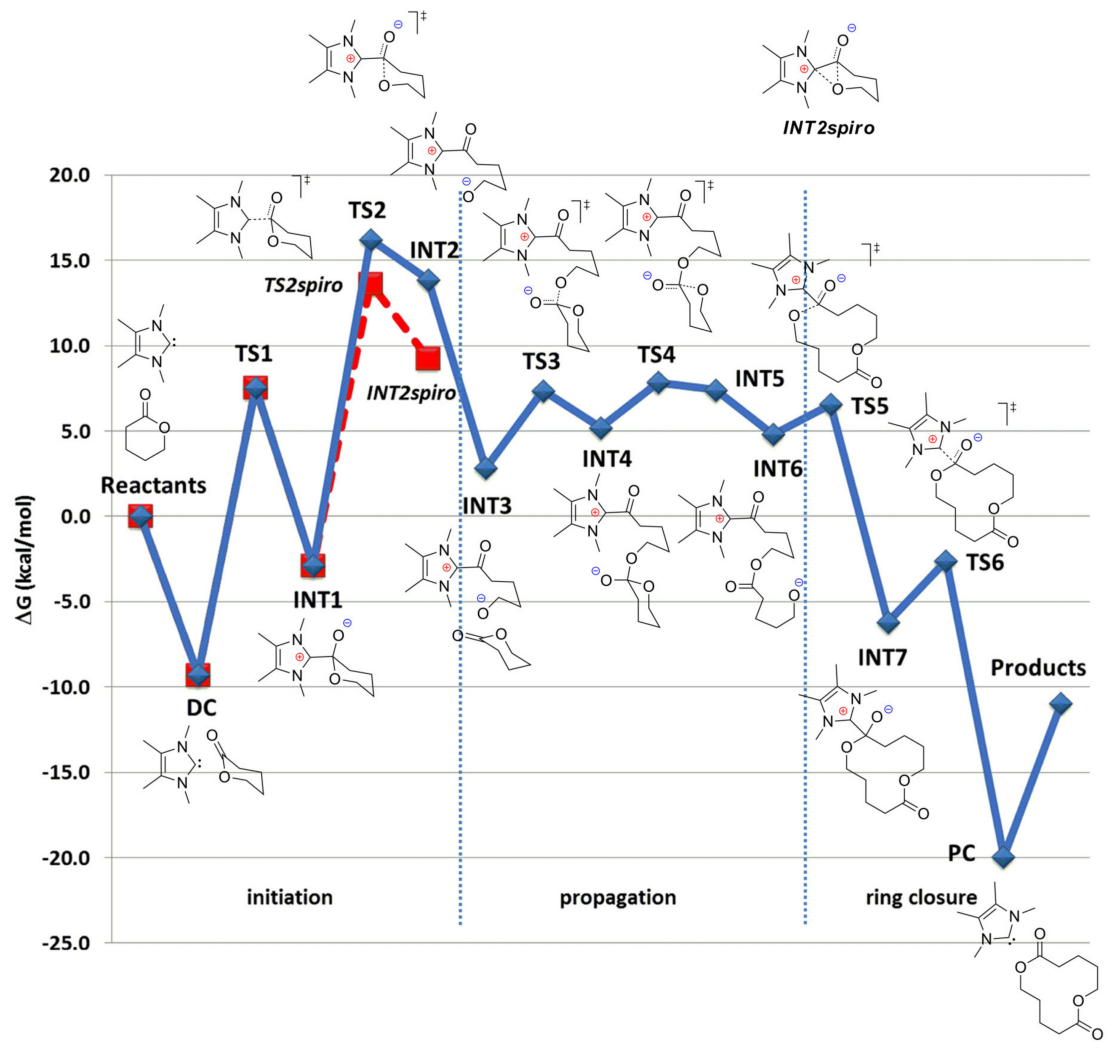
Reaction energy profile (kcal/mol) for the catalytic dimerization of δVL with NHC. All energies relative to isolated reactants (2 δVL + NHC). Reprinted with permission from [75]. Copyright (2014) American Chemical Society.

The hydrogen-bonding mechanism is preferable in base-catalyzed ROP if ROH initiator presents in the reaction mixtures, as was demonstrated for DMAP-catalyzed ROP (see Section 3). However, the high nucleophilicity and basicity of NHCs allows for the zwitterionic mechanisms to be competitive under the mild reaction conditions. Without ROH initiator, NHCs may act as strong nucleophiles that form low-energy adducts with cyclic esters; these adducts can undergo ring-opening without the assistance of ROH initiator. Such a reaction pathway can lead to the formation of macrocyclic polyesters [72,73] and poly(ethylene phosphate)s [74] that exhibit intriguing properties. The formation of these polymers from δVL was the subject of the investigation of Waymouth et al. [75], who included thorough DFT modeling of the reaction catalyzed by tetramethyl-substituted NHC at B3LYP/6-311+G(2d,p) [39,40,76,77,78] level of theory. The calculations of the reaction pathway leading to the formation of cyclic δVL dimer found that the highest activation barrier in the whole process was located at the ring-opening of the tetrahedral intermediate INT1 that resulted from nucleophilic attack of the NHC on the δVL carbonyl moiety (Figure 10). Non-aromatic intermediate INT2*spiro* (Figure 10) has proven to be a dead end stationary point, the only relaxation to INT1 was found for this molecule. Note that useful information regarding charge distribution in transition states presented in Figure 10 was also provided in [75].

The first ring-opening transition state TS2 was rate-limiting; therefore, the whole process would be characterized by slow initiation, rapid propagation, and facile cyclization to liberate macrocyclic products. The insertions of next δVL molecules were estimated as energetically favorable, the effect of stabilization of curled conformation by Coulombic attraction in INT2-like intermediates (INT5,6 etc.) lowered in line with the increase of the number of δVL molecules enchained.

**Figure 11:**
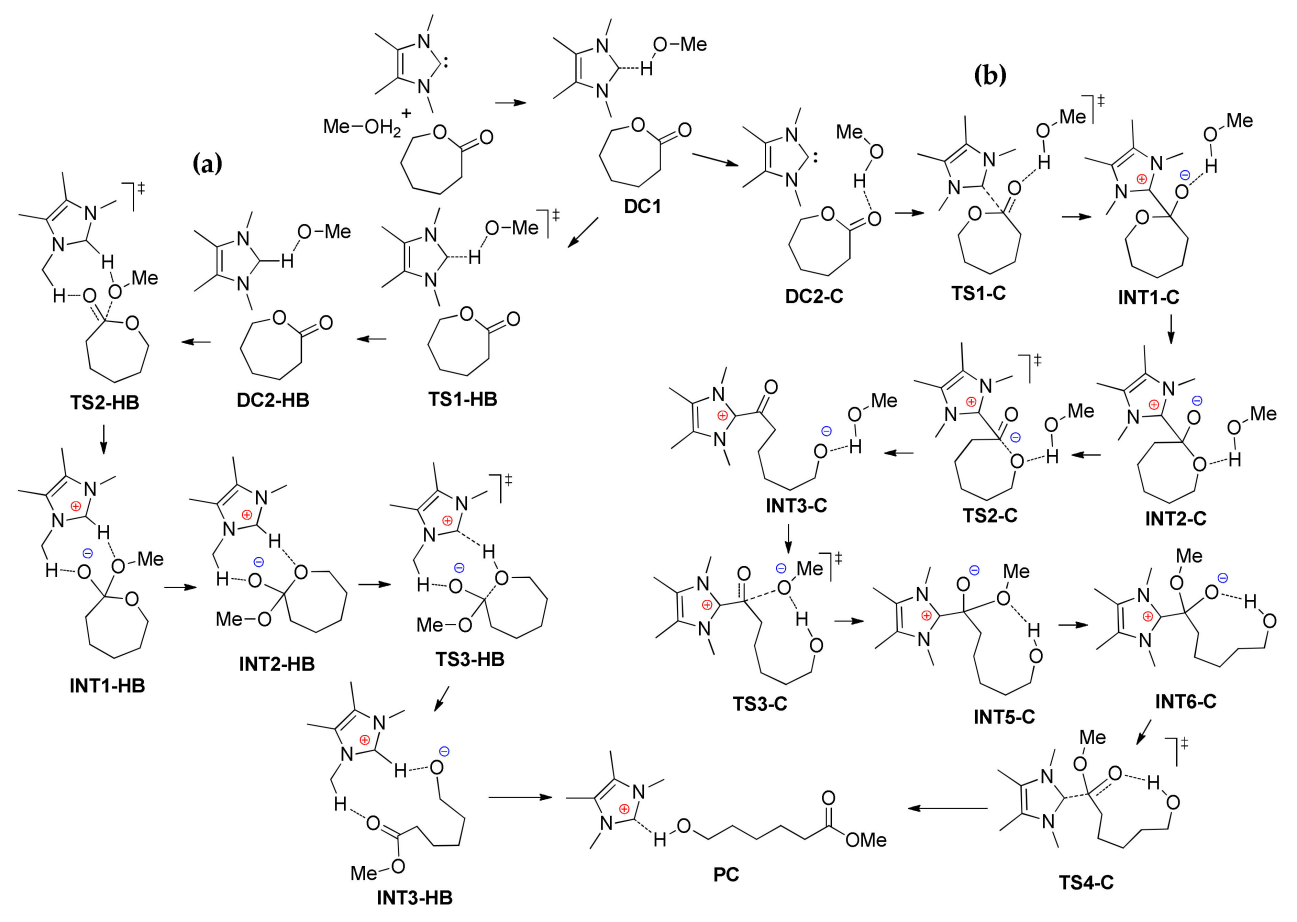
Computed stationary points and transition states for MeOH-assisted hydrogen-bonding (**a**) and nucleophilic (**b**) mechanisms of NHC-catalyzed εCL ROP [79].

**Figure 12:**
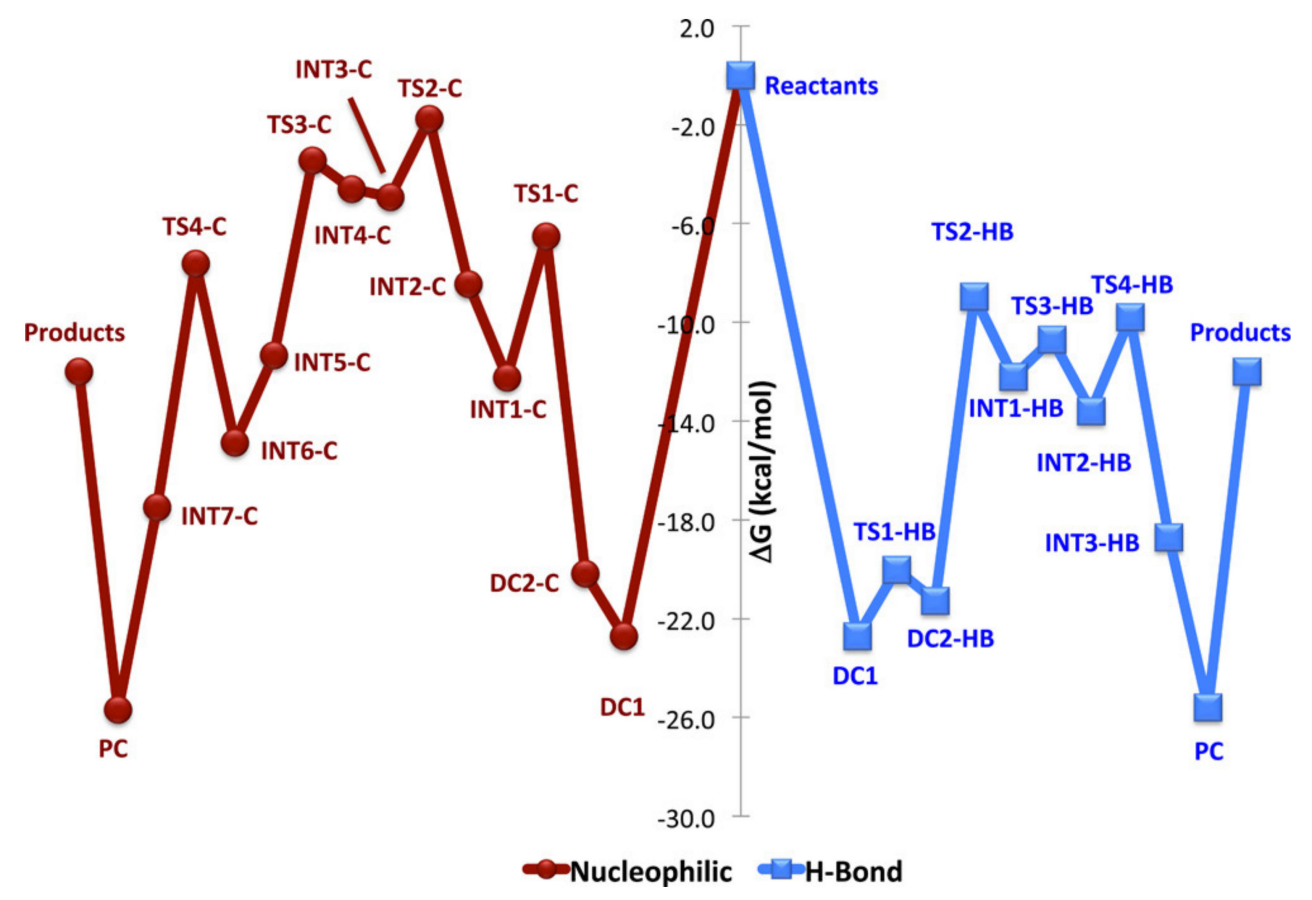
Energy reaction profiles of NHC-catalyzed εCL ROP in the presence of MeOH for nucleophilic (**red**) and hydrogen-bonding (**blue**) mechanisms. Reprinted with permission from [79]. Copyright (2015) American Chemical Society.

Waymouth et al. performed computational investigations of εCL ROP catalyzed by NHCs in the presence and in the absence of MeOH initiator [79] at B3LYP/6-311+G(2d,p) [39,40,76,77,78] level of theory. A continuum dielectric with the IEF-cPCM [80,81,82] method was utilized to represent reaction conditions (THF, ε = 7.58 at 298 K). The results of the modeling of εCL ROP in the frameworks of MeOH-free zwitterionic reaction mechanism were similar to those presented in Figure 11 for δVL ROP, and the activation energy was estimated by the value of 28 kcal/mol. The results of the modeling of MeOH-assisted εCL ROP were of greater interest; Figure 11 presents two plausible mechanisms of this reaction, and Figure 12 shows the free energy reaction profiles.

The reactant complex, DC1, was common to both the hydrogen-bonding and nucleophilic pathways. Ring-opening was the rate-limiting step for nucleophilic mechanism (TS2-C, Figure 11 and Figure 12), while the insertion of MeOH (TS2-HB) was characterized by the highest activation barrier for nucleophilic mechanism. Calculations predicted that the hydrogen-bonding mechanism is preferred to the mechanism that involves basic nucleophilic attack by about 6 kcal/mol. The results of calculations were compared with the experimental data; the acceleration of εCL ROP rate by more than an order of magnitude in the presence of MeOH was in good agreement with the DFT modeling results.

Cavallo et al. reported the results of the DFT optimization of GL and δVL adducts with different NHCs [83]. The authors did not discuss the ROP mechanisms for these cyclic esters; therefore, this article was inconsistent with the topic of our review.

**Figure 13:**
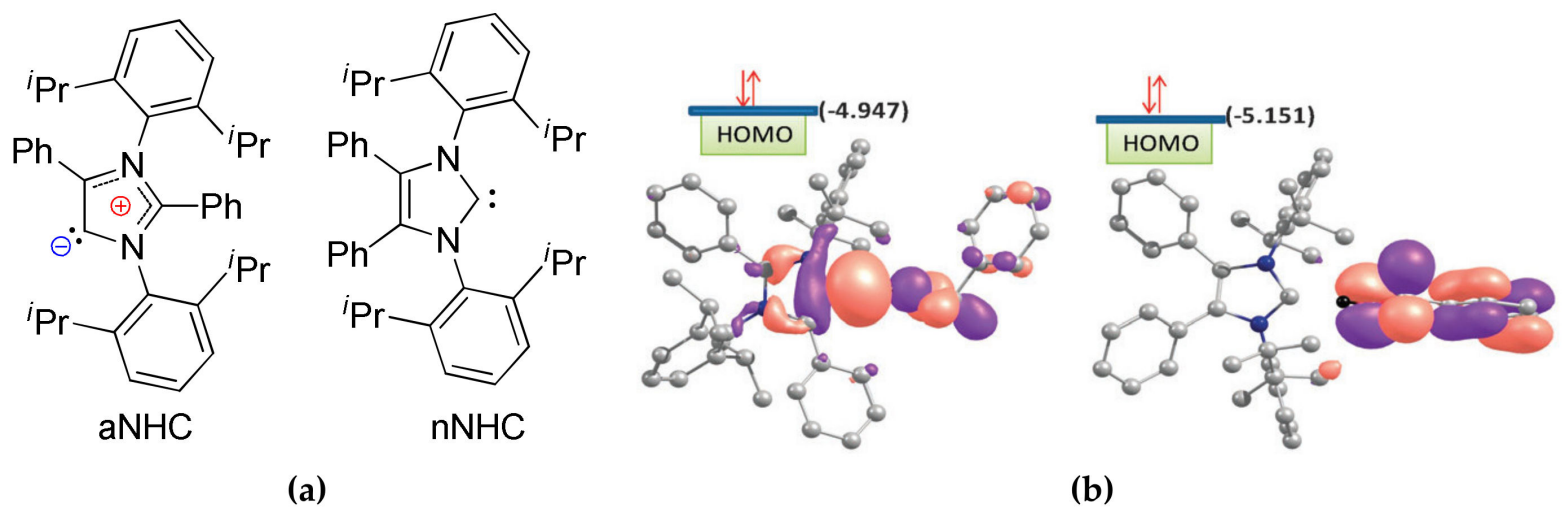
(**a**) The abnormal (aNHC) and normal (nNHC) carbenes; and, (**b**) HOMO of aNHC and bNHC adducts with BnOH (A and B, respectively). Reprinted with permission from [86]. Copyright (2011) Royal Society of Chemistry.

Mandal et al. used abnormal NHCs (aNHCs) [84,85], in which the carbene center is no longer located between the two nitrogen atoms, but is generated between nitrogen and carbon atoms (Figure 13a), in ROP of δVL, εCL and *d,l*-LA [86]. They found that aNHCs outperformed conventional NHCs by the catalytic activity in εCL ROP. The comparative DFT modeling of the structures of aNHC and its “normal” analog nNHC (Figure 13a) was performed at BP86/SVP [87,88] level of theory to clarify these experimental observations. When the HOMO of the hydrogen bonded aNHC–BnOH adduct (**A**, Figure 13b) was compared with that of the nNHC–BnOH (**B**, Figure 13b), it was observed that the HOMO of adduct **A** still preserves the σ lone-pair type character that supports that the reaction proceeds with a monomer activated pathway through nucleophilic attack of the carbene center of the adduct on the cyclic ester monomer, as depicted in Scheme 2. Additionally, this observation excludes the possibility of an initiator or activated alcohol mechanism for ROP. However, the HOMO of the adduct **B** was predominantly BnOH based and, hence, the σ-donating character of the carbene was no longer expected (Figure 13b). Thus, the hydrogen bonded adduct **B** would be expected to initiate ring opening polymerization through attack by the activated alcohol mechanism pathway, as Lohmeijer et al. reported [89] and discussed above. The HOMO of adduct **B** was more stable (−5.151 eV) than the HOMO of the adduct **A** (−4.947 eV) while taking that the HOMO of aNHC was higher in energy (−4.398 eV) than nNHC isomer (−4.991 eV), when BnOH is present, into account. This indicated that the adduct **A** was predominantly more basic than **B**. Furthermore, the NPA charge on the carbene carbon of the adduct was substantially less (−0.117 e) in **A** as compared to that in **B** (+0.108 e), which indicated a better nucleophilic character at the carbene carbon for abnormal NHC.

## 5. TBD, Substituted Guanidines and Related Compounds

**Figure 14:**
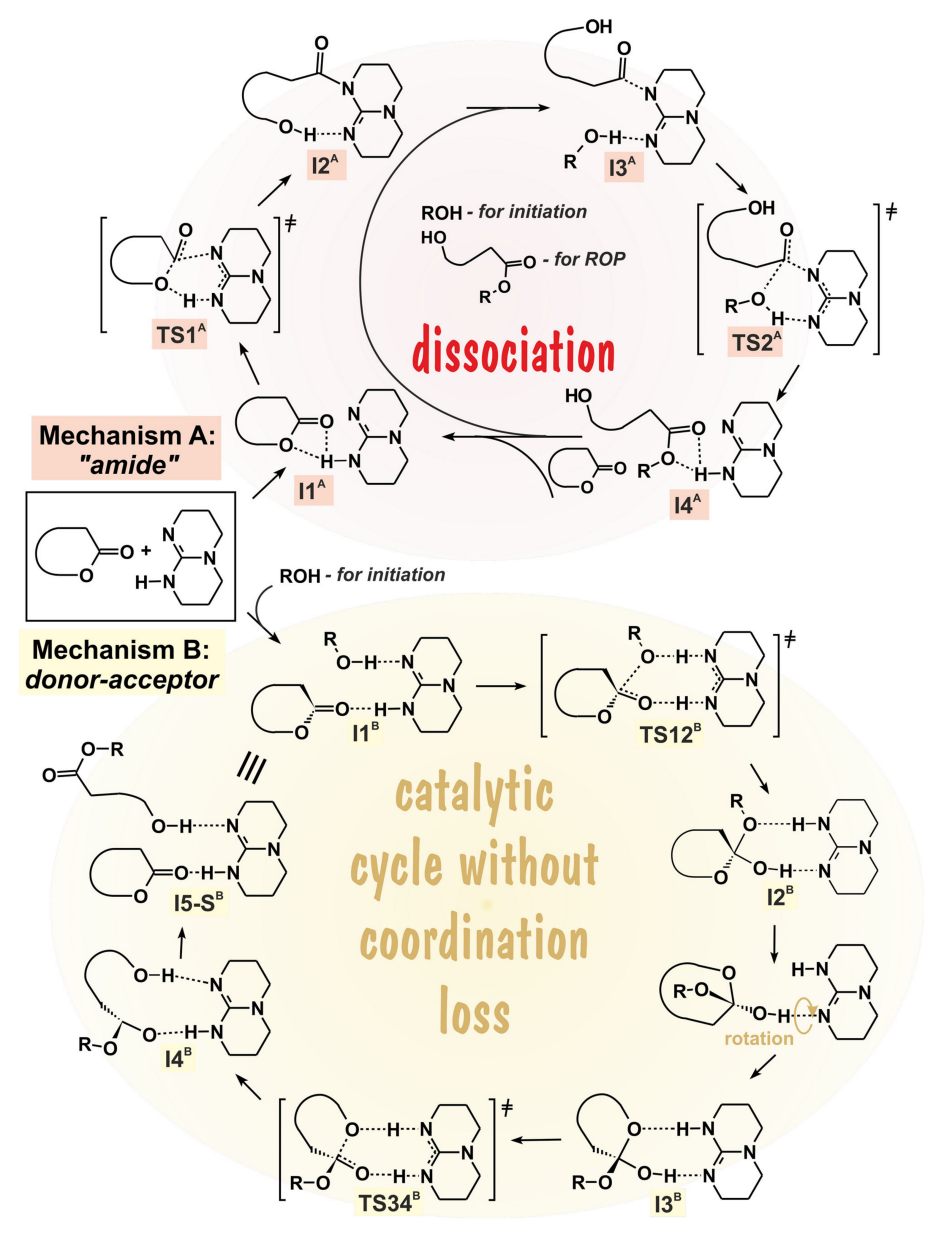
Alternative mechanisms of 1,5,7-triazabicyclo-[4.4.0]dec-5-ene (TBD)-catalyzed alcoholysis (ROP) of cyclic esters. The colored version of the figure presented in [94].

**Figure 15:**
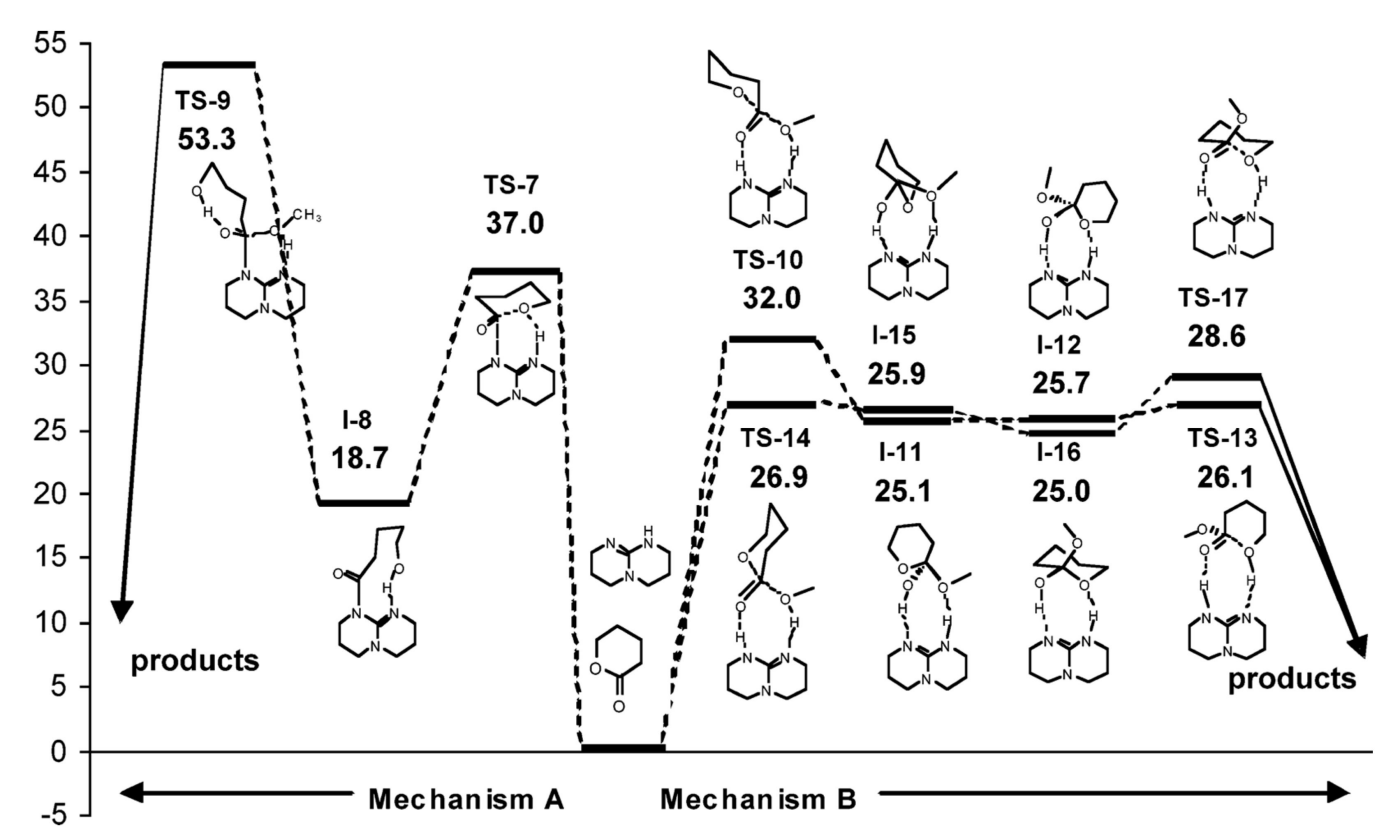
Energy profile and structures obtained for the reaction between δVL and MeOH. Reprinted with permission from [91]. Copyright (2007) American Chemical Society.

In 2006, Hendrick, Waymouth et al. studied the catalytic behaviour of 1,5,7-triazabicyclo-[4.4.0]dec-5-ene (TBD) in the ROP of δVL, εCL, and *l*-LA in the presence of 4-pyrenebutanol initiator [90]. For these reactions, the dual activation mechanism (also called as “amide” mechanism, Figure 14, mechanism A) was proposed. Simón and Goodman investigated the possibility of an alternative “donor-acceptor” mechanism (Figure 14, mechanism B) in comparison with amide mechanism in 2007 [91]. While using DFT modeling at the B3LYP/6-31+G* [40,78,92,93] level of theory, they drew the energy profiles for two alternative reaction mechanisms A and B of TBD-catalyzed δVL polymerization in the presence of MeOH (Figure 15). This reaction profiles clearly demonstrated that the donor-acceptor mechanism of TBD-catalyzed ROP is preferable for moderately strained lactones, such as δVL.

**Figure 16:**
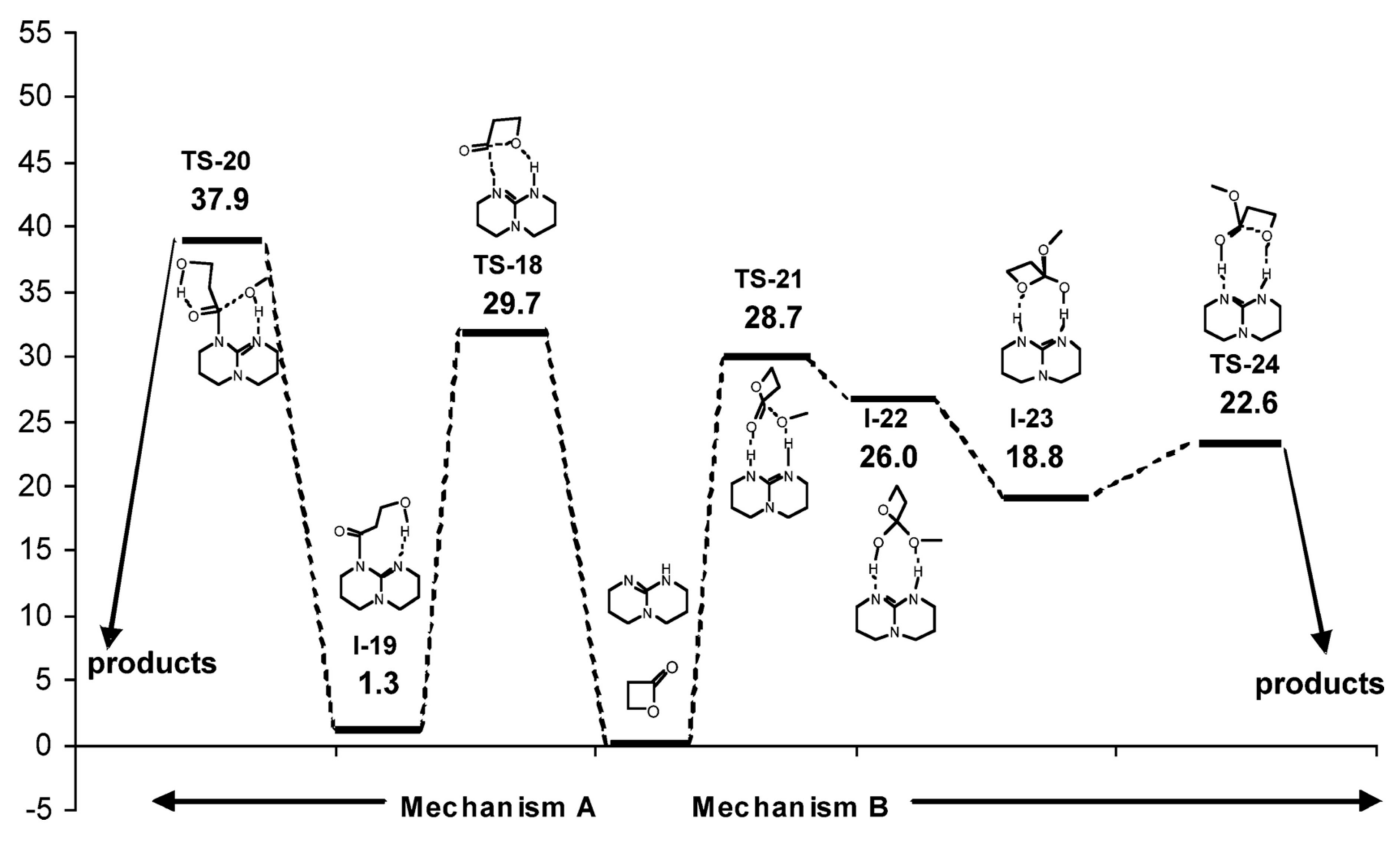
Energy profile and structures obtained for the reaction between β-propiolactone and MeOH. Reprinted with permission from [91]. Copyright (2007) American Chemical Society.

In contrast to δVL, β-butyrolactone (βBL) was inactive in TBD-catalyzed ROP at 25 °C. This difference was explained in [91] by the results of the calculations of TBD-catalyzed β-propiolactone methanolysis (Figure 16). Unlike δVL methanolysis, in the case of four-membered lactone, Gibbs free energy barriers for nucleophilic addition of TBD (Mechanism A) and methanol acid-base addition (Mechanism B) were similar. The formation of the stable *N*-acyl adduct (I-19, Figure 16) was highly probable, but the following TS-20 (ΔG = 36.6 kcal/mol) looked unattainable under the mild reaction conditions. However, at elevated temperatures, TBD was able to catalyze polymerization of βBL [95]. The formation of TBD end-capped polymers confirmed the amide mechanism A, which was updated in this work by the assumption of zwitterionic intermediate formation from I-19, and by the detection of the products of I-19 dehydration as the C(O)CH=CHMe end-groups.

**Figure 17:**
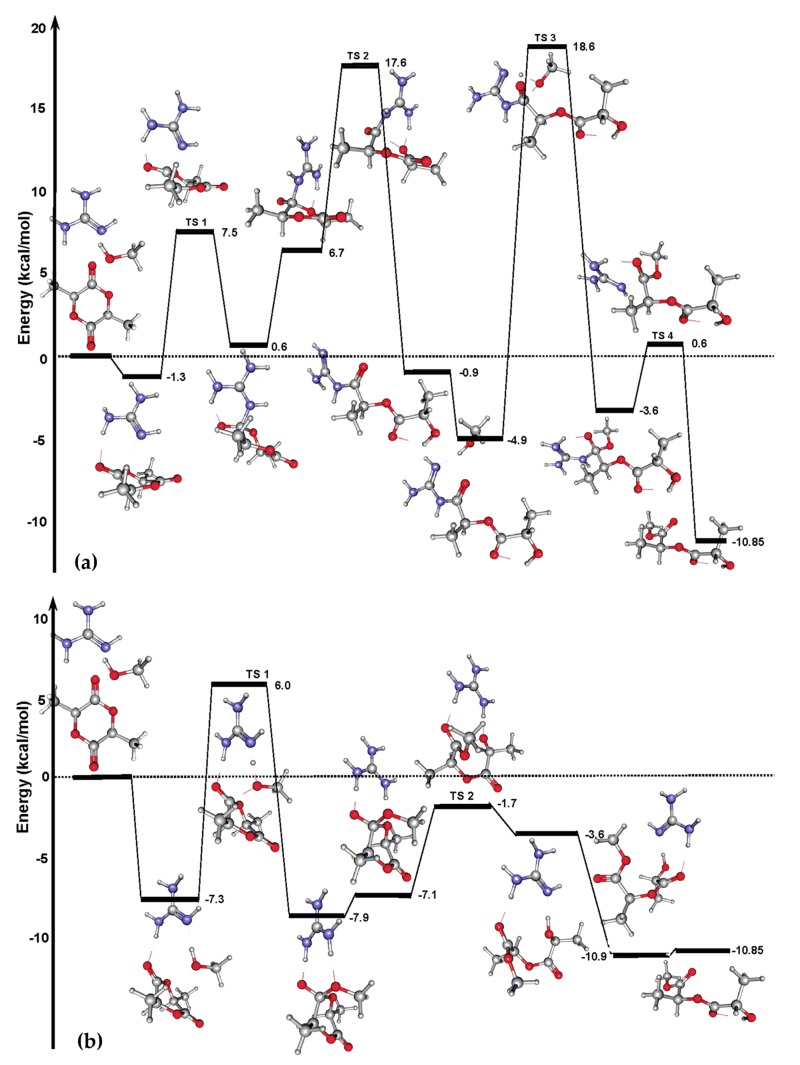
Energy profiles (electronic energies) and structures obtained by DFT modeling of guanidine-catalyzed methanolysis of *l*-LA for (**a**) amide mechanism and (**b**) hydrogen-bonded mechanism. Reprinted with permission from [96]. Copyright (2008) American Chemical Society.

In parallel with the investigations of TBD-catalyzed ROP of lactones that Simón and Goodman performed [91]; Rice et al. studied the mechanism of TBD-catalyzed polymerization of lactide [96]. They used guanidine molecule as a truncated model of the TBD catalyst, and calculated the energy profiles for two possible reaction pathways (Figure 17) at the MPW1K/6-31+G [41,92,97,98] level of theory to compare alternative reaction mechanisms; the effect of the solvent (CH_2_Cl_2_) was taken into account by the c-PCM [81,82] method. For amide mechanism A, the activation barrier of the first step (formation of amide complex) was very low (8.8 kcal/mol), but the barriers of the subsequent steps were much more higher up to 23.5 kcal/mol (Figure 17a). The energy barriers for hydrogen-bonded mechanism B were found to be substantially lower (Figure 17b); the rate-limiting step was guanidine-assisted MeOH insertion on the carbonyl group. The value of the activation barrier (13.3 kcal/mol for guanidine) was considerably reduced for TBD: the relative free energy of this transition state was only 7.9 kcal/mol. These results predict the hydrogen-bonding mechanism to be preferable in TBD-catalyzed LA ROP. It should be noted that the zwitterionic ROP mechanism might well be feasible in the absence of ROH initiator: thus, the formation of TBD-bound PLA was experimentally detected when *l*-LA polymerized without the addition of alcohol [99].

**Figure 18:**
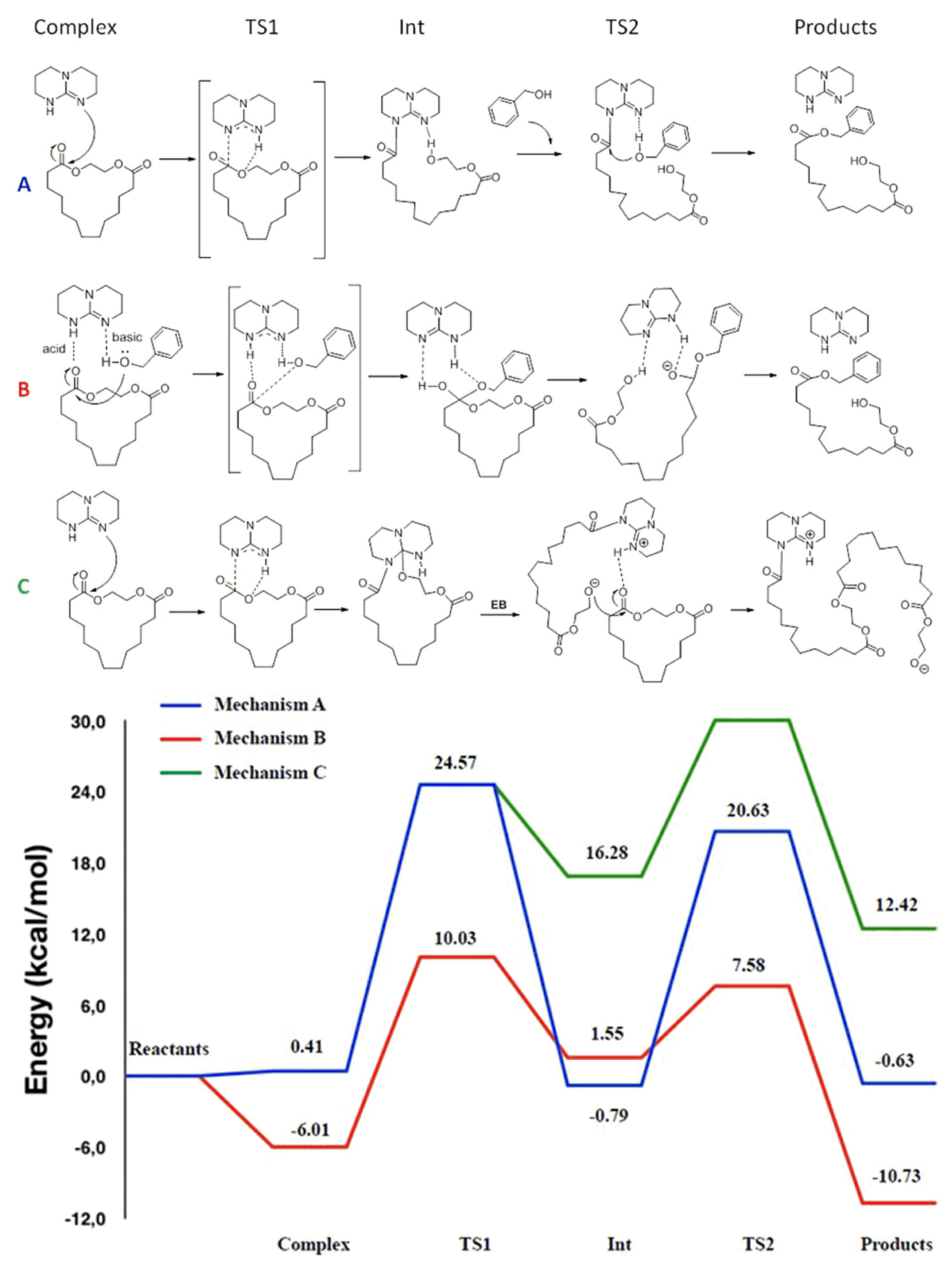
Activation mechanisms of TBD catalyst for the ROP of ethylene brassylate (top) and free energy profiles obtained by DFT calculations of different possible activation mechanisms. Reprinted with permission from [100]. Copyright (2014) Wiley-VCH Verlag GmbH & Co.

Mecerreyes et al. performed comparative DFT modeling of the initiation stage of TBD-catalyzed polymerization of ethylene brassylate macrolactone [100] at the M06-2X/6-31+G(d) [51,54] level. The comparison of the energy reaction profiles (Figure 18) allowed for drawing a conclusion about the preference of the hydrogen-bonded mechanism B. Thus, TBD, in combination with the ROH initiator, catalyzed the ROP of moderately strained lactones and lactide acting as an acid and base simultaneously, activating both cyclic ester and ROH via the formation of two hydrogen bonds.

**Figure 19:**
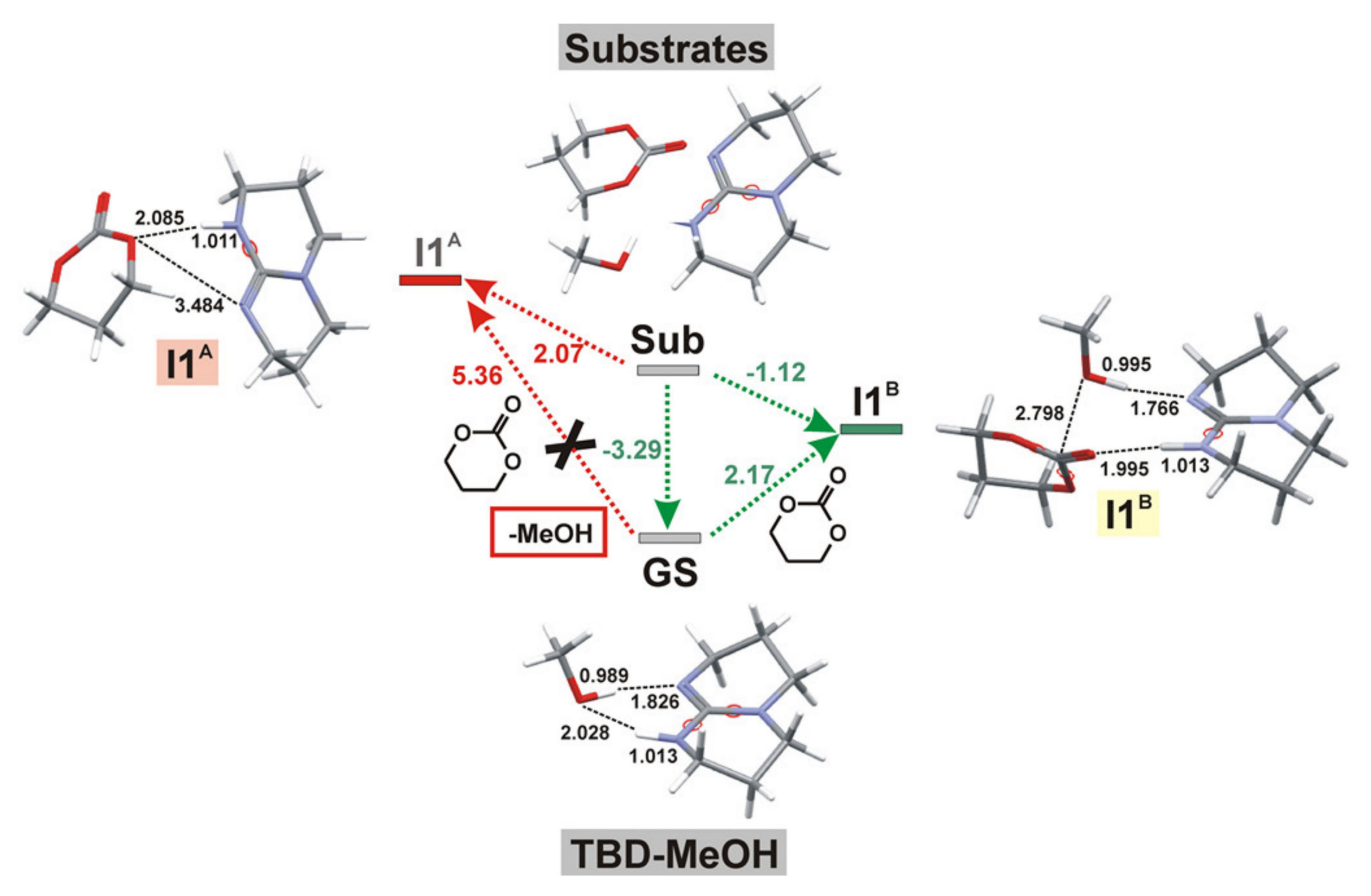
The formation of the complex TBD-MeOH as a key criterion for the choice of the reaction mechanism. The changes of free energies are given in kcal/mol. The colored version of the figure presented in [94]. Copyright (2015) Springer Nature.

**Figure 20:**
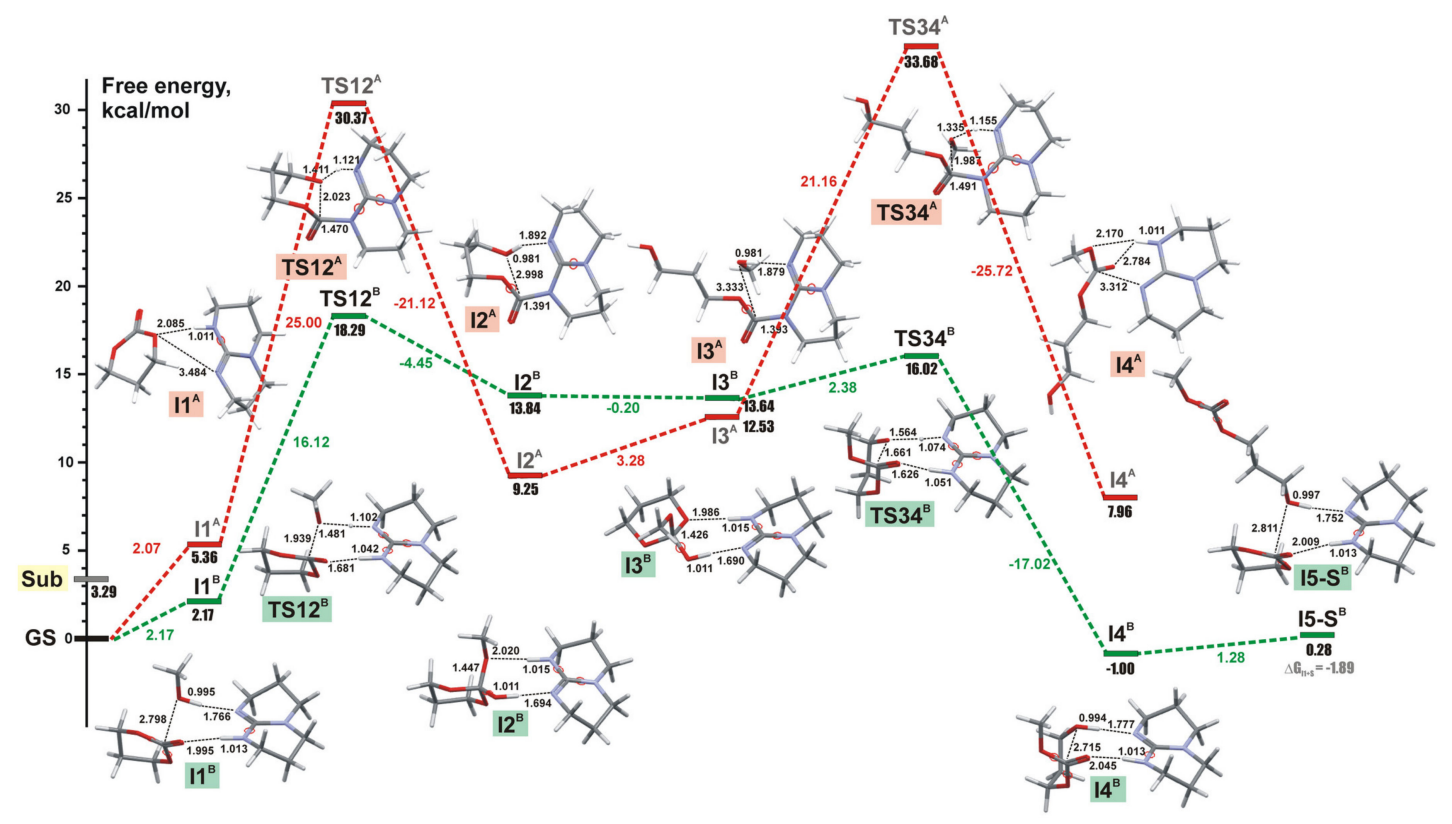
Energy profiles obtained by DFT calculations for TBD-catalyzed ring-opening methanolysis of trimethylene carbonate (TMC) for mechanisms A (**red**) and B (**green**). The colored version of the figure presented in [94]. Copyright (2015) Springer Nature.

Our scientific group performed a detailed study of TBD-catalyzed ROP of 5-, 6- and 7-membered cyclic esters and carbonates [94] at B3LYP/6-311G(d) [39,40,51,98] level of theory while using methanolysis as a model reaction. First, we calculated the geometries of the reaction complexes that formed by TBD, trimethylene carbonate (TMC), and MeOH and found that TBD-MeOH complex can be considered as a common ground state for all of the processes under study (Figure 19). The choice between alternative ROP mechanisms that are presented in Figure 14 was made by the comparison of the reaction profiles of TMC methanolysis (Figure 20).

**Figure 21:**
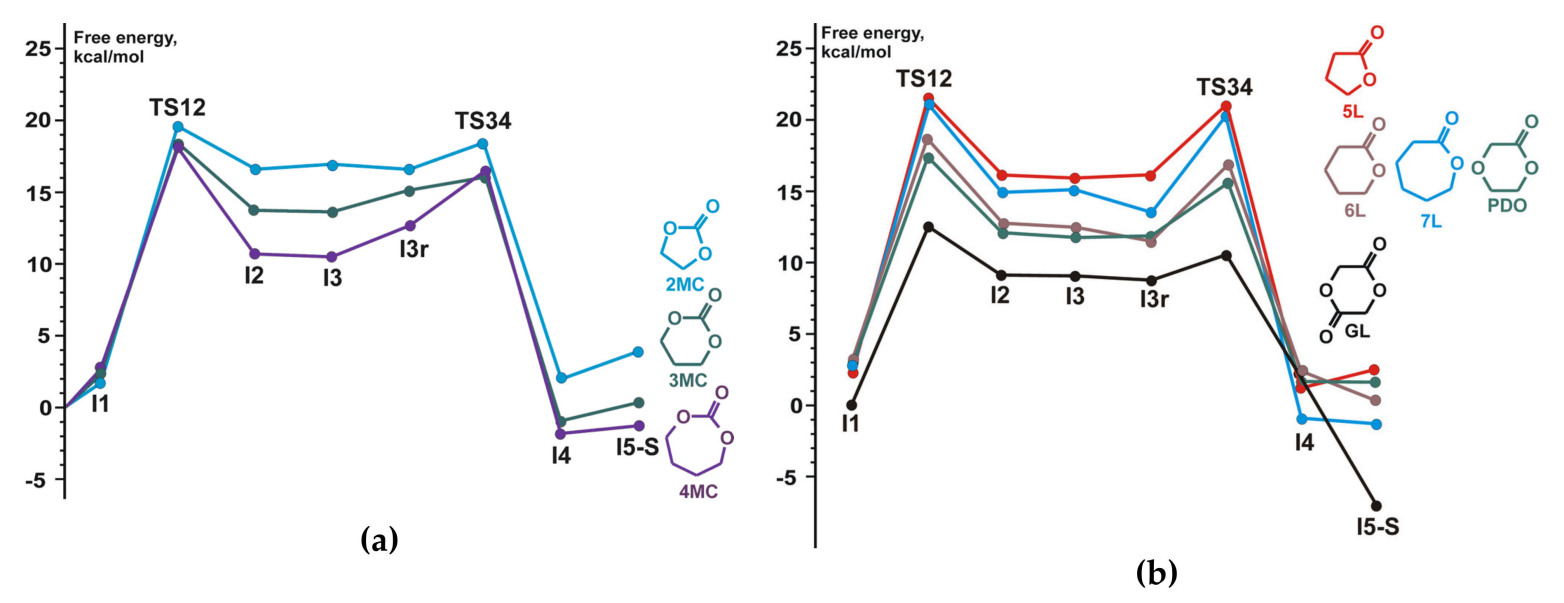
DFT modeling of TBD-catalyzed methanolysis of (**a**) 5-, 6-, and 7-membered cyclic carbonates and (**b**) γBL, δVL, εCL, polymerizability of 1,4-dioxan-2-one (PDO), and glycolide (GL). The colored version of the figure presented in [94]. Copyright (2015) Springer Nature.

**Figure 22:**
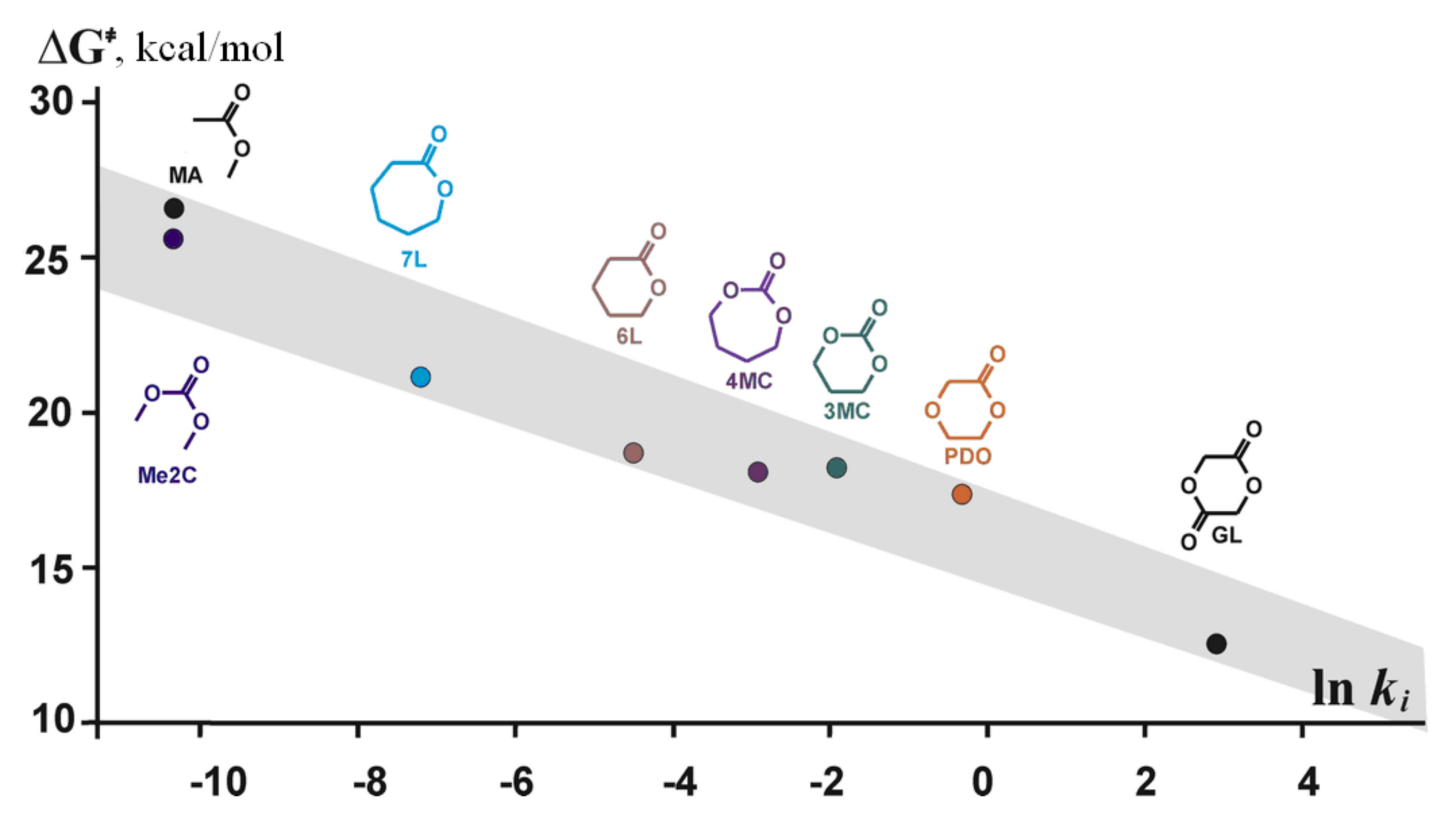
ΔG^≠^ – ln *k_i_* correlation in TBD-catalyzed ROP/transesterification. The colored version of the figure presented in [94]. Copyright (2015) Springer Nature.

The reaction pathway B was found to be preferable; high energy of the insertion transition state TS12^A^ was correlated with the experimental fact of the inertness of TMC relative to TBD in the absence of alcohol initiator under the ambient conditions. We analyzed methanolysis of 5-, 6-, and 7-membered cyclic carbonates, lactides γBL, δVL, εCL, PDO, and glycolide GL in the frameworks of the mechanism B; Figure 21 presents the calculated energy profiles. We also calculated the energy profiles for methyl acetate and dimethyl carbonate transesterification and found that the corresponding activation barriers much more higher than the barriers for methanolysis of cyclic esters. The results of calculations were compared with the results of polymerization and transesterification experiments, good correlation between calculated free activation barriers and polymerization/transesterification rates was demonstrated (Figure 22).

**Figure 23:**
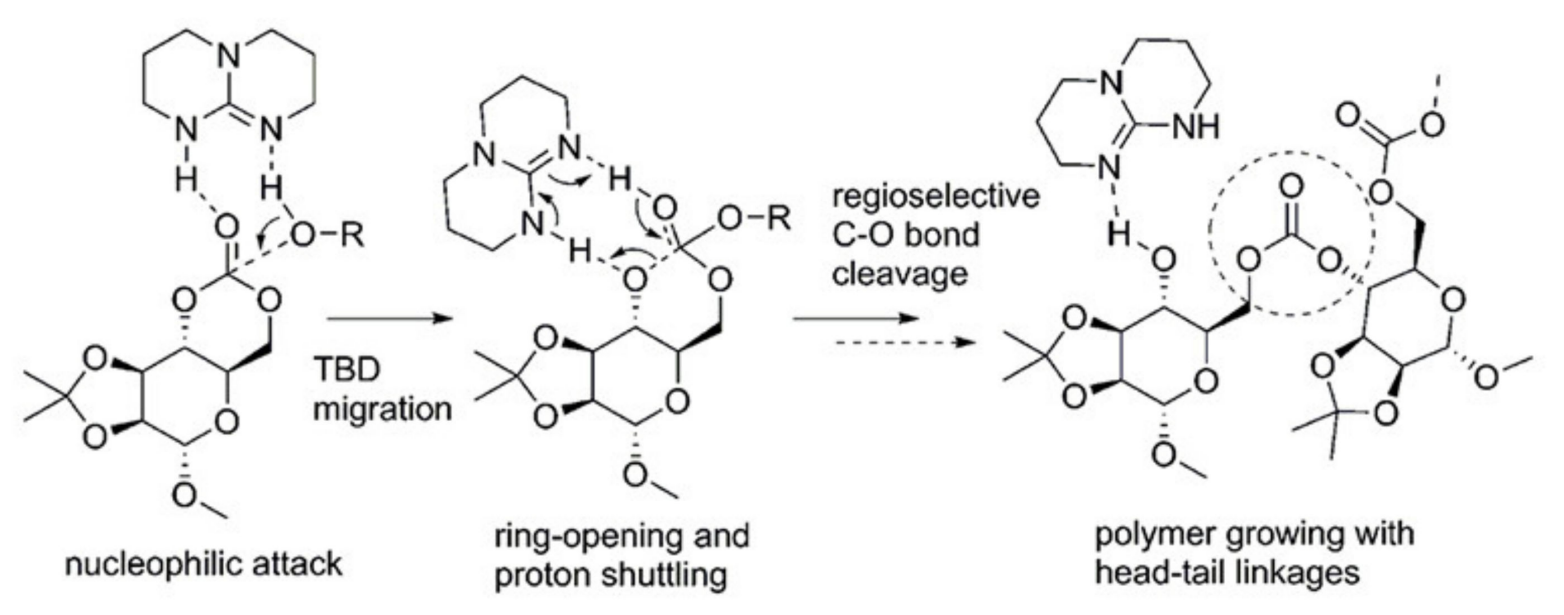
Formation of regioregular polycardonate in TBD-catalyzed ROP of *d*-mannose-derived cyclic carbonate [101].

Buchard et al. [101] studied TBD-catalyzed polymerization of *d*-mannose-derived cyclic carbonate experimentally and theoretically at rωB97XD/ 6-311++G(2d,p)/CPCM [82,98,102] level of theory. They analyzed the stereochemistry of the initiation stage, and demonstrated the preference of head-to-tail polymerization to explain the regioselectivity of polymerization (Figure 23).

**Figure 24:**
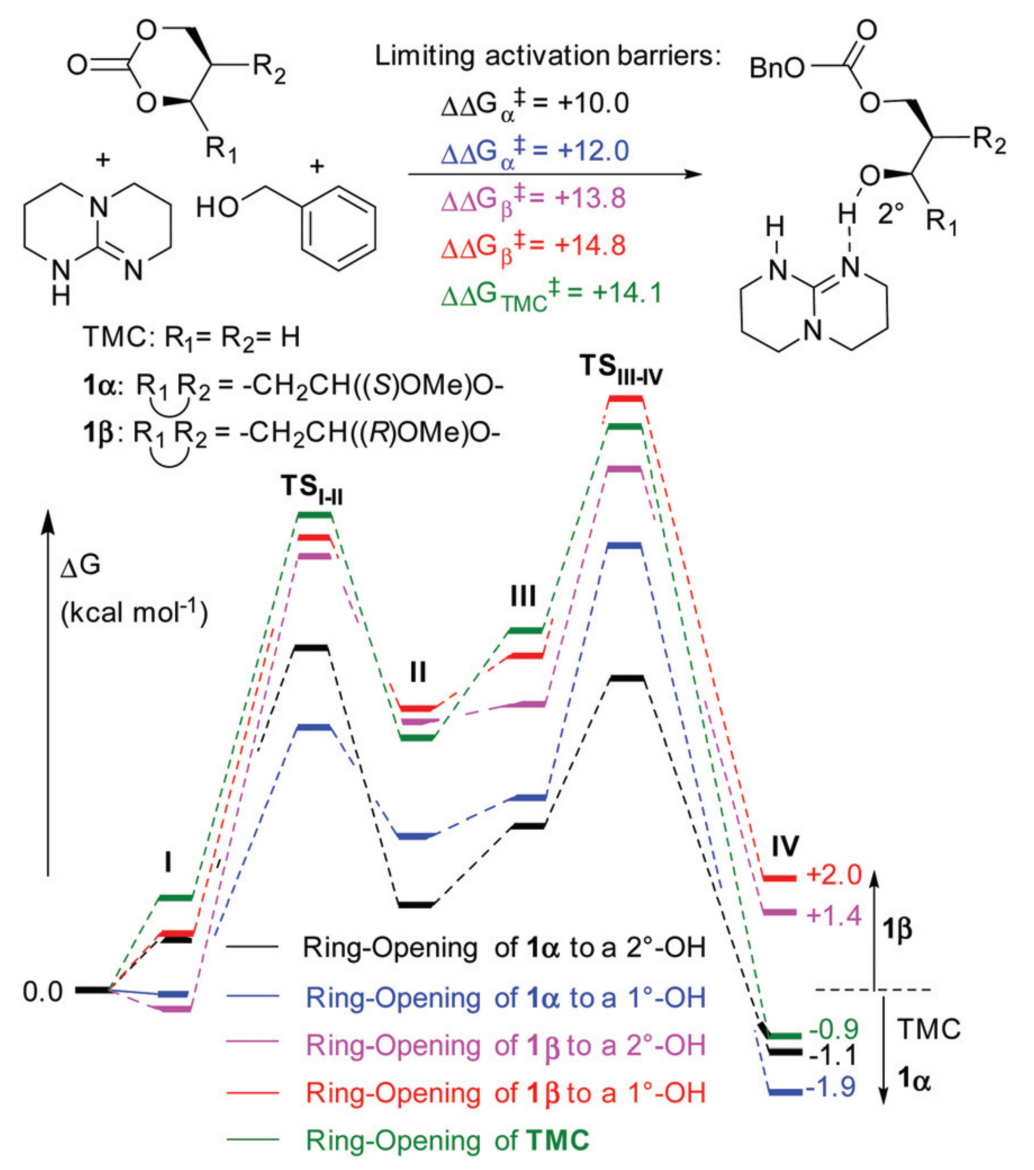
DFT modeling of the initiation step in the ROP of **1α**, **1β**, and TMC with TBD catalyst and BnOH initiator involving formation of ternary complex **I,** nucleophilic addition of BnOH (**TSI–II**) to form quaternary intermediate **II**, TBD migration **III** for ring-opening (**TSIII–IV**) to expose a primary (1°–OH) or secondary alcohol (2°–OH) in **IV**. Reprinted from [103]. Copyright (2017) Royal Society of Chemistry.

Buchard et al. performed the comparative DFT modeling of the initiation step of TBD-catalyzed ROP of TMC and 2-deoxy-*d*-ribose derived cyclic carbonates **1α** and **1β** (Figure 24) [103] at rωB97XD/ 6-311+G(2d,p)/CPCM [82,98,102] level of theory. By the results of calculations, TMC was found to be the most active monomer; however, TMC and **1α** incorporated in equal ratios in copolymerization experiments.

**Scheme 3:**
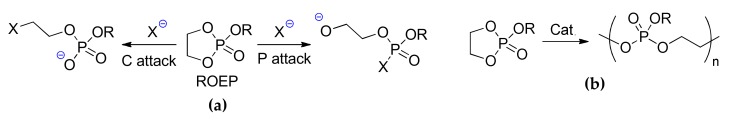
(**a**) The possible directions of the nucleophilic attack on cyclic ethylene phosphates [107]; and, (**b**) ROP of cyclic ethylene phosphates.

Cyclic ethylene phosphates (ROEP, Scheme 3) are prospective ROP substrates for the synthesis of hydrophilic and biodegradable poly(ethylene phosphate)s [6,7,104,105,106]. Two directions of the nucleophilic attack on ethylene phosphate molecule could be considered in a formal sense (Scheme 3a). Bendikov et al. [107] experimentally and theoretically studied the reactions of EtOEP with different nucleophiles at B3LYP/6-31+G* [40,78,92,93] level of theory, and found that C–O bond cleavage (C attack) was preferable for C, P and sterically hindered O-nucleophiles, while P–O bond cleavage (P attack) was preferable for OH^−^ and OMe^−^ nucleophiles. The latter direction was found to be preferable for MeOEP hydrolysis; ab initio calculations also confirmed this issue [108].

**Scheme 4:**
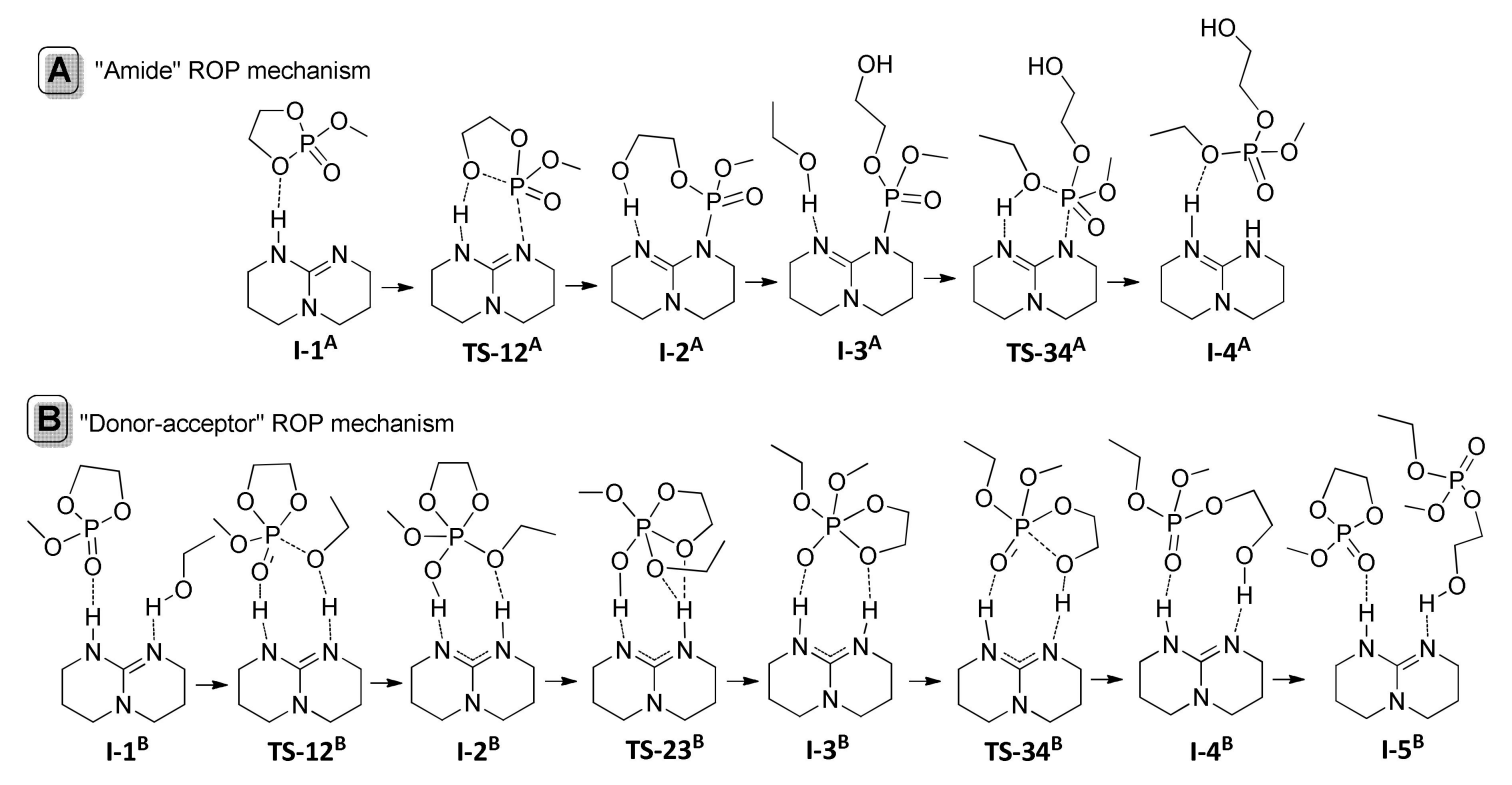
Amide (**A**) and donor-acceptor (**B**) mechanisms for the TBD-catalyzed ROP of MeOEP [109].

**Figure 25:**
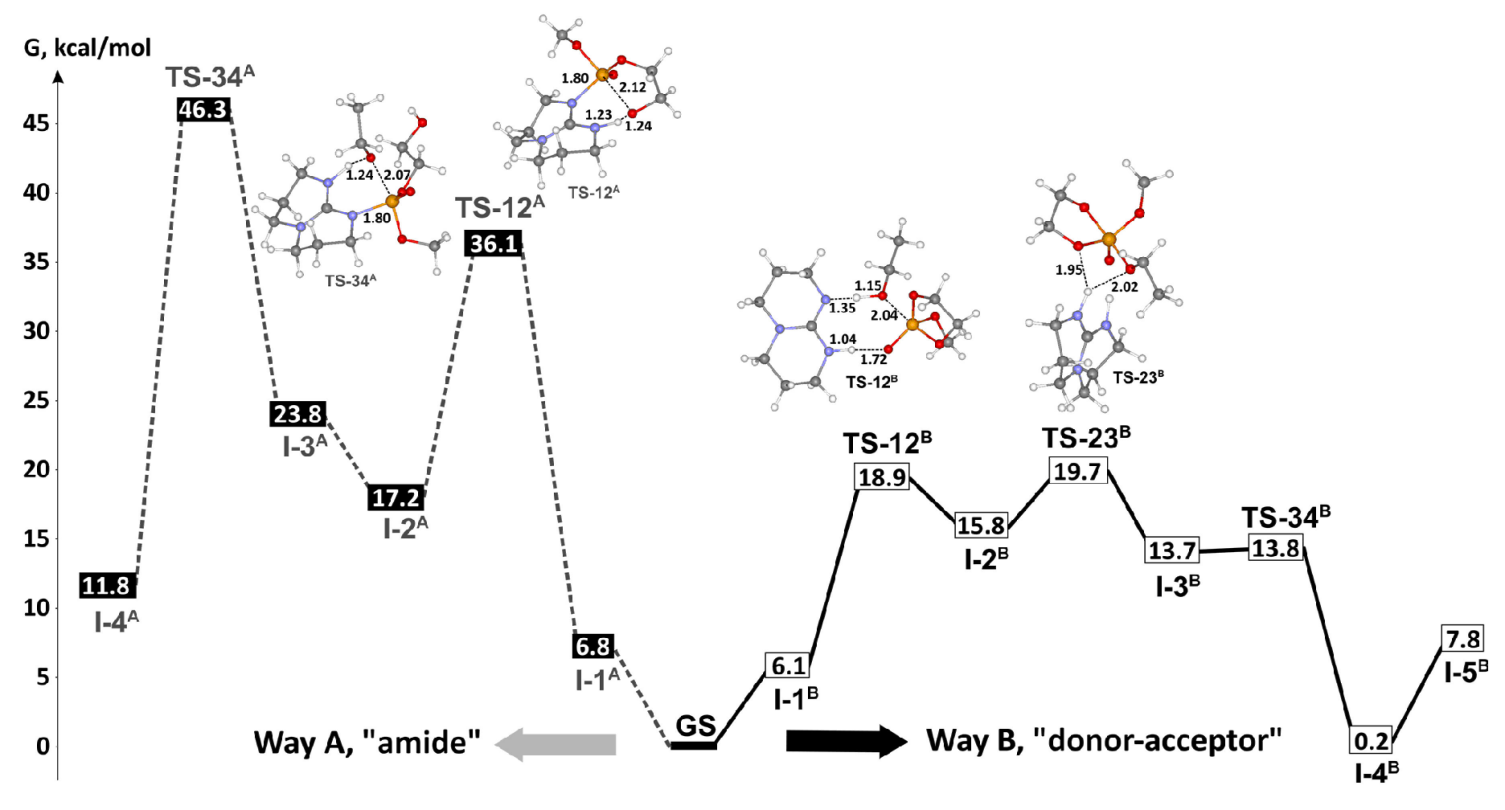
Energy profile obtained by DFT calculations of TBD-catalyzed ring-opening ethanolysis of MeOEP for mechanisms A (----- dark gray, left) and B (— black, right) and molecular structures of the key transition states (bond lengths and interatomic contacts are provided in Å). Reprinted with permission from [109]. Copyright (2019) Elsevier B. V.

P–O bond cleavage is reaction direction that corresponds to catalytic ROP of ethylene phosphates (Scheme 3b). Very recently, we made DFT modeling of TBD-catalyzed polymerization and transesterification of MeOEP [109,110] at the B3PW91/DGTZVP level of theory [111,112]. We performed DFT modeling of TBD-catalyzed reaction of MeOEP with EtOH for both alternative mechanisms while taking into account that the amide mechanism (Scheme 4a) was proposed for TBD-catalyzed polymerization of ROEP [7,113,114] (Scheme 4). The comparison of the reaction profiles of “amide” (Figure 25, left) and “donor-acceptor” (Scheme 4b, Figure 25, right) pathways demonstrated that the “donor-acceptor” mechanism was highly favorable by the difference in activation barriers about 26 kcal/mol.

**Scheme 5:**
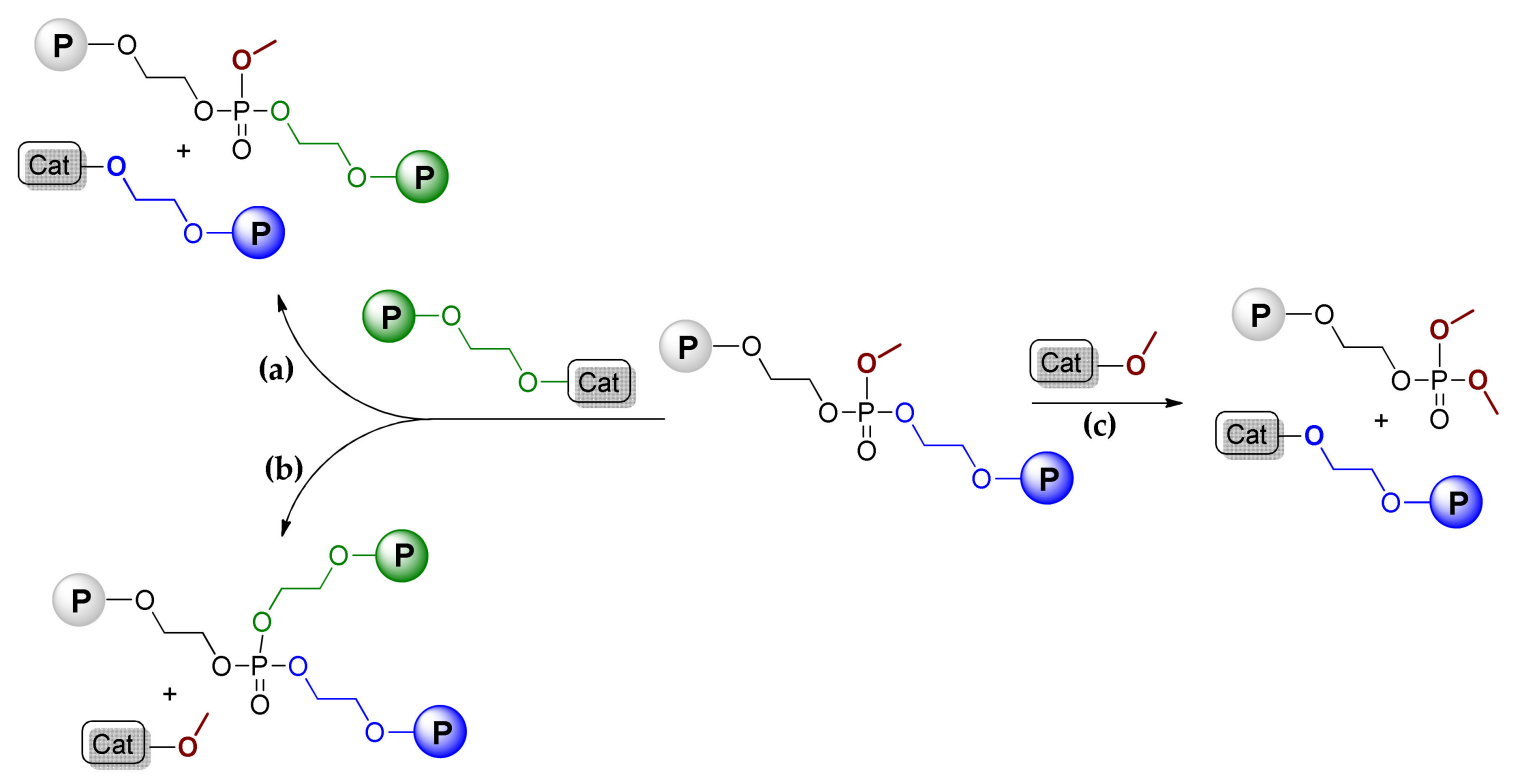
“Scission” (**a**,**c**) and “branching” (**b**) transesterification pathways for poly(MeOEP) [109].

The further DFT modeling of TBD-catalyzed transformations of phosphates was devoted to TBD-catalyzed transesterification (TE) processes. Two fundamentally different transesterification mechanisms of poly(MeOEP) are possible [113,115]. The “scission” route (Scheme 5a) results in the formation of a new linear polymer and macroinitiator. “Branching” (Scheme 5b) results in the formation of a branched polymer and methoxy initiator. The methoxy initiator can react with polyphosphate, yielding a low MW polymer with –OP(O)(OMe)_2_ end-group and macroinitiator (Scheme 5c).

**Figure 26:**
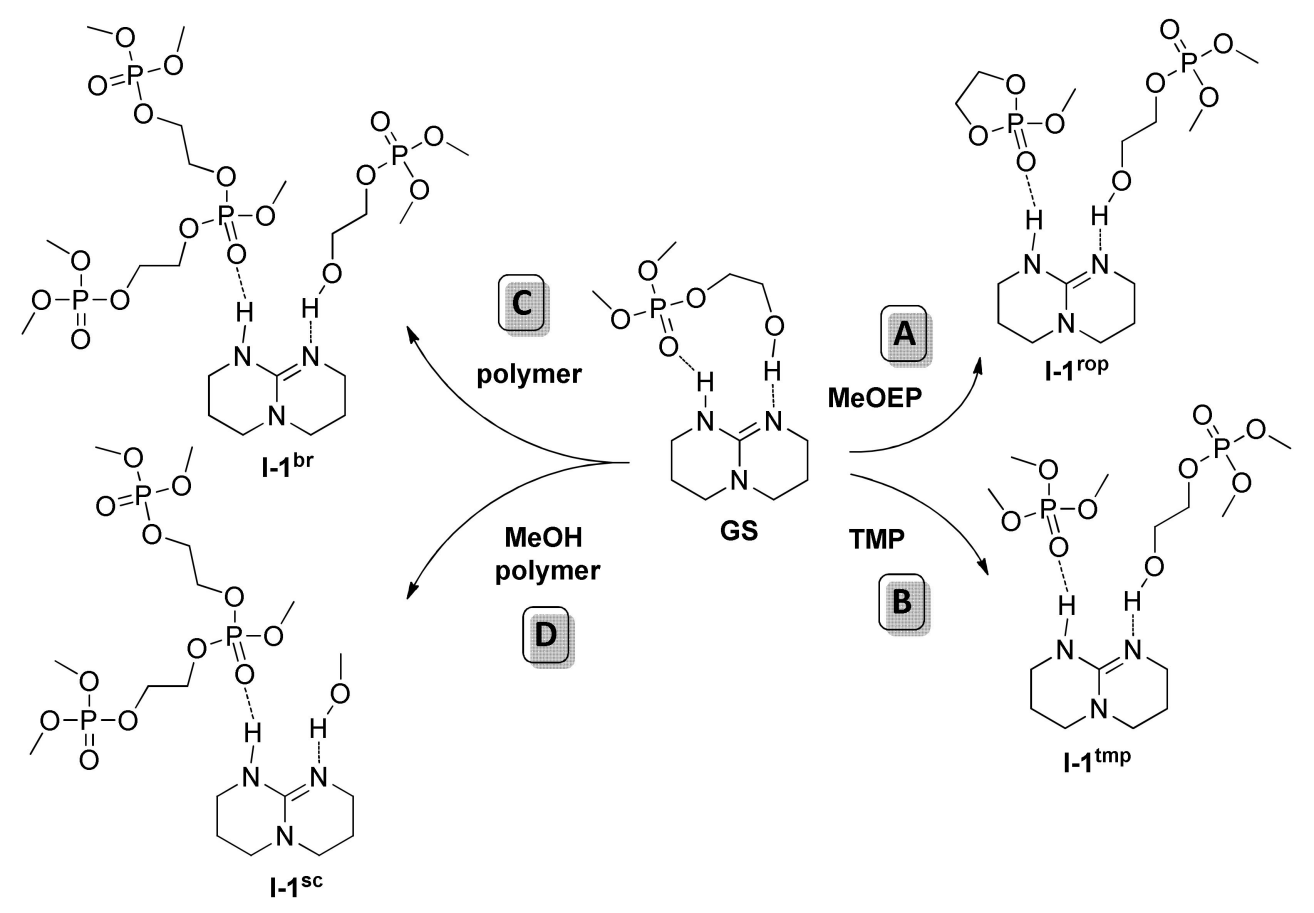
Common ground state and starting stationary points for the TBD-catalyzed ROP of MeOEP (**A**), reaction with TMP (**B**), and transesterification (**C**, **D**) [109].

In view of Scheme 5, DFT modeling was performed by considering two types of initiators (the macroinitiator HOCH_2_CH_2_OP(O)(OMe)_2_ and the methoxy initiator) and two types of substrates (MeOEP and MeOP(O)[OCH_2_CH_2_OP(O)(OMe)_2_]_2_ mimicking the polyphosphate chain fragment). In addition, we calculated the reaction profile for the reaction of the macroinitiator with trimethyl phosphate TMP. The chelate complex of HOCH_2_CH_2_OP(O)(OMe)_2_ and TBD was considered as common ground state for all of the reactions studied (Figure 26)

**Figure 27:**
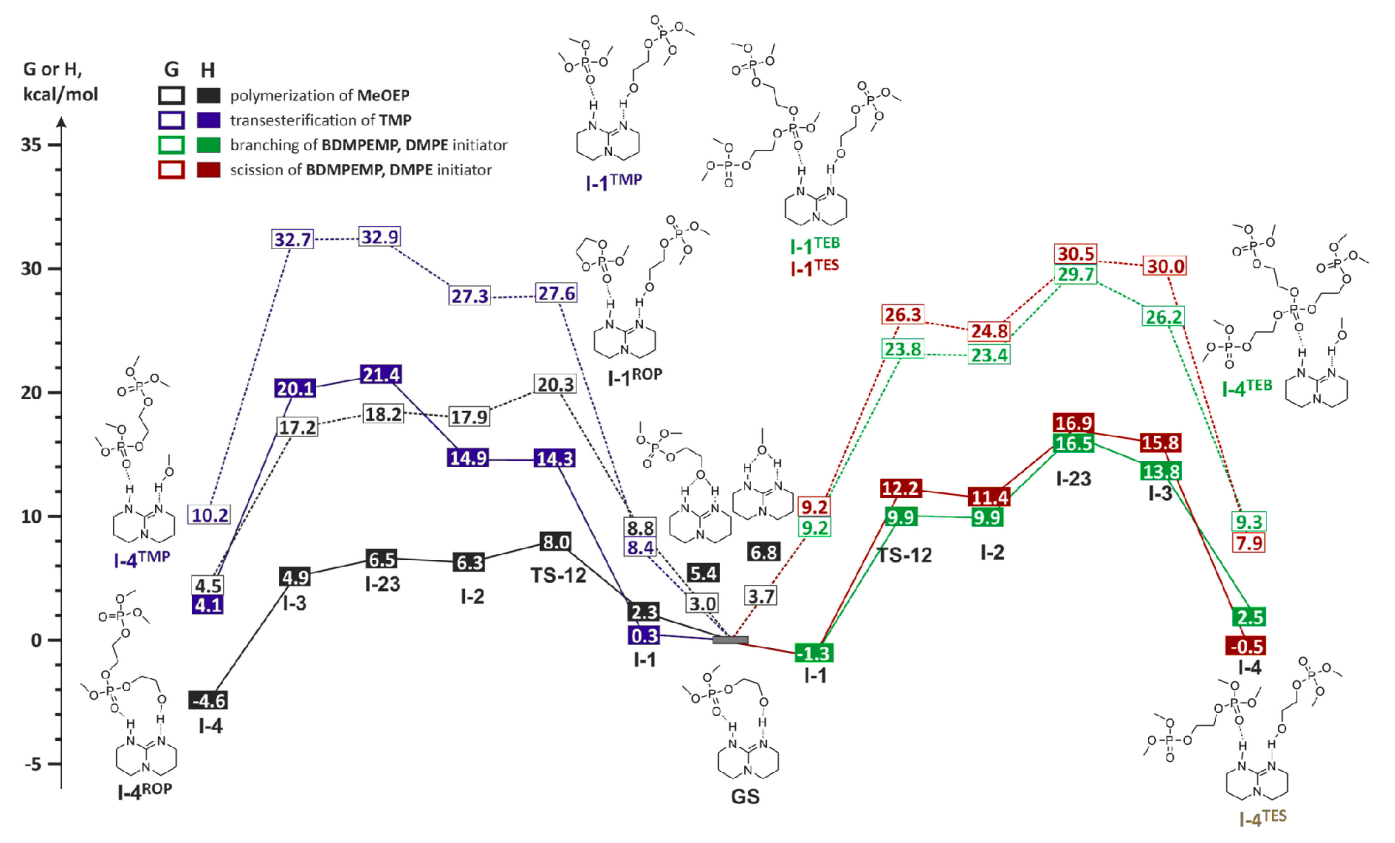
Calculated energy profiles of the TBD-catalyzed ROP of MeOEP and the transesterification of TMP and poly(MeOEP) in G scale (- - - -) and in H scale (——). Reprinted with permission from [109]. Copyright (2019) Elsevier B. V.

We found that the reaction profiles for the TE process of MeOP(O)[OCH_2_CH_2_OP(O)(OMe)_2_]_2_ “branching” and “scission” (Figure 27, right), as well as for transesterification of TMP (Figure 27, left), appear to be the same. All of the TE profiles were markedly different from the profile of the MeOEP ROP of (Figure 27, left), and the free activation energy of the ROP was 10–13 kcal/mol lower than the free activation energies of the TE reactions. The calculated value of the activation barrier of MeOEP polymerization (ΔG ≠ ~20 kcal/mol) corresponded to the experimentally observed fast process [115]. However, the calculations predicted the relative inertness of poly(MeOEP) and TMP in transesterification (ΔG ≠ >30 kcal/mol). The results of calculations were in contradiction with the experimentally observed fast formation of branched poly(MeOEP), even at −20 °C [115].

The transesterification of TMP was experimentally studied to verify the calculations. No TE products were observed; moreover, the addition of TMP to the reaction mixture containing MeOEP, TBD, and alcohol initiator resulted in the formation of linear poly(MeOEP). According to the calculated reaction profiles (Figure 27), MeOEP, TMP, and the fragment of the poly(MeOEP) chain exhibited the same ability to coordinate TBD molecules. The values of the free activation energy of transesterification for TMP and MeOP(O)[OCH_2_CH_2_OP(O)(OMe)_2_]_2_ were also close. However, the open polymer chain model that was used in the evaluation of the activation barrier of polyphosphate transesterification did not take into account the possibility of attractive interaction between the fragments of macromolecules with the formation of polymer globules. The contribution of such interactions is able to reduce the entropy component of the free activation energy. If ΔS ≠ is approaching zero, the ΔH ≠ is approaching ΔG ≠ in its value, and the reaction profile of the ROP in the G scale is moving closer to the profiles of the transesterification of the polyphosphate in the H scale. Figure 27 illustrates this effect for the calculated reaction profiles of MeOEP polymerization (left, in black) and poly(MeOEP) transesterifications (right).

**Scheme 6:**
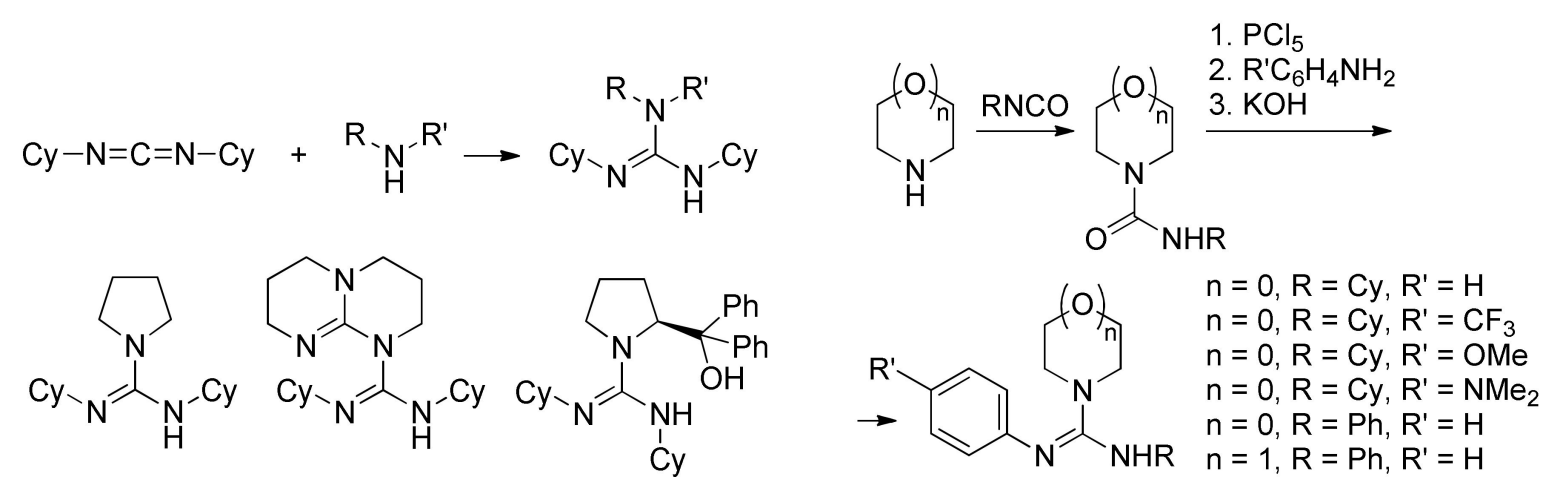
The synthesis and structures of (**a**) dicyclohexyl(alkyl)-substituted guanidines [116] and (**b**) aryl-substituted guanidines [117] studied in *l*-LA ROP.

Hedrick et al. [116], who prepared a number of trisubstituted guanidines by the reaction of secondary amines with dicyclohexylcarbodiimide, demonstrated the further development of guanidine-type catalysts (Scheme 6a). DFT optimization at B3LYP/6-31+G* [40,78,92,93] level of the molecular structures of pyrrolidine derivative, its complex with MeOH, and tetrahedral intermediate formed by the reaction of MeOH with *l*-LA, allowed for explaining the lower catalytic activity of acyclic guanidines relative to TBD.

Five years later, Hecht et al. [117] synthesized a series of aryl-substituted guanidines (Scheme 6b) and studied the catalytic activity of these compounds in polymerization of *l*-LA. It was found that the activity of the catalysts increases in line with increasing of the donor character of the substituent R’; the replacement of acceptor CF_3_ group by donor NMe_2_ group enhanced the catalytic activity by two order of magnitude. The DFT modeling of the aryl-substituted guanidines and their MeOH complexes was performed at B3LYP/TZVP/PCM [39,40,82,87] level; calculated basicities of guanidines and dissociation energies of guanidine-MeOH complexes correlated with the rates of *l*-LA ROP.

## 6. DBU and Related Compounds

**Scheme 7:**
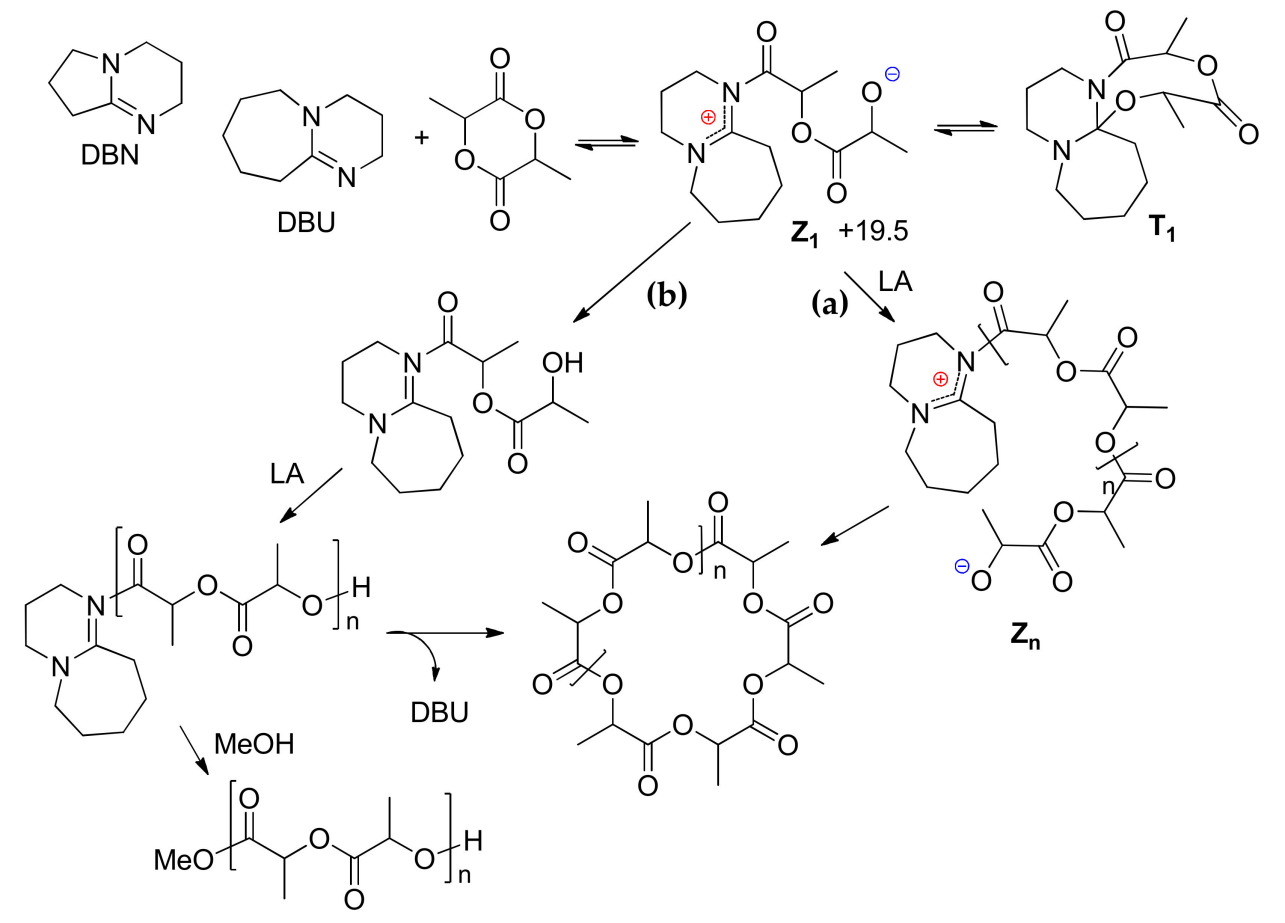
Proposed mechanism for the 1,8-diazabicyclo[5.4.0]undec-7-ene (DBU)-mediated polymerization of lactide [123].

2,3,4,6,7,8,9,10-Octahydropyrimido[1,2-*a*]azepine, which is commonly referred to as 1,8-diazabicyclo[5.4.0]undec-7-ene (DBU) (Scheme 7), is a commonly used organic base with low nucleophilicity. In the presence of ROH initiators, DBU demonstrated moderate catalytic activity in ROP of TMC [118], and good productivity in the polymerization of lactide [119] and ethylene phosphates [113]. From the synthetic viewpoint, the DBU/ROH catalytic system is not the “best choice” for controlled ROP of cyclic esters, except highly active ethylene phosphates [113,120,121,122].

However, DBU without any proton-containing activators was found to be an effective catalyst of lactide ROP with the formation of macrocyclic PLA [123]. In this work, Waymouth et al. studied the ROP of *l*-LA that was catalyzed by DBU and closely related 1,5-diazabicyclo[4.3.0]non-5-ene (DBN) (Scheme 7) and detected the formation of macrocyclic products and minor amounts of linear polylactides that were terminated with MeO groups after treatment of the reaction mixtures with MeOH. It was proposed that DBU acted as an initiator of zwitterionic ROP in analogy to NHCs in the absence of ROH. This assumption was confirmed by DFT modeling of the zwitterionic mechanism (Scheme 7, path a) and an alternative mechanism that included the formation of ketene aminal intermediate (**KA**, Scheme 7, path b) at M06/6-31+G(d,p) [51,124] level of theory with CPCM solvation model correction [82]. The DFT modeling resulted in the finding of several pathways for zwitterionic intermediate **Z_1_**. The first was reversible ring closure to the neutral tricycle **T_1_**, which would likely be a dormant species. The addition of lactide to the alkoxide of the zwitterion would result in chain growth to larger zwitterions **Z_n_** (path a), followed by cyclization with a release a cyclic polylactide and DBU. Alternatively, the deprotonation of the zwitterion could generate **KA** (path b). In the presence of DBU, **KA** could undergo chain growth to generate linear polylactides that are able to form both cyclic PLAs and the MeO-terminated products upon methanol workup. The DFT calculations indicated that all the intermediates **Z_1_**, **T_1_**, and **KA** are energetically accessible; the higher barriers associated with the formation and methanolysis of **KA** was consistent with the formation of minor amounts of linear chains experimentally observed.

**Figure 28:**
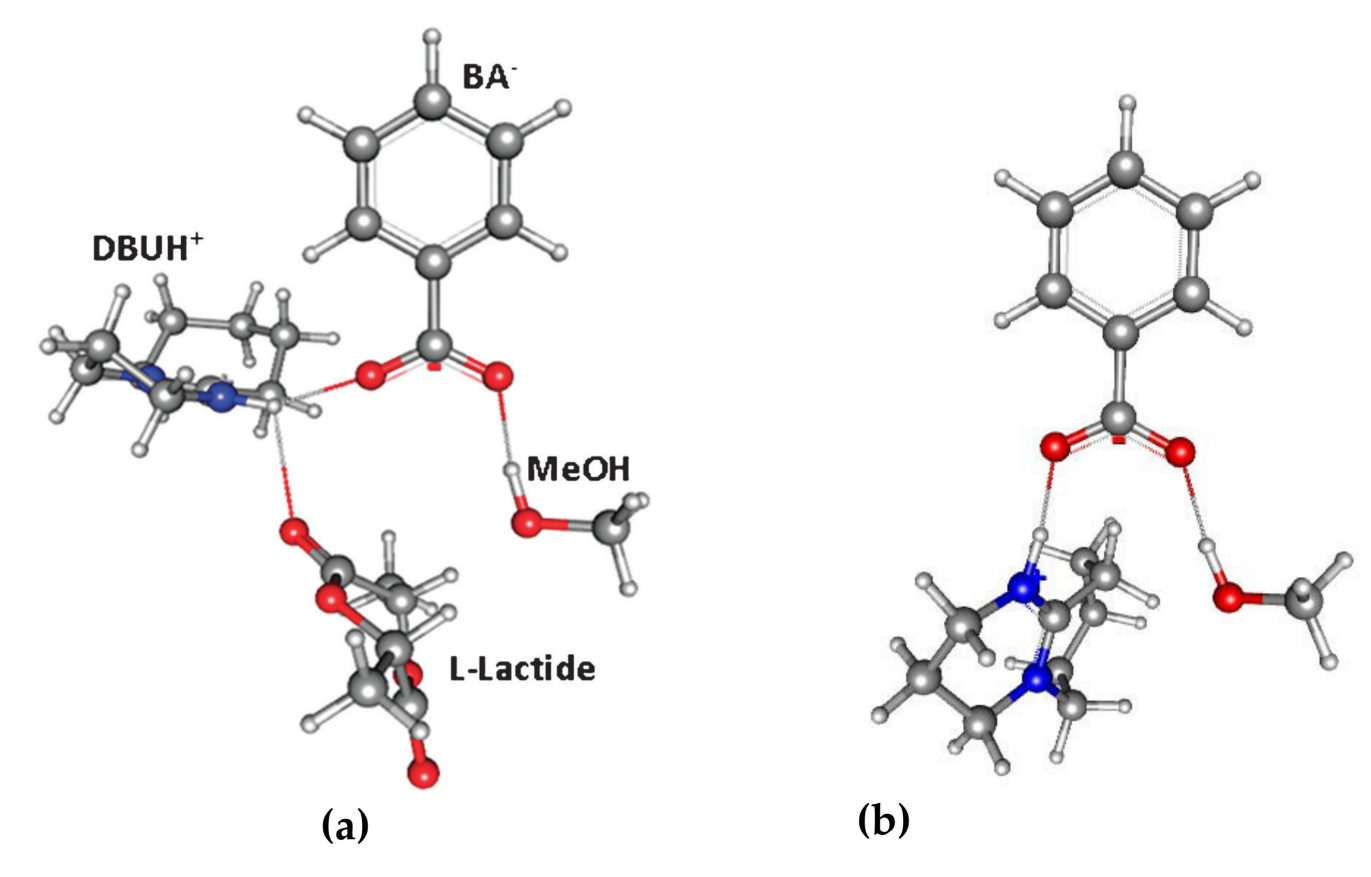
The moleular structures of the key intermediates in DBU/PhCOOH-catalyzed *l*-LA ROP, (**a**) quadruple DBU–PhCOOH–*l*-LA–MeOH reaction complex; (**b**) triple DBU–PhCOOH–MeOH complex that mimics active catalytic species. Reprinted with permission from [125]. Copyright (2011) Royal Society of Chemistry.

Hedrick et al. investigated the use of DBU/PhCOOH complex in alcohol-initiated ROP of *l*-LA was investigated [125]; this study included DFT modeling of the key reaction species at B3LYP/aug-VTZP/aug-cc-pVTZ [39,40,126,127] level while using the IEF-cPCM solvation model (CH_2_Cl_2_) [81]. It was found that, in the presence of benzioc acid, the PLA with harrow *Ð_M_* (1.06–1.07) formed. The DFT modeling clarified the role of the PhCOOH that formed the reaction complex, including DBU, PhCOOH, *l*-LA, and MeOH molecules, in this complex the molecules of cyclic substrate and MeOH are activated by hydrogen bonding (Figure 28a). The 1:1:1 DBU–PhCOOH–MeOH (Figure 28b) represented an active catalytic species. Thus, the use of benzoic acid effectively suppressed the transesterification process; this finding is of great importance for the issue of the organocatalytic ROP.

## 7. Thiourea-Based Catalysts

**Scheme 8:**
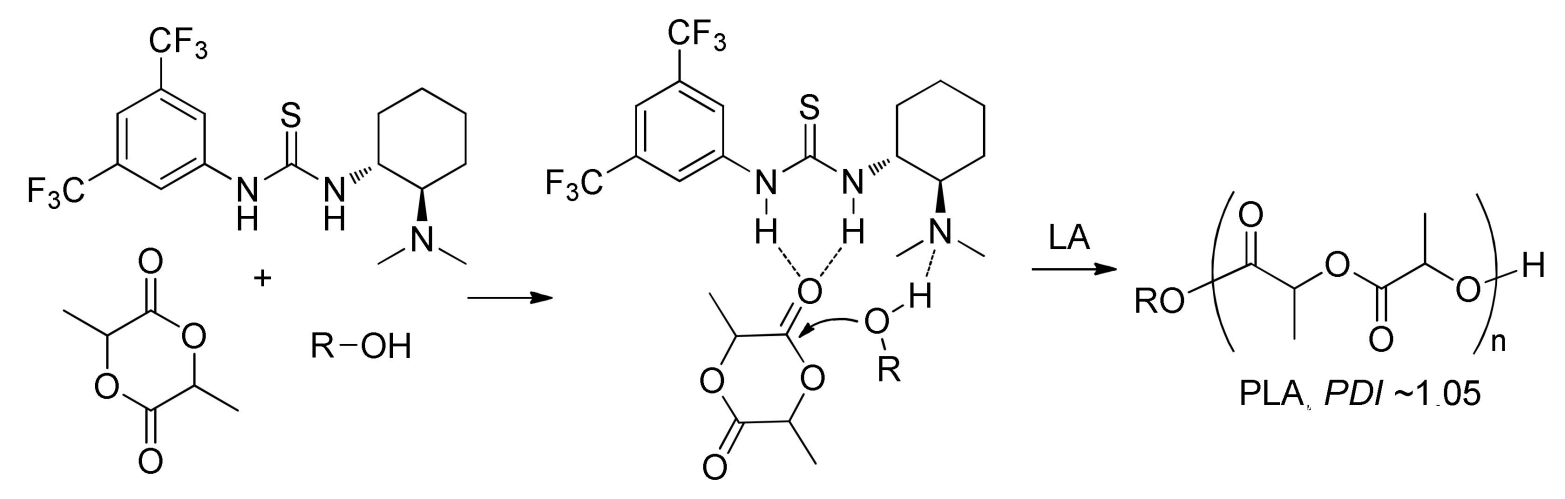
The proposed mechanism of dual activation by amino-functionalized thiourea catalyst [129].

The mechanistics aspects of thiourea-based ROP catalysis have been recently reviewed [128]. In 2005, Waymouth, Hendrick et al. demonstrated unique catalytic behaviour of the amino-functionalized thiourea organocatalyst (Scheme 8) in lactide polymerization [129]. This catalyst demonstrated the extraordinary selectivity for polymerization relative to transesterification; the proposed reaction mechanism involved the bifunctional activation of the LA carbonyl via hydrogen bonding to the thiourea group and of the initiating/propagating alcohol by the Brønsted basic tertiary amino group (Scheme 8). In the further research of this group, it was demonstrated that the combination of thiourea with terniary amines represented an effective catalyst for the controlled ROP of lactides and lactones [119].

**Figure 29:**
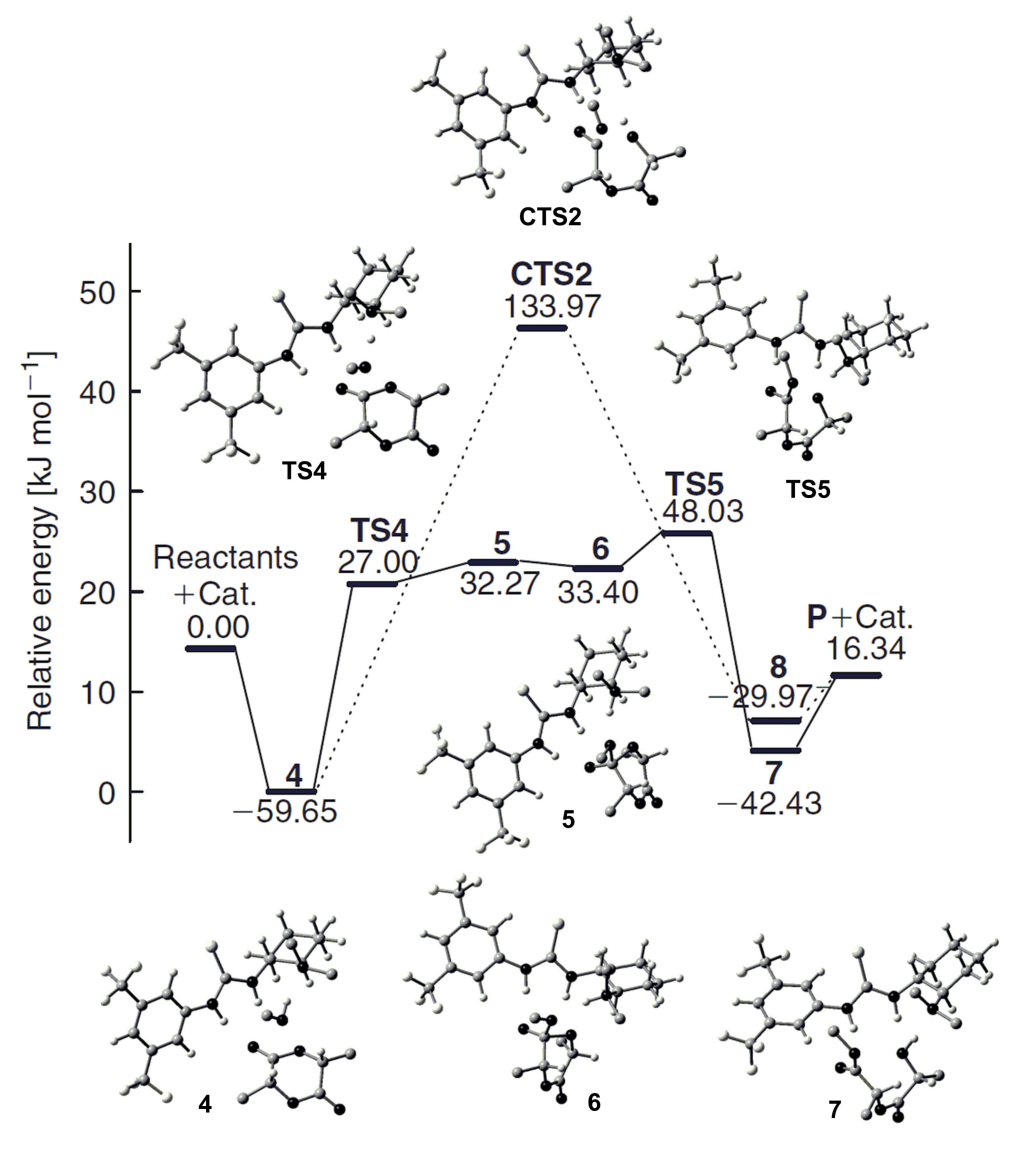
Energy profile (electronic energies with zero-point corrections) for *l*-LA methanolysis catalyzed by amino-functionalized thiourea. Reprinted with permission from [130]. Copyright (2009) Australian Chemical Society.

In 2009 [130], Znang et al. performed DFT modeling of methanolysis of *l*-LA, which was catalyzed by amino-functionalized thiourea organocatalyst (Scheme 8) at B3LYP/6–311++G(d,p) [39,40,131] level of theory. They found that the activation barrier was about 43 kcal/mol for non-catalytic reaction of *l*-LA with MeOH, whereas the activation energy of the catalytic reaction via the formation of triple hydrogen-bonded complex was 25.7 kcal/mol (Figure 29). The comparative calculations of the alternative “concerted” catalytic mechanism through CTS2 allowed for excluding the latter reaction pathway by high activation barrier (Figure 29). The zwitterionic mechanism was also modeled and excluded for the same reason.

**Figure 30:**
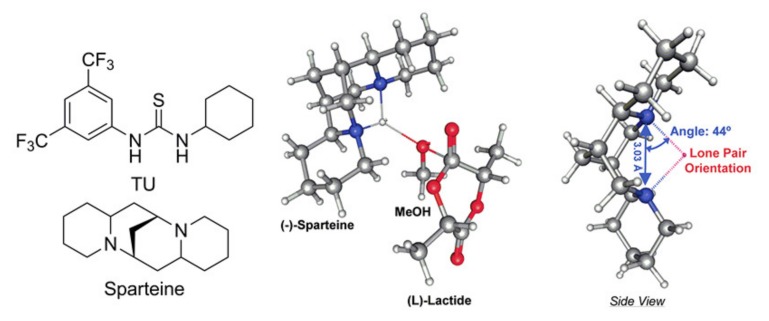
Thiourea (TU) and (–)-sparteine molecular structures and approximate geometry of the rate limiting step of (–)-sparteine catalyzed *l*-LA ROP with methanol showing no dependence on catalyst chirality, and the calculated distance between N atoms along with the lone pair angle extending from the N–N plane. Reprinted with permission from [132]. Copyright (2010) American Chemical Society.

Hedrick et al. established a very close mechanism for thiourea (TU) and spartein-catalyzed alcohol-initiated ROP of *l*-LA [132]. It was found by DFT calculations that it was the lone pair orientation in spartein molecule (Figure 30) provided an effective activation of MeOH molecule. Such an activation resulted in extremely high catalytic activity. Kazakov and Kiesewetter analyzed some aspects of catalytic behaviour of amine-TU catalysts [133], using DFT modeling for the optimization of amine-TU complexes.

**Figure 31:**
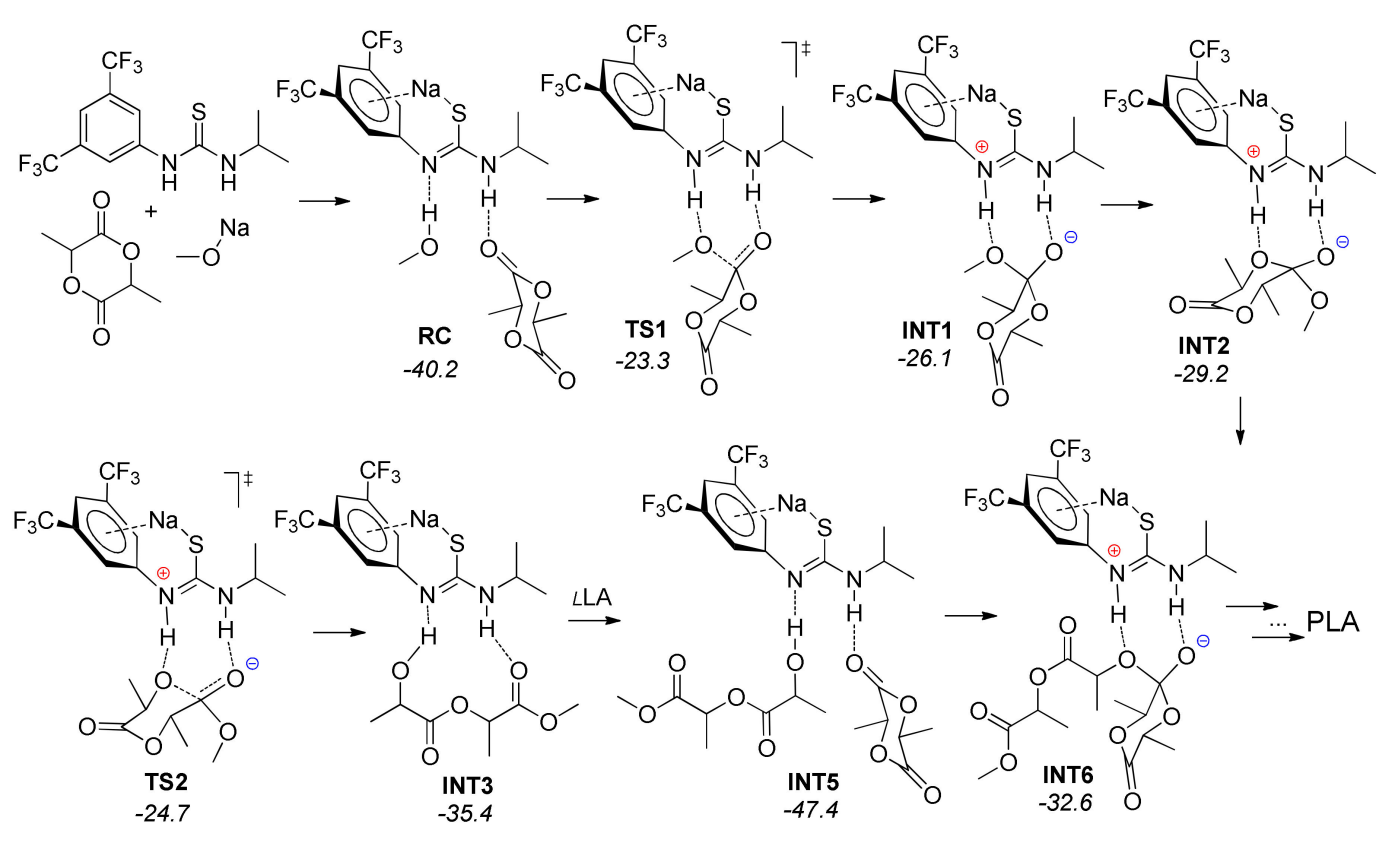
The structures and calculated free energies of the stationary points and transition states for thioamidate-catalyzed ROP of *l*-LA [134].

Finally, Waymouth et al. effectively used the DFT methods to explain the outstanding catalytic characteristics of TU-derived anion—thioamidate—in *l*-LA ROP [134]. The catalysts containing this anion demonstrated higher activity in comparison with the MeONa initiator, and provided the obtaining of PLA with narrow MWD. In contrast with the mechanism that included the formation of three-component catalytic species TU–amine–ROH, as discussed above, the TU-derived thioamidate acts as a dual hydrogen-bonding activator for both *l*-LA and MeOH molecules. DFT modeling of the reaction profile was performed at B3LYP-D3/6-31+G(d)/aug-cc-pVTZ [39,40,78,127,135,136]; the impact of the solvent (CH_2_Cl_2_) was taken into account with the IEF-cPCM method [137]. The isopropyl-substituted model was used for calculations instead of TU molecule; Figure 31 presents the structures of the stationary points and transition states with the corresponding free energies. Note that the binding of *l*-LA molecule to the MeOH-bound thioimidate (**INT5**) was ~12 kcal/mol more favorable than binding of the open-chain ester (INT3). The mechanism that is presented in Figure 31 is very similar to the donor-acceptor mechanism of TBD-catalyzed ROP of lactones and lactides (Figure 14).

**Figure 32:**
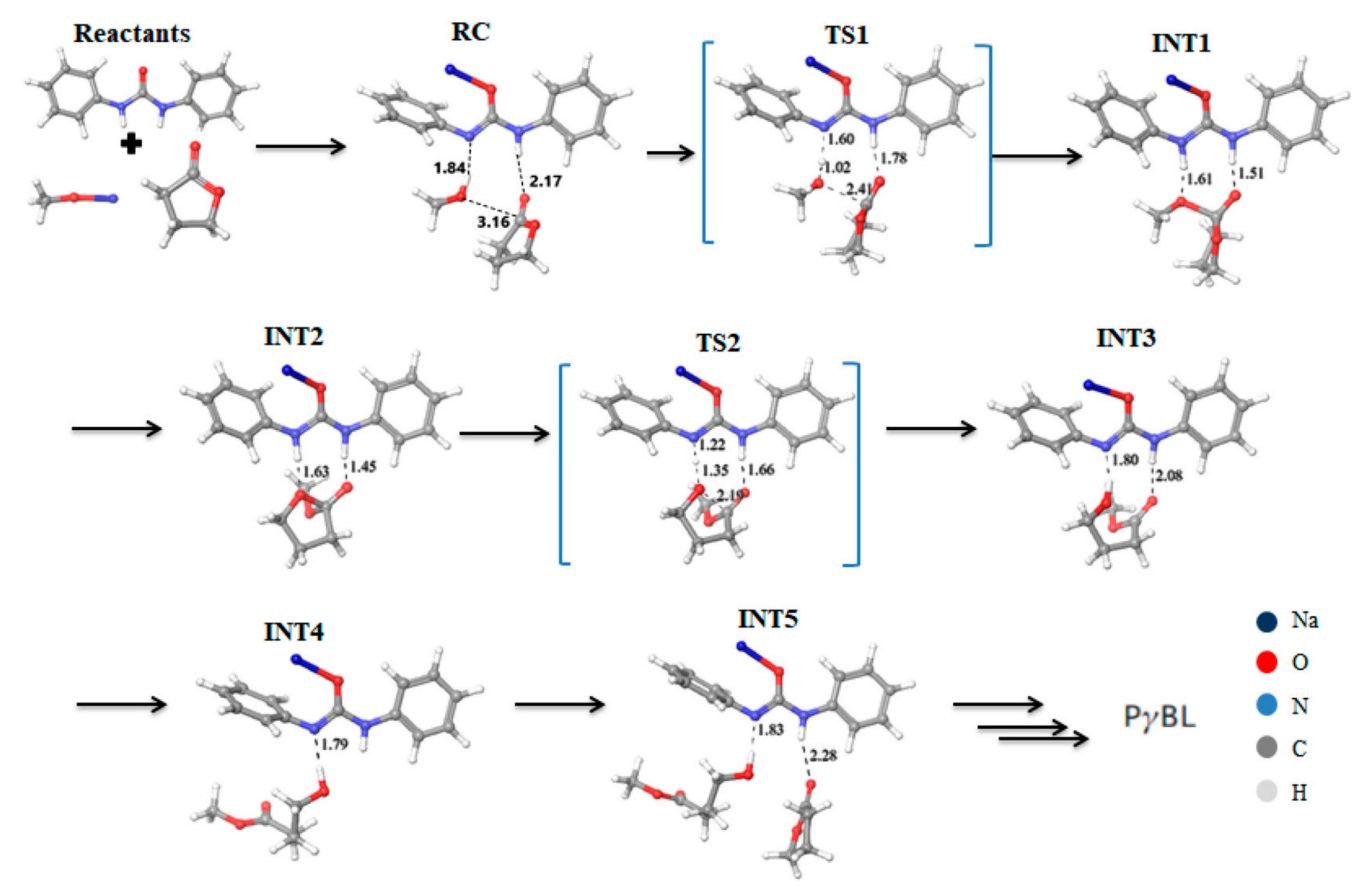
The stationary points and transition states of the proposed mechanism for the polymerization of γBL catalyzed by *N*,*N*’-diphenylurea/MeONa. Bond distances are exposed in the black dashed line. Reprinted with permission from [143]. Copyright (2018) American Chemical Society.

**Figure 33:**
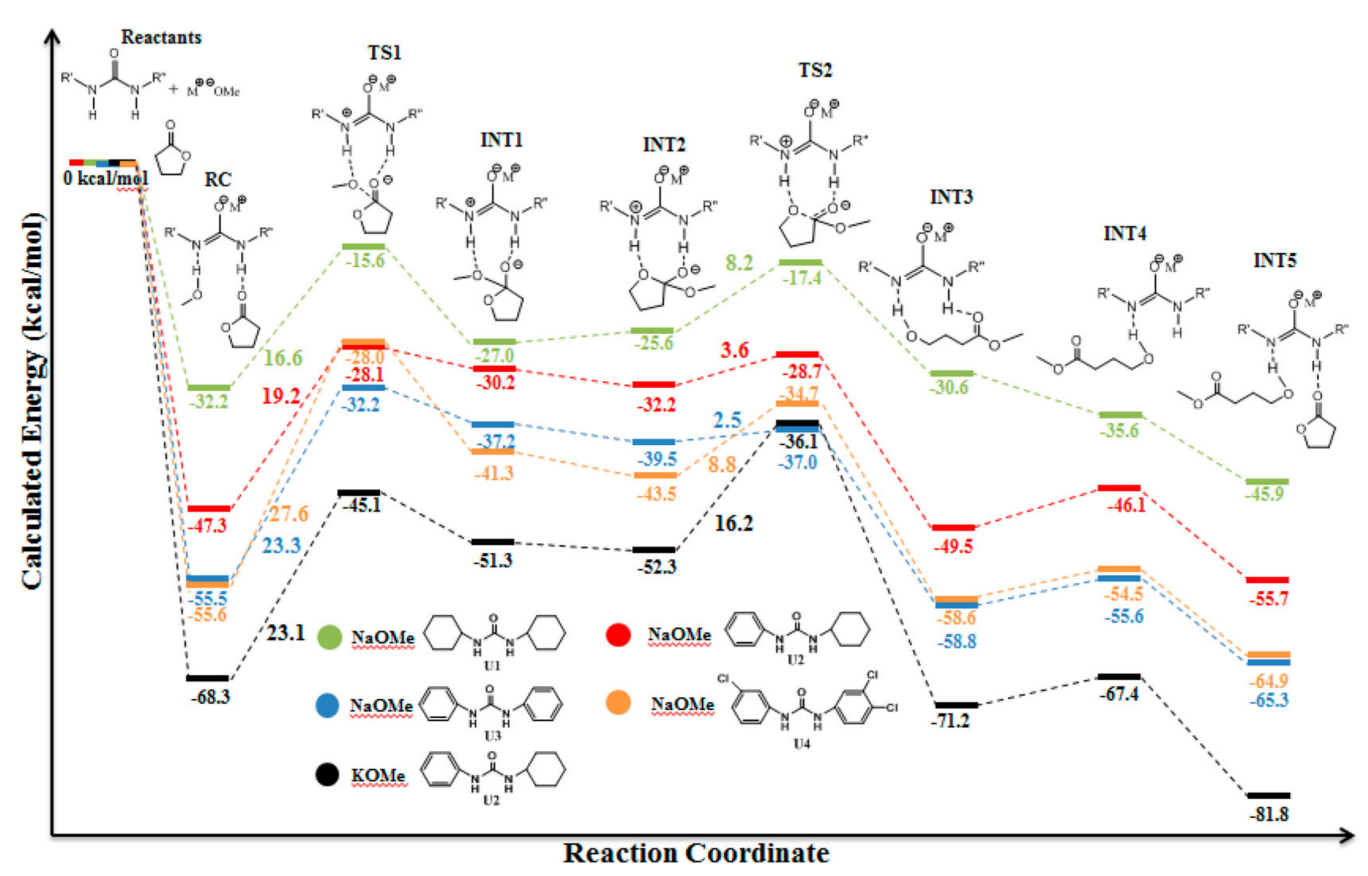
The free energy reaction profiles for different ureas and alkoxides. Reprinted with permission from [143]. Copyright (2018) American Chemical Society.

The thioimidate catalyst demonstrated good catalytic activity in lactide polymerization, but lower activity in TMC ROP and low productivity in polymerization of lactones. The novel simple and promising catalysts, the close structural analogs of TU-derived thioamidate, were studied in the few past years [138,139,140,141,142]. These catalysts, which represent the metal derivatives of disubstituted ureas, demonstrated high activities in ROP of cyclic esters of all actual structure types—lactide, lactones, TMC, and ethylene phosphates. Moreover, Meng et al. [143] found that these catalysts are effective in the polymerization of the “inert in ROP” [37,46] cyclic ester, five-membered γBL. The problem of the synthesis of HMW poly(γBL) was solved due to high catalytic activity of these catalysts that allowed for running γBL polymerization at very low reaction temperatures. Meng et al. performed a thorough theoretical study of γBL ROP catalyzed by urea anions with different substituents at the nitrogen atoms while using DFT calculations at GGA-PBE [144,145,146] level of theory for an explanation of the experimental results and understanding the reaction mechanism. The results of computational studies (Figure 32 and Figure 33) demonstrated that urea anion acted as a bifunctional catalyst that activated both alcohol molecule and γBL. According to thermodynamic calculation, the more alkaline urea with electron-donating group exhibited lower activation barrier and, therefore, demonstrated higher catalytic efficiency.

## 8. Acid-Catalyzed ROP

The most of organocatalysts have basic functionalities. This has made the polymerization of monomers containing carboxylic, amide, or other acidic functionalities considerably more difficult, if not impossible, while using traditional basic organocatalysts. Acid-catalyzed ROP is highly attractive for such monomers; however, only traditional substrates were experimentally studied in such reactions. The strong Brønsted acids are only effective ROP catalysts for specific substrates, such as 1,3-dioxepan-2-one [147], and only several types of Brønsted and Lewis acids demonstrated the high efficiency in ROP of common cyclic esters, such as δVL, εCL, TMC, or LA. The ability of sulfonic acids [148,149,150,151,152,153,154,155,156,157,158,159], carboxylic acids [160,161,162,163], acidic phosphates [164,165,166,167], ROH/HCl/ether [168,169,170], and Tf_2_NH [171] to catalyze polymerization of the common lactones, cyclic carbonates, and lactides has been established, the mechanisms of the reactions were proposed but not explored in detail. In [150], the thermochemistry of cationic ring-opening of TMC and 5-methylene-1,3-dioxan-2-one was estimated by quantum-chemical modeling at the HF/3-21G** level of theory, but the results of such modeling do not correspond to the subject of our review.

**Scheme 9:**
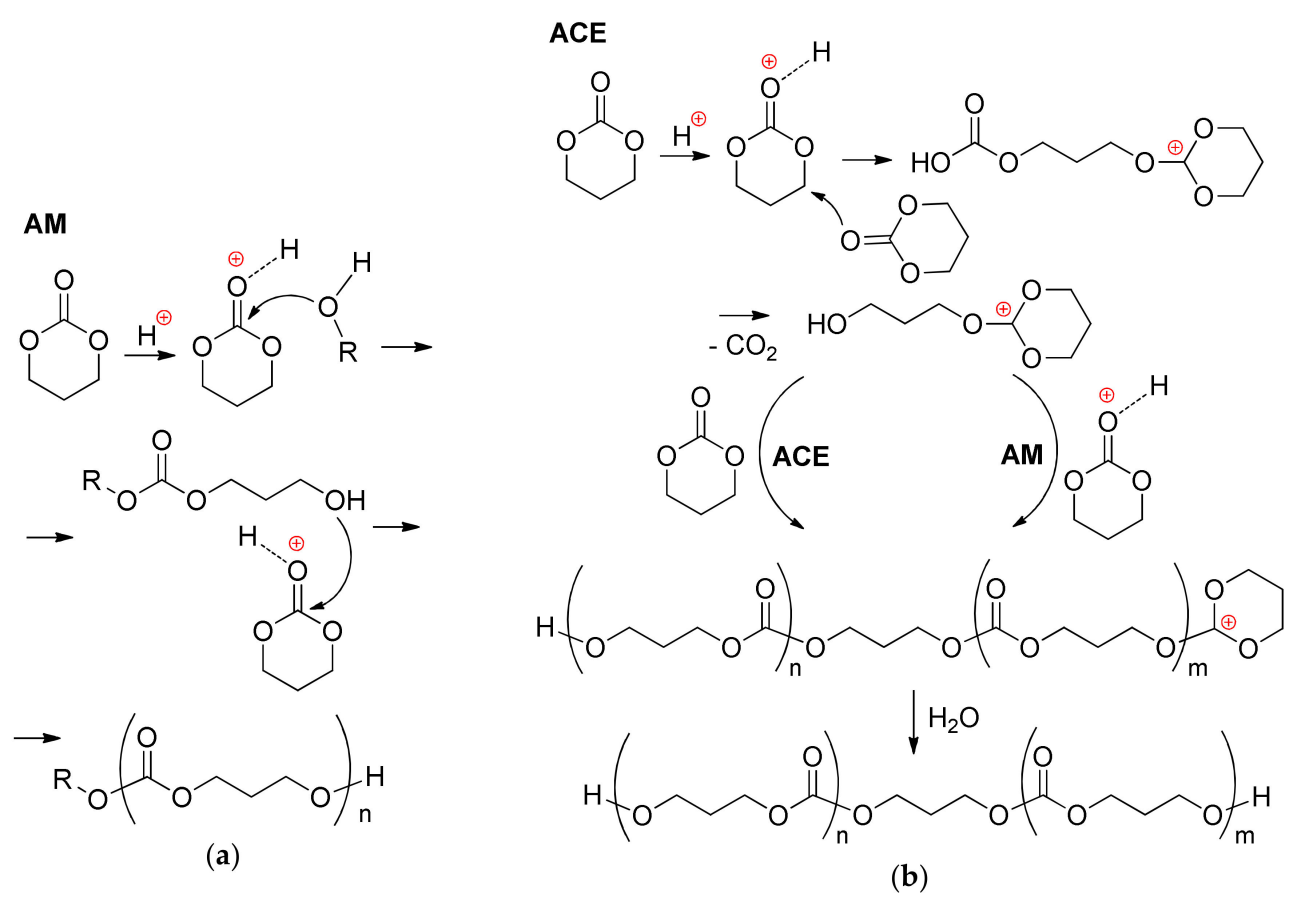
Two mechanisms for acid-catalyzed ROP of TMC: (**a**) Activated monomer mechanism (AM); and, (**b**) Activated chain end mechanism (ACE) [172].

Ten years later, Bourissou et al. showed sulfonic acids as effective polymerization catalysts for TMC ROP [172] and then proposed two reaction pathways (Scheme 9) to explain the formation of bimodal poly(TMC). The activated chain end (ACE) mechanism and activated monomer (AM) mechanism were discussed. Note that Baśko and Kubisa discussed such two mechanisms earlier for εCL and LA acid-catalyzed ROP [155]. However, the first theoretical studies of acid-catalyzed ROP were conducted until 2010.

**Figure 34:**
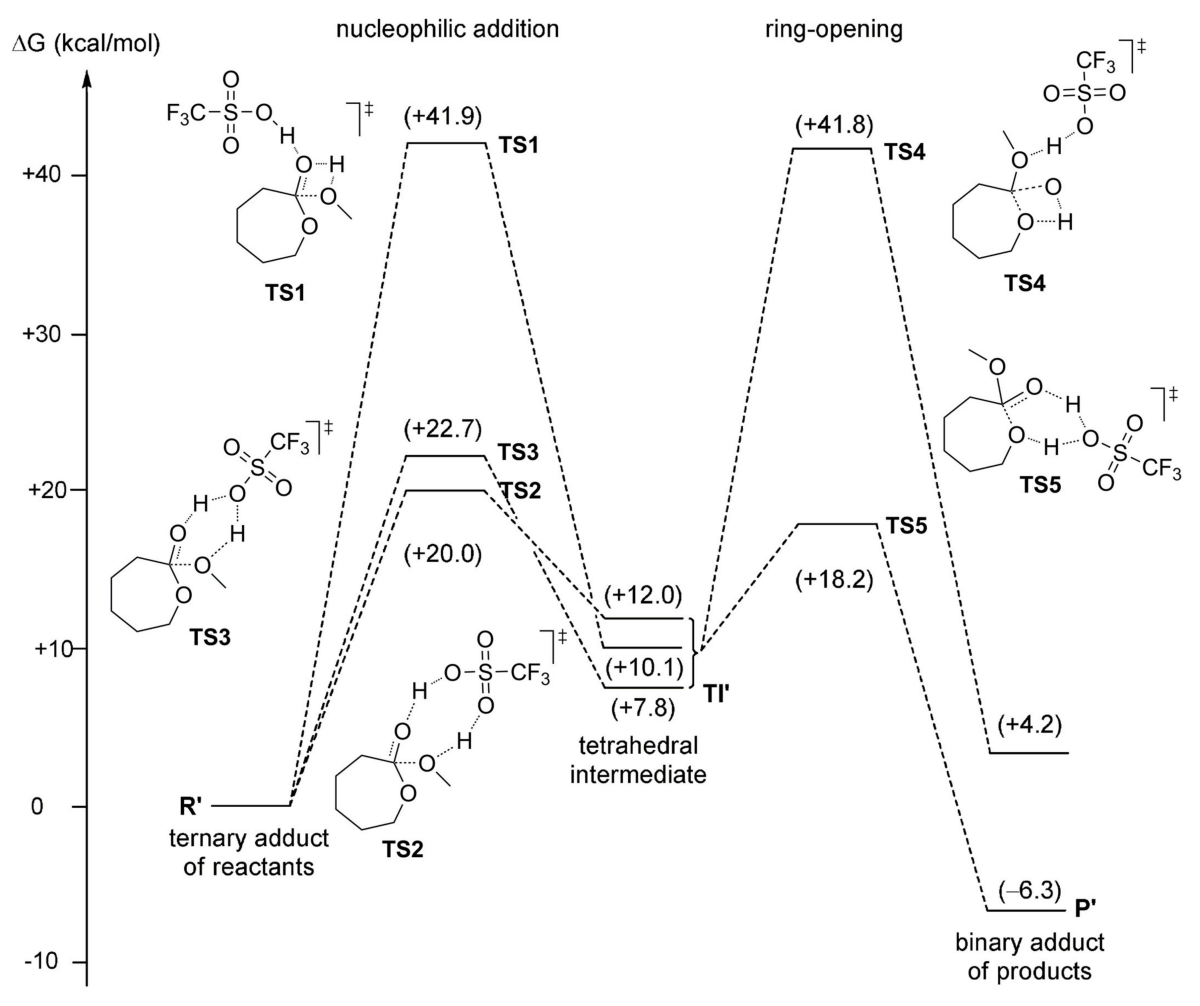
Free energy reaction profiles for alternative mechanisms of CF_3_SO_3_H-catalyzed methanolysis of εCL, and the structures of the key transition states. Reprinted with permission from [173]. Copyright (2010) American Chemical Society.

Maron et al. computationally studied the catalytic properties of CF_3_SO_3_H and MeSO_3_H in εCL ROP initiated by MeOH [173] while using B3PW91 functional [40,111] and 6-31G(d,p) basis set [98]; the F atom was treated with a Stuttgart-Dresden pseudopotential [174]. The nucleophilic addition and ring-opening were analyzed and discussed separately. Three transition states were found for the insertion step, two energetically close bifunctional transition states TS2 and TS3 were found to be preferable by the value of 20 kcal/mol in comparison with monofunctional TS1 (Figure 34). The products of MeOH insertion were tetrahedral intermediates differed by the nature of the hydrogen bonds involved; these intermediates were close by energy (within 3.5 kcal/mol). The ring-opening step involved the cleavage of the endocyclic C–O bond. For this step, two transition states were located, TS4 (without the participance of the acid) and six-membered TS5 (with a alkylsulsonate bridge between the cyclic O atom and hydroxy group, Figure 34). The energy of the ring-opening TS5 was lower than transition states of the initiation step due to the assistance by CF_3_SO_3_H, so that the ring-opening was an easy process. The initial nucleophilic attack was the rate-determining step, and the overall free enthalpy change for εCL methanolysis was estimated by the value of −3 kcal/mol.

In summary, it was clear that sulfonic acid demonstrated a bifunctional behaviour, acting as a “proton shuttle” via its acidic hydrogen atom and basic oxygen atoms. A similar study was performed for MeSO_3_H; the energy reaction profile of this process was found to be similar to the reaction profile that is presented in Figure 34. The activation barriers that were predicted for MeSO_3_H and CF_3_SO_3_H (22.7 and 16.7 kcal/mol, respectively) were in the same range, despite a significant difference in acidity. The slightly higher values found for MeSO_3_H over CF_3_SO_3_H suggested that ring-opening polymerization of εCL should proceed faster when catalyzed by CF_3_SO_3_H, whereas MeSO_3_H was experimentally found to be more active [157]. Such difference between theoretical and experimental data was explained later [175] (see below).

**Figure 35:**
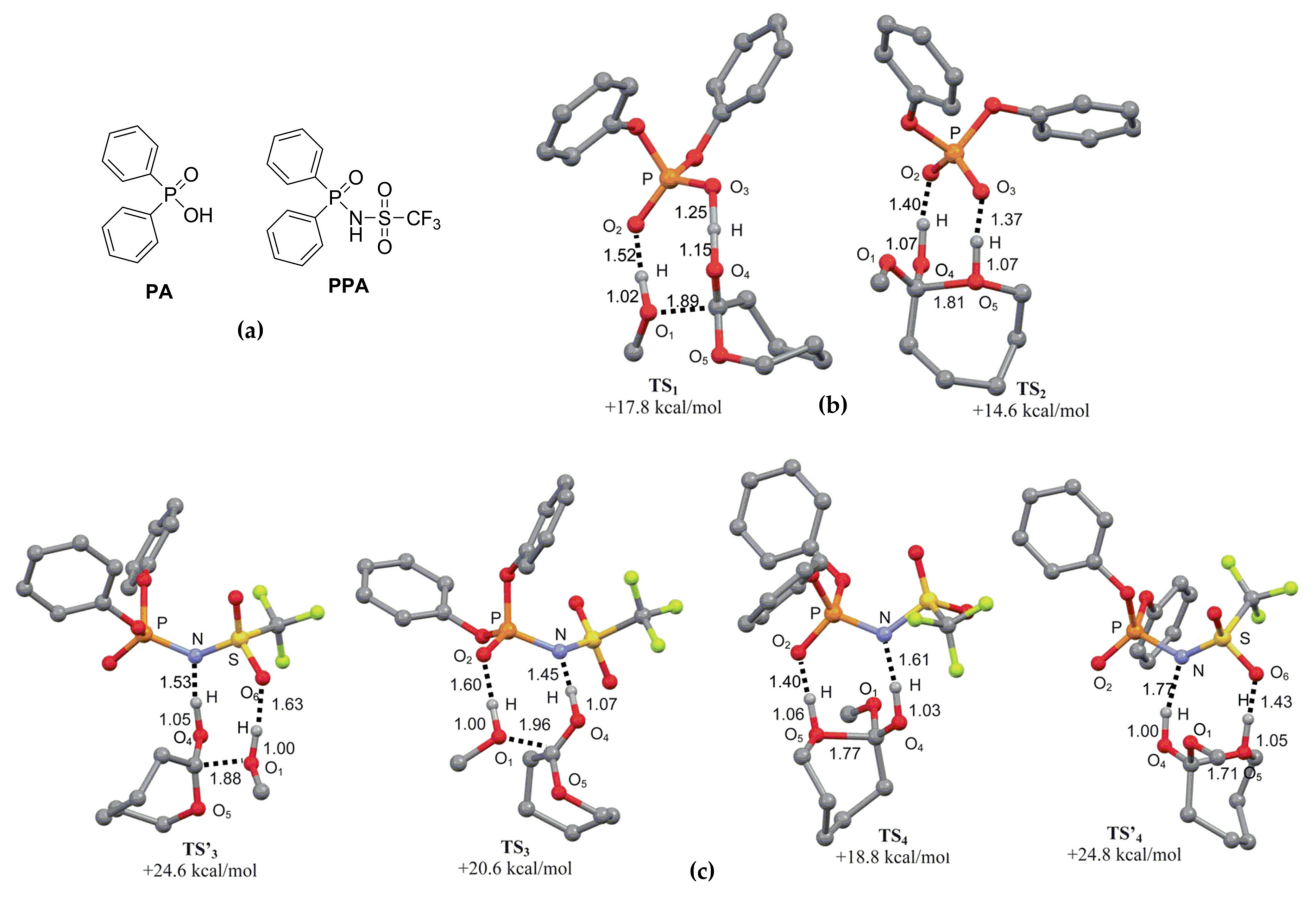
The structures of phosphoric acids (PA) and phosphoramidic acids (PPA) (**a**); the geometries and relative energies of the key transition states for εCL methanolysis catalyzed by PA (**b**) and PAA (**c**). Reprinted with permission from [166]. Copyright (2010) American Chemical Society.

One year later, Bourissou, Maron et al. [166] studied εCL ROP catalyzed by phosphoric acids and phosphoramidic acids (PA and PAA, respectively, Figure 35a). The polymerization experiments in toluene media resulted in obtaining poly(εCL) with narrow Ð_M_ (1.07–1.15); the end-fragments of ROH initiator used (*n*-C_5_H_11_OH) were detected in NMR spectra of polymers; the values of *M_n_* determined by end-group analysis and by SEC were in good agreement; thus, the ROP under these conditions can be considered as a living polymerization. DFT modeling of the reaction mechanisms were performed at B3PW91/6-31G(d,p) [40,98,111] level of theory while using MeOH as an initiator. While taking the bifunctional character of PA and PAA catalysts into consideration, the authors studied and discussed the only bifunctional pathways that were qualitatively different for PA and PAA. Figure 35 also presents the key transition states for the insertion and ring-opening steps. The calculations predicted higher catalytic activity for PA (lower activation barrier), but PAA demonstrated slightly higher productivity in polymerization experiments. Despite this discrepancy, the modeling clearly emphasized the importance of cooperative activation; both systems were acting as proton shuttles.

**Scheme 10:**
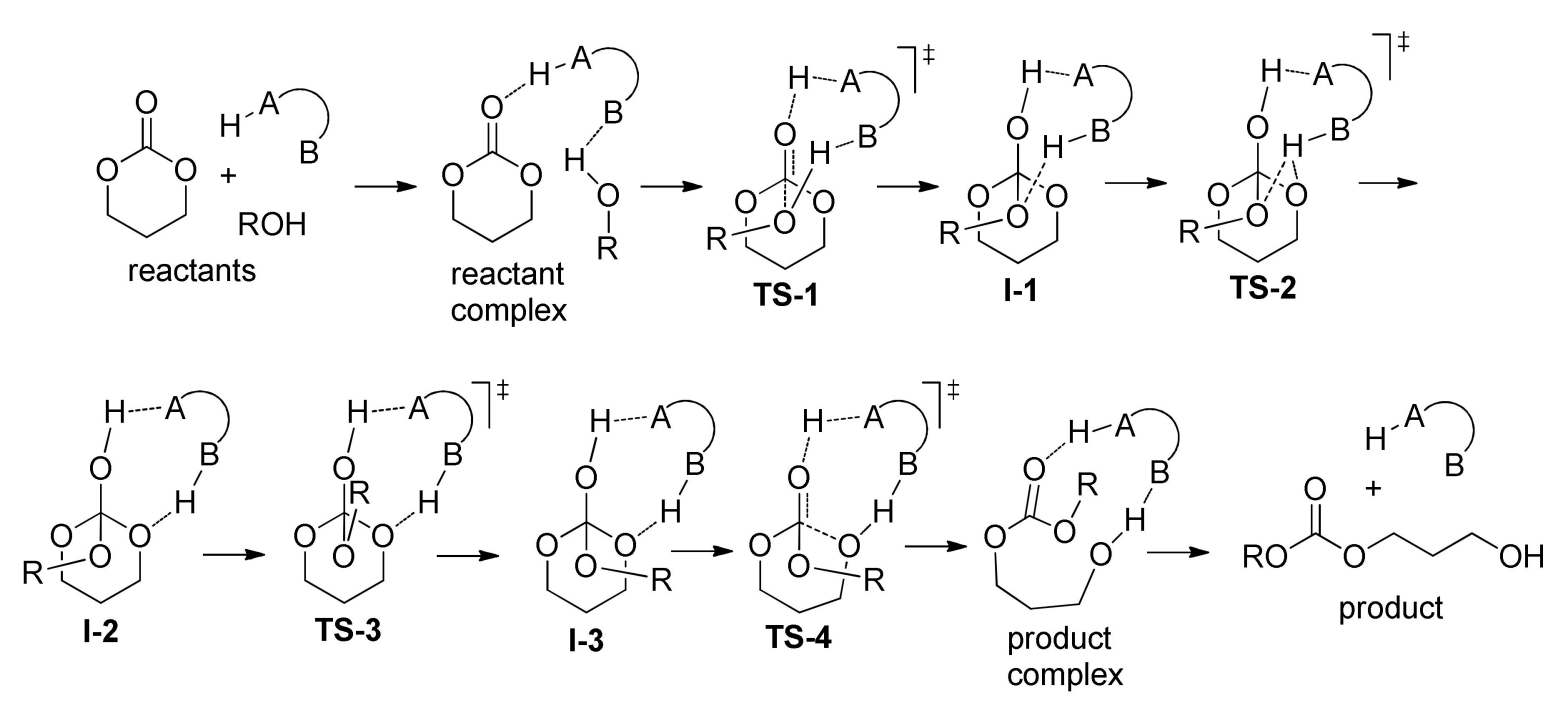
Proposed acid-catalyzed mechanism for the ROP of cyclic carbonates [175].

Coady et al. conducted a comprehensive computational study of possible reaction pathways comparing the sulfonic and carboxylic acids to gain better understanding of the mechanism of acid-catalyzed ROP of TMC [175]. Calculations were performed with GAMESS-US38 [176] while using M11 density functional theory [177] with the 6-311 + G (2d,p) basis set [39], followed by single point energy calculations with the aug-cc-pVTZ basis set [127,136]; the impact of the solvent (CH_2_Cl_2_) was taken into account with the SMD (IEF-cPCM) method [137]. Computation suggested a counterintuitive mechanism: instead of full carbonyl protonation (Scheme 9), a pathway utilizing bifunctional activation was found (Scheme 10).

**Figure 36:**
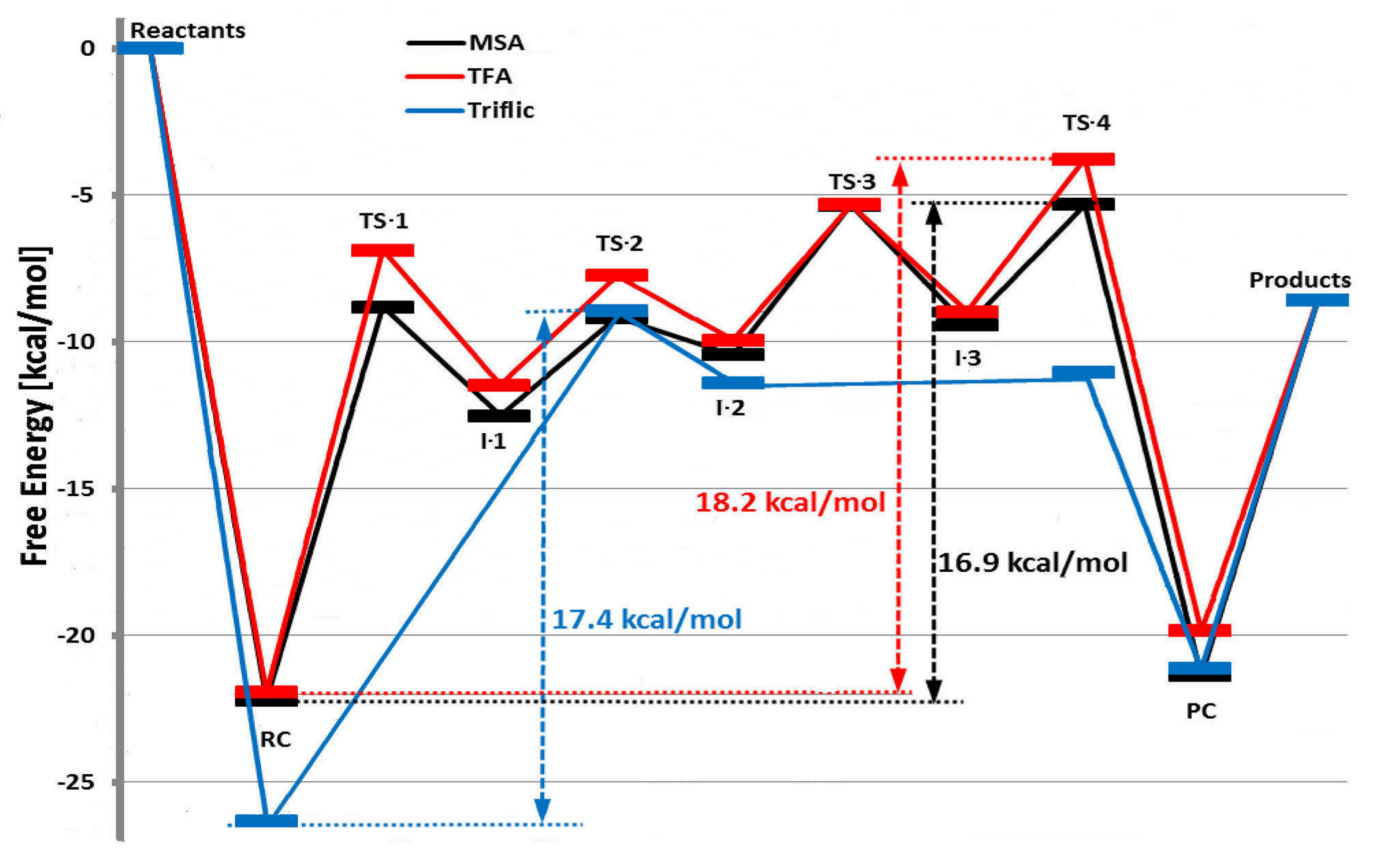
Calculated reaction energy profiles for ROP of TMC initiated by methanol and catalyzed by MeSO_3_H (MSA), CF_3_COOH (TFA), or triflic acid CF_3_SO_3_H. Reprinted with permission from [175]. Copyright (2013) American Chemical Society.

The acid molecule plays the role of such bifunctional activator that consists of an acidic group that is capable of hydrogen bond donation (H–A) and a Lewis basic atom capable of accepting a hydrogen bond (B). The first step is a nucleophilic attack (TS-1) with proton transfer to monomer and formation of I-1. At the second step (TS-1), the proton transfer to exocyclic oxygen atom occurs (I-2). The intermediate obtaining forms I-3 by overcoming rotational TS-3. The next step, ring-opening via TS-4, results in the product complex. At the final stage, the molecule of the product forms; this molecule acts as a reagent in the reaction with the next TMC molecule by the same reaction sequence (propagation stage). Figure 36 presents the calculated reaction profiles of the polymerization of TMC initiated by methanol and catalyzed by MeSO_3_H (MSA), CF_3_COOH (TFA), and triflic acid CF_3_SO_3_H. DFT modeling data were in poor agreement with the experimental results: triflic acid demonstrated the best activity; TFA was inactive as a catalyst.

The comparative DFT modeling of the activated chain end (ACE, Scheme 9) mechanism energetics resulted in the free activation energy value of 45 kcal/mol generally considered as too high for a viable mechanistic alternative. The ROP experiments with complete water removal resulted in a decrease of the high MW fraction of the polymer [175], which invalidated the ACE mechanism that was proposed earlier [172] and confirmed the results of DFT modeling.

**Figure 37:**
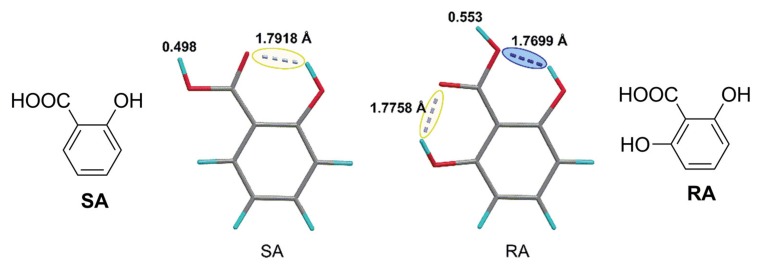
Hydrogen bonding in γ-resorcylic acid (RA) and salicylic acid (SA). Reprinted with permission from [178]. Copyright (2015) Royal Society of Chemistry.

The activated momoner mechanism of ROP had been detected not only for sulfonic acids, but also for carboxylic acids. Guo et al. [178] studied the polymerization of δVL and εCL, catalyzed by γ-resorcylic acid (RA) or salicylic acid (SA, Figure 37), and performed the DFT modeling of the molecular structures of these acids at B3LYP/6-31++G [39,40,41] level of theory. The calculations performed only allowed for the authors to explain higher acidity of RA by double hydrogen bonding (Figure 37); the probability of bifunctional mechanism was only mentioned but not discussed.

**Scheme 11:**
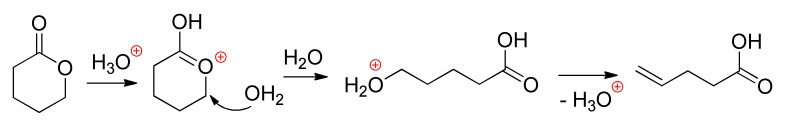
Ring-opening/dehydration of lactones catalyzed by Brønsted acid in water [179].

It is also highly significant that the reaction of the Brønsted acids with lactones—if we do not view the acid catalyst as a bifunctional agent—results in the formation of unsaturated carboxylic acids. Haider et al. studied this issue in detail [179] at the GGA-PW91 [180] level of theory. The results of simulations demonstrates the linear correlation between the rate constants and the energies of the formation of oxocarbenium ions; these ions undergo nucleophilic attack of the water molecule not on carbonyl carbon, but on ω-carbon of the lactone ring, followed by dehydration (Scheme 11).

## 9. Phosphazenes: A Regrettable Lacuna in DFT Modeling

**Scheme 12:**
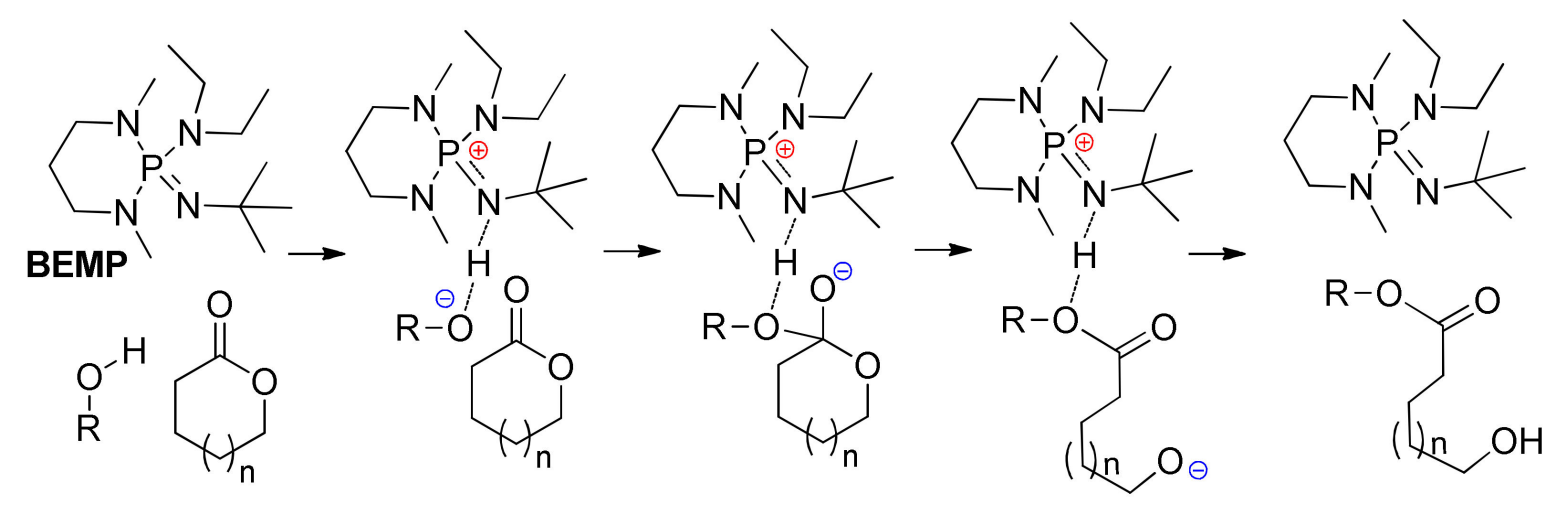
Postulated mechanism of the ROP of lactones using phosphazene BEMP as catalyst [182].

To date, DFT modeling was not applied for theoretical analysis and visualization of the mechanisms of ROP catalyzed by nitrogen–phosphorous hybrid organobases, such as phosphazene bases (PBs, Scheme 12), which possess a remarkably high basicity [181]. Wade et al. proposed the mechanistic concept of phosphazene-catalyzed polymerization of cyclic esters already in the first publication on the theme [182]. This mechanism involves the activation of the alcohol for the nucleophilic attack on the carbonyl group of cyclic esters without the activation of cyclic esters (Scheme 12).

**Scheme 13:**
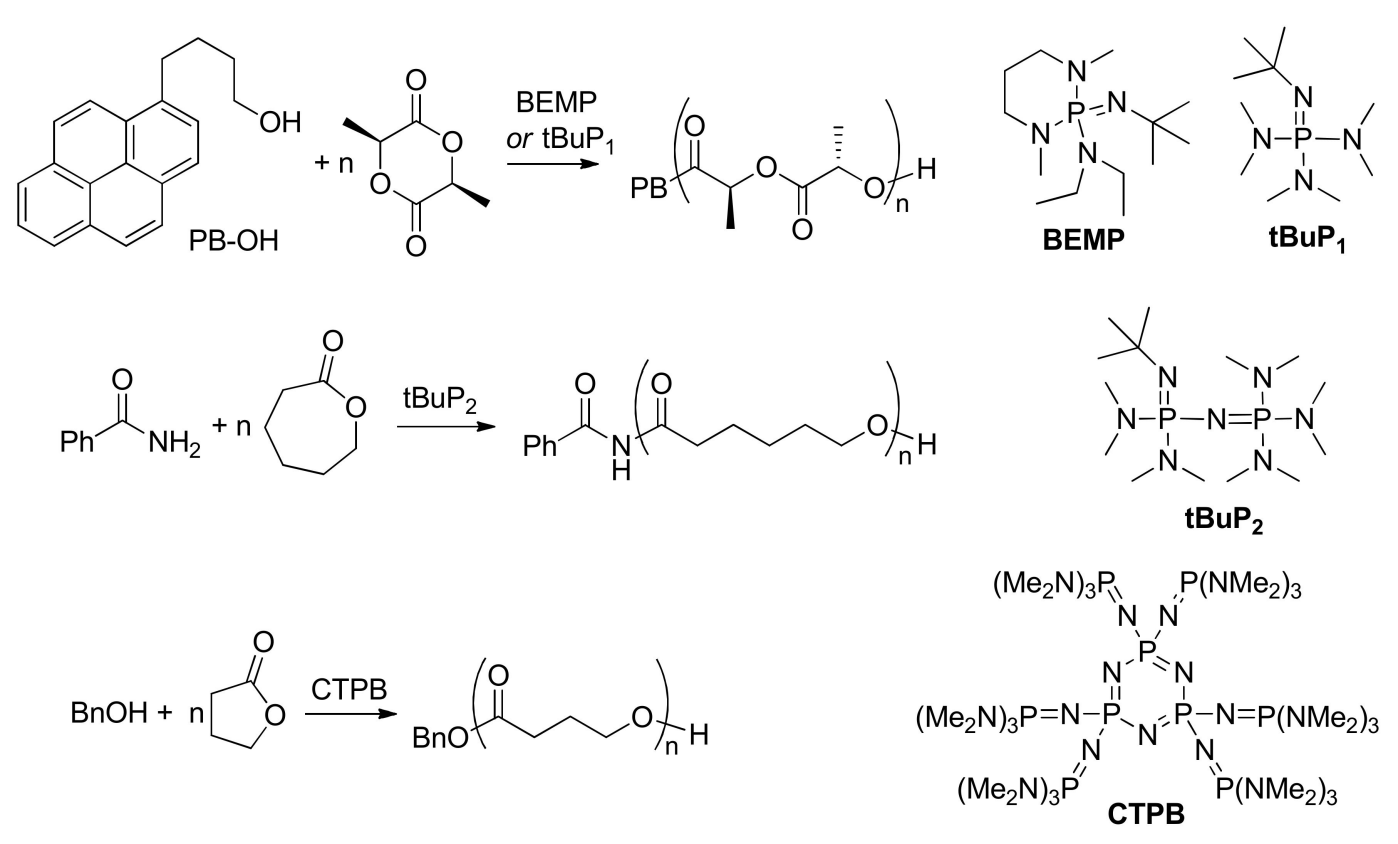
The examples of phosphazene-catalyzed ROP of cyclic esters.

A number of publications were devoted to the use of phosphazenes in ROP of cyclic esters [95,182,183,184,185,186,187,188,189,190]. For example, phosphazenes demonstrated high catalytic activity in δVL [182] εCL [182,185], *l*-LA [182] polymerization and block copolymerization of εCL with *l*-LA[182], being initiated by alcohols [182,185] and amides [185] as O–H and N–H acids, respectively (Scheme 13). Cyclic phosphazene (CTPB, Scheme 13) catalyzed living ROP of γBL [184] and macrocyclic ω-pentadecalactone [187]. The phosphazene-modified adamantane-biphenylene-based framework was effectively used in δVL and εCL polymerization [191]. Very recently, bifunctional (thio)urea-phosphazene catalysts were used in ROP of *d*,*l*-LA, δVL, and εCL [192].

Note that quantum chemical methods were successfully applied for the design of novel phosphazene catalysts in terms of Brønsted basicity [193,194]. We believe that DFT modeling could also be usefully extended to phosphazene-catalyzed ROP of different substrates.

## 10. Concluding Remarks

To date, different organocatalysts have been successfully used in the ROP of cyclic esters (actones, lactides, carbonates) and cyclic phosphates and phosphonates, which was the subject of this review. Starting from basic catalysts, such as DMAP and NHC, through the use of non-nucleophilic bases, such as DBU and phosphazenes, the researchers came to organocatalysts, providing the most gentle and energetically favourable donor-acceptor mechanism, namely, TBD—DBU/TU—thiourea and urea salts. Currently, the last group of the catalysts appears to be highly promising due to versatility [142] and knowingly lack of toxicity. The role of DFT modeling in understanding of the ROP mechanisms and in the design of the novel ROP catalysts is crucial, comparative theoretical estimations of the ROP activation barriers for zwitterionic and donor-acceptor mechanisms resulted in the development of efficient catalytic systems that are able to activate both cyclic ester molecule and chain-end fragment.

Different methods have been applied for the DFT modeling of the polymerization of cyclic esters. In Table 1, we summarized the technical aspects of DFT modeling of the organocatalytic ROP. The use of one or other of the alternative optimization methods depends on both research objectives (e.g., comparison of the alternative reaction pathways, understanding of the causes of stereochemical induction, etc.) and presentation purposes (visualization of the reaction complex in context of the proposed reaction mechanism). The use of solvation model in DFT calculations, which is essential for zwitterionic processes, seems to be of less importance for the reactions in non-polar media occurring via hydrogen transfer within the reaction complex. A manifold increase of calculation time, while using actual solvent model, poses a problem for choosing between the completeness of the model and accuracy of geometries and energies of the stationary points and transition states. Hence, the use of the solvation model is a choice of the researchers, particularly in view of the fact that the pre-exponential Arrhenius factor and conformational effects have been totally ignored by most of the researchers, given the staggering complexity of considering these factors in DFT calculations.

The main conclusion of the present review was to recognize that proton exchange and hydrogen bonding [195] play a determining role in organocatalytic ROP of cyclic esters, especially for ROH-initiated processes. This is the difference between coordination ROP [32] and organocatalytic ROP, as discussed in this review, with regard to DFT modeling. Such a difference between coordination and organocatalytic ROP also affects the character of the polymerization: for active coordination catalysts, the polymerization occurs as a living process, the polymerization grade *P_n_* is determined by the monomer/catalyst ratio. In organocatalytic ROP, *P_n_* is determined by the ratio of monomer and ROH initiator; therefore, the amount of the organocatalyst can be substantially minimized, as shown in Figure 1.

Besides, the traditional coordination-insertion ROP mechanism of the “living” polymerization implies the formation of linear polymers with narrow MWD if the activation barriers of ring-opening and transesterification substantially differ in energy. The zwitterionic mechanism becomes possible by using basic organocatalysts in the absence of proton donors, and DFT modeling compellingly demonstrates the relative ease of the formation of macrocyclic oligomers that are apparent polymerization products.

As can be seen, the results of DFT simulations of the reaction profiles are often unrelated with experimental data on catalytic activity in comparing different organocatalysts. Such a discrepancy can be attributed to the imperfections of the methods used in modeling (and usually the authors have been guilty of such an explanation). However, the direct correlations between activation energies and polymerization rates is not entirely correct, because ignoring the pre-exponential Arrhenius factor would not necessarily be equal for different catalysts. The role of the conformations of growing polymer chain, and other factors, facilitating or impeding the formation of catalytic complex, should be taken into account, especially for organocatalytic processes with relatively weak catalyst—monomer—activator interactions. Transesterification processes can become essential, notwithstanding their higher activation barriers if the transport of the substrate molecule is hindered, as was demonstrated in [109]. In view of the polymer architecture, such shift in the reaction might result in the formation of branched polymers in the case of ethylene phosphates’ ROP, and in broadening of MWD for common cyclic substrates, such as lactones and lactides. Moreover, in the course of ROP the shift from living to immortal polymerization, from chain-growth to step-growth reaction mode, can be occurred.

One of the main purposes for the use of organocatalysis in the synthesis of polyesters was to avoid metal complexes that may be toxic and difficult in the removal from polar polymers. At first glance, organocatalytic ROP allows for solving the problem of the purity of the materials attended for biomedical and microelectronics applications. DFT modeling that help us to understand the reaction mechanisms and, therefore, to outline the ways of the modern catalyst design, is a powerful tool in the development of novel catalysts and prospective materials. However, it should be noted that theoretical estimations of the applicability and effectiveness of one or the other organocatalyst is incomplete without the study of the toxicity of these catalysts [21] and, more pertinently, of the possibility of direct covalent bonding between organocatalyst and polyester molecules, and side transformations of cyclic substrate molecules and polyester macromolecules under the action of organocatalysts. Of course, DFT modeling is unable to assist researchers in the study of toxicity, which is the area of biomedical research [21], however, DFT methods may assist not only in understanding of the ROP mechanisms, but in excluding of the potentially undesirable reactions during polymer preparation and separation stages.

Polymer degradation and utilization is another prospective field for the application of DFT modeling. To date, only few publications are devoted to this issue [30,196,197].

The clear progress in computer engineering and developing of the methods of quantum chemistry allows for the consideration of the DFT modeling as a common and useful technique for understanding and visualization of polymerization mechanisms. It is fair to say that the potential of the DFT is not being fully utilized in the design of new catalysts, processes, and materials, and we strongly hope that this review will help researchers to expand their scientific tools by the use of this simple and efficient method.

## Figures and Tables

**Table 1:** DFT methods used in the modeling of organocatalytic ROP of cyclic esters.

Catalyst	Monomer	Functional	Basis Set	Solvation Model	Ref.
– ^1^	γBL, δVL	B3LYP	6-31G(d)	PCM ^2^	[37]
DMAP	*l*-LA	B3LYP	6-31G(d)	PCM/SCRF	[50]
adenine	*l*-LA	M06-2X	6-31+G(d,p)	n.d.^3^	[43]
Me_4_-NHC ^4^	δVL	B3LYP	6-311+G(2d,p), aug-cc-pVTZ	IEF-cPCM	[75]
Me_4_-NHC	εCL	B3LYP	6-311+G(2d,p), aug-cc-pVTZ	IEF-cPCM	[79]
abnormal NHC	εCL	BP86	SVP, TZVP	n.d.	[86]
TBD	δVL, βBL	B3LYP	6-31+G*	SCRF	[91]
TBD	*l*-LA	MPW1K	6-31+G	cPCM	[96]
TBD	ethylene carbonateTMC, γBL, δVL, εCL, PDO, GL	B3LYP	6-311G(d)	gas phase	[94]
TBD	macrolactone	M06-2X	6-31+G(d), 6-311++G(d,p)	n.d.	[100]
TBD	*d*-mannose-derived cyclic carbonate	rωB97XD	6-311++G(2d,p)	cPCM	[101]
TBD	TMC, 2-deoxy-*d*-ribose derived cyclic carbonates	rωB97XD	6-311++G(2d,p)	cPCM	[103]
TBD	MeOEP	B3PW91	DGTZVP	gas phase	[109,110]
guanidines	*l*-LA	B3LYP	6-31+G*	IEF-cPCM	[116]
guanidines	*l*-LA	B3LYP	TZVP	PCM	[117]
DBU	*l*-LA	M06	6-31+G(d,p)	cPCM	[123]
DBU/PhCOOH	*l*-LA	B3LYP	aug-VTZP, aug-cc-pVTZ	IEF-cPCM	[125]
amino-TU	*l*-LA	B3LYP	6-311++G(d,p)	SCRF-PCM	[130]
TU Na salt	*l*-LA	B3LYP-D3	6-31+G(d), aug-cc-pVTZ	IEF-cPCM	[134]
RNHC(O)NHR’	γBL	GGA	PBE	gas phase	[143]
Ph_2_P(O)OH	εCL	B3PW91	6-31G(d,p)	cPCM	[166]
CF_3_SO_3_H	εCL	B3PW91	6-31G(d,p)	cPCM	[173]
MeSO_3_H	TMC	M11	6-311+G(2d,p), aug-cc-pVTZ	IEF-cPCM	[175]

^1^ The estimation of the ROP thermodynamics. ^2^ PCM calculations performed at HF/6-311G(d,p) level. ^3^ No data. ^4^ 1,2,3,4-Tetramethylimidazole-derived carbene.
